# Gruffi: an algorithm for computational removal of stressed cells from brain organoid transcriptomic datasets

**DOI:** 10.15252/embj.2022111118

**Published:** 2022-08-02

**Authors:** Ábel Vértesy, Oliver L Eichmüller, Julia Naas, Maria Novatchkova, Christopher Esk, Meritxell Balmaña, Sabrina Ladstaetter, Christoph Bock, Arndt von Haeseler, Juergen A Knoblich

**Affiliations:** ^1^ Institute of Molecular Biotechnology (IMBA), Austrian Academy of Sciences Vienna Biocenter Vienna Austria; ^2^ Max Perutz Labs, Center for Integrative Bioinformatics Vienna (CIBIV) University of Vienna Vienna Austria; ^3^ Medical University of Vienna, Vienna Biocenter Vienna Austria; ^4^ Vienna Biocenter PhD Program A Doctoral School of the University of Vienna and Medical University of Vienna Vienna Austria; ^5^ Institute of Molecular Pathology (IMP) Vienna Austria; ^6^ CeMM Research Center for Molecular Medicine of the Austrian Academy of Sciences Vienna Austria; ^7^ Bioinformatics and Computational Biology, Faculty of Computer Science University of Vienna Vienna Austria; ^8^ Department of Neurology Medical University of Vienna Vienna Austria

**Keywords:** bioinformatics, ER‐stress, hypoxia, neural development, organoids, Methods & Resources, Neuroscience

## Abstract

Organoids enable *in vitro* modeling of complex developmental processes and disease pathologies. Like most 3D cultures, organoids lack sufficient oxygen supply and therefore experience cellular stress. These negative effects are particularly prominent in complex models, such as brain organoids, and can affect lineage commitment. Here, we analyze brain organoid and fetal single‐cell RNA sequencing (scRNAseq) data from published and new datasets, totaling about 190,000 cells. We identify a unique stress signature in the data from all organoid samples, but not in fetal samples. We demonstrate that cell stress is limited to a defined subpopulation of cells that is unique to organoids and does not affect neuronal specification or maturation. We have developed a computational algorithm, Gruffi, which uses granular functional filtering to identify and remove stressed cells from any organoid scRNAseq dataset in an unbiased manner. We validated our method using six additional datasets from different organoid protocols and early brains, and show its usefulness to other organoid systems including retinal organoids. Our data show that the adverse effects of cell stress can be corrected by bioinformatic analysis for improved delineation of developmental trajectories and resemblance to *in vivo* data.

## Graphical abstract

A unique stress signature found in in brain organoid samples but not in fetal samples can be quantified and removed from scRNAseq datasets using a new algorithm.

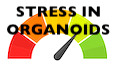

## Introduction

Organoids are 3D stem cell cultures that enable human tissue modeling with unprecedented structure and complexity (Eiraku *et al*, 2008; Kadoshima *et al*, 2013; Lancaster *et al*, 2013; Paşca *et al*, 2015; Qian *et al*, 2016). At the same time, single‐cell transcriptomics has become widely used for their characterization. Alongside these recent technological breakthroughs, it has become clear that 3D tissue culture is affected by limited oxygen and nutrient supply to the center of the tissue.

As most models lack functional vascularization (Garreta *et al*, 2021), and therefore rely on limited passive transport across the tissue, diffusion‐limited hypoxia is an intrinsic problem in organoids. Nutrient‐ and in particular oxygen‐limitation are long‐known phenomena in tissue models (Malda *et al*, 2007; Volkmer *et al*, 2008). Oxygen restriction causes widespread metabolic changes by activating the hypoxia‐, glycolysis‐, and ER stress‐ pathways; furthermore, it affects differentiation and proliferation (Kültz, 2005; Mohyeldin *et al*, 2010).

Brain organoids are among the most complex and physically largest organoids and are therefore most affected by the limited nutrient supply of the center (Qian *et al*, 2019). Nevertheless, this problem has only been recently addressed (Mansour *et al*, 2018; Giandomenico *et al*, 2019; Bhaduri *et al*, 2020; Qian *et al*, 2020), and its extent is still debated.

It therefore remains an open question if stress is a global or a local issue, thus, how much it affects the 3D tissue culture model. A recent paper claimed that *in vitro* conditions lead to a pervasive stress across the whole organoid, causing immaturity, misspecification, and dissimilarity to fetal tissue (Bhaduri *et al*, 2020). These observations contrast with the previous understanding of spatially limited stress (Qian *et al*, 2019). This raises the question: How should we handle the data affected by an artificial stress signature? It is unclear if stress is an “acute” signature on top of a cell's original identity, or if stress is leading to a completely different cellular state. While the same stress pathways are also active in the fetal brain, reports disagree on whether it is equivalent to those observed *in vitro* (Bhaduri *et al*, 2020; Gordon *et al*, 2021).

Experimental solutions emerged in protocols which increase convection with bioreactors, orbital shakers, or microfluidics. Despite these efforts, 3D cultures above ~ 500 μm radius develop a necrotic core with healthy tissue limited to the surface ~ 100–300 μm. Further developments involve organoid implantation *in vivo* resulting in subsequent vascularization (Mansour *et al*, 2018), *in vitro* induction of vasculature (Cakir *et al*, 2019), section culture (Giandomenico *et al*, 2019; Qian *et al*, 2020), or bioengineering solutions (Garreta *et al*, 2021). These approaches aim to increase nutrient supply, but neither is currently as scalable as the standard organoid culture, which therefore remains the mainstay of organoid research. Until a widely applicable and scalable experimental solution emerges, tissue health and cellular stress persist as a problem for the field.

While most large single‐cell RNA–seq studies on diverse brain organoid systems have reported glycolytic or ER‐stressed clusters (Kanton *et al*, 2019; Velasco *et al*, 2019; Tanaka *et al*, 2020), there is no consensus on how to identify them, what is happening in these cells, or what the effects of stressed cells on the organoid are. To measure the prevalence and consequences of stress in brain organoids, we analyzed differentiation, maturation, and identity of ~ 160,000 single cells from newly presented and published cortical and cerebral organoid (together: brain organoids) datasets.

We found stressed cells in all organoid samples, forming a distinct subpopulation. Beyond stress pathway activity, stressed cells showed widespread transcriptional changes that we refer to as the “stressed‐state.” We do not find this stressed state *in vivo*; therefore, it is likely an artifact. Eliminating artificial cell populations is essential to truly recapitulate *in vivo* conditions. As stressed cells are currently unavoidable, we developed *granular functional filtering* (Gruffi), an unbiased computational algorithm to isolate stressed cells. Gruffi can clarify developmental trajectories and increase similarity of *in vitro* datasets to the fetal brain.

## Results

### A distinct population of ER‐stressed‐ and glycolytic‐cells exist in all analyzed organoids

**Figure 1:**
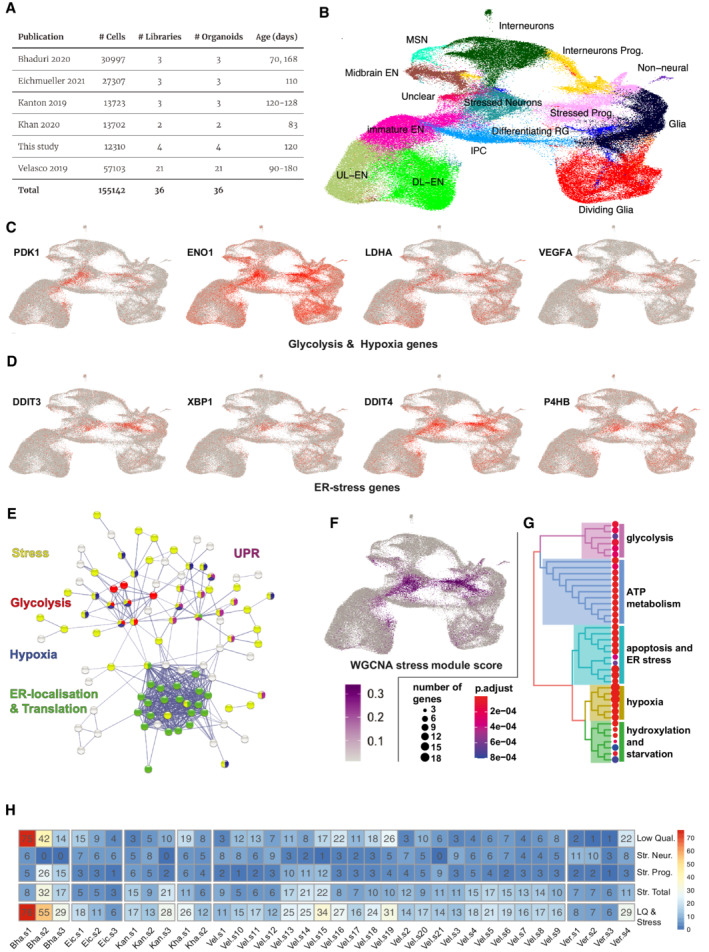
A distinct population of ER‐stressed‐ and glycolytic‐cells exist in all analyzed organoids A
The list of samples and datasets analyzed in this study encompasses mature cortical organoids from multiple key publications.B
UMAP embedding of the integrated dataset. Clustering with cell‐type annotation shows the expected neural cell types, but also reveals two stressed subpopulations.C, D
(C) Key marker genes for glycolysis, hypoxia or (D) ER‐stress are specifically enriched in stress clusters.E
Protein–protein interaction map of GO‐term enrichments on the top 150 stressed‐cluster enriched genes (by log fold change). Highlighted terms: Cellular response to stress (red, GO:0033554, 2e‐07, 0.47); Response to hypoxia (blue, GO:0001666, 3e‐10, 0.9); Response to unfolded protein (yellow, GO:0006986, 2e‐06, 0.97); Glycolytic process (limegreen, GO:0006096, 1e‐05, 1.36); Protein localization to endoplasmic reticulum (cyan, GO:0070972, FDR: 2e‐20, strength 1.35)—covering nearly the same ribosomal genes as: Translational initiation (GO:0006413, 2e‐20, 1.35).F, G
(F) WGCNA analysis (see Appendix Fig S1 for other modules) of variable genes identifies a gene module specific to stressed cells, (G) which is enriched in stress related terms.H
Percentage of low‐quality cells; cells in stressed‐neuron and ‐progenitor clusters and their union, quantified across all datasets.

We reanalyzed recent, landmark single‐cell transcriptomics studies and performed new experiments (Kanton *et al*, 2019; Velasco *et al*, 2019; Bhaduri *et al*, 2020; Khan *et al*, 2020; Eichmüller *et al*, 2022; Fig 1A) to answer three questions: What stress pathways are active in organoids? Does stress occur in all or only certain organoid protocols? Is cellular stress limited to a group of cells or is it pervasive?

We included all those mature, wild‐type samples that were prepared on the 10× Chromium single‐cell platform starting from .fastq files using the same pipeline (Materials and Methods). To focus on stress in the neural lineage, we removed all cells that are not part of brain development and are a result of mispatterning, sometimes observed in organoids. For a proportional representation of datasets, we subsampled ~ 160,000 from a total of 300,000 cells.

Cellular stress can lead to a perturbation of essential processes, thus affecting cell quality in scRNAseq. Therefore, we applied a minimal filtering, keeping all cells with > 500 genes, < 20% mitochondrial‐ and 30% ribosomal‐reads (Ilicic *et al*, 2016; Luecken & Theis, 2019). This resulted in a median depth of 3,651 UMI/cell (Materials and Methods). We integrated and analyzed the resulting datasets in Seurat (v4) and found the previously reported cell types (Fig 1B). The UMAP separated dividing cells and glia cells from neurons (horizontally) and excitatory‐ from interneurons (vertically). Besides, there were multiple clusters in the center of the UMAP, which were less well‐defined by marker gene expression.

### Stress is a common hallmark of the two largest unidentified clusters

**Figure EV1:**
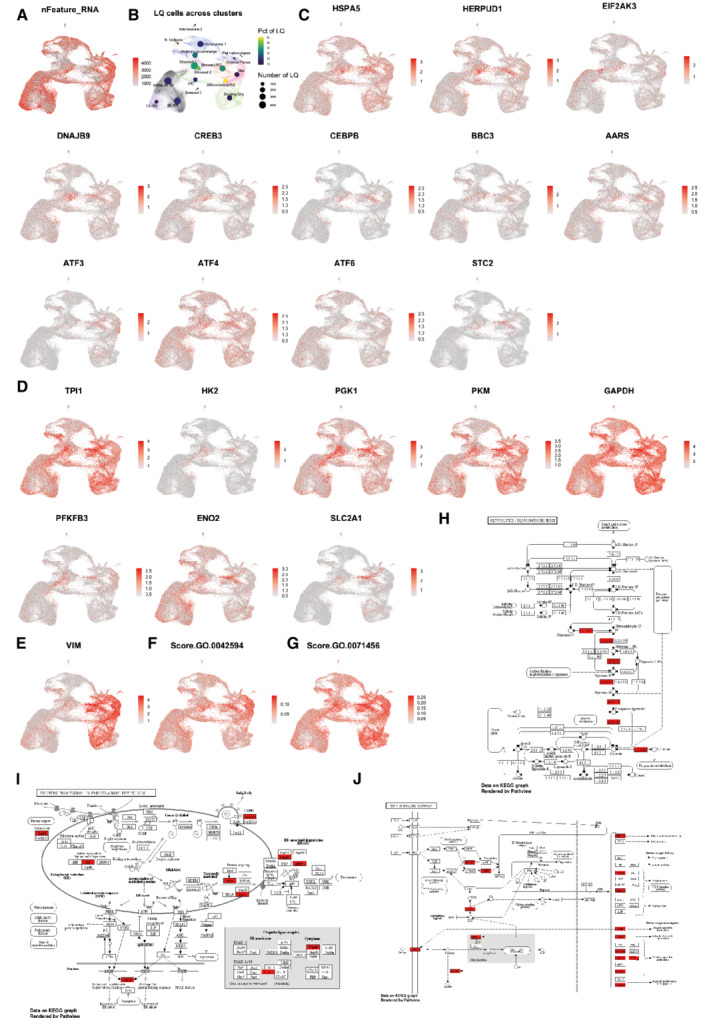
Metabolic changes and marker genes in stressed cells A
UMAP of organoid integration shown in Fig 1B colored by number of RNA features per cell (nFeature_RNA).B
Low‐quality (LQ) cells as determined by expression of < 1,000 features. In the background the clustering of Fig 1B is shown. On top per cluster the percentage of LQ cells per cluster (color) and the number of LQ cells (size) are depicted.C
Expression of additional endoplasmic reticulum (ER) stress genes enriched in the stress clusters.D
Expression of additional glycolysis genes enriched in the stress clusters.E
Vimentin (*VIM*) is expressed in all progenitor populations regardless of lineage or stress state.F, G
Additional GO‐terms scores ‘response to starvation’ (GO:0042594) and ‘cellular response to hypoxia’ (GO:0071456) are also characteristic of stressed cells.H–J
Stress cluster marker genes in relevant significantly associated KEGG pathways: HIF‐1α signaling, (Genes: 12, Fold Enrichment: 14.4, FDR: 2.30e‐9); Glycolysis, (7, 13.7, 2.3e‐5); Protein processing in the ER, (8, 5.7, 1.9e‐3). The top 150 coding stress marker genes were used for this analysis (as in Fig 1E). Enriched genes are marked red.

Differential gene expression and QC‐metric analysis revealed that the “unidentified” two central clusters (stressed neurons and stressed progenitors, Fig 1B) consisted of cells strongly expressing stress markers and low‐quality cells (Figs 1C and D, and EV1A and B). The stress genes were part of endoplasmic reticulum (ER) stress: *CHOP* (or *DDIT3*), *XBP1*, *DDIT4*, *P4HB* (Rashid *et al*, 2015); glycolysis (*ENO*, *HK2*, *PGK1*, *GAPDH*), and hypoxia: *PDK1*, *PHD*, *GLUT1* (or *SLC2A1*; Lee *et al*, 2020; Figs 1C–D and EV1C–J).

To better understand the nature of stress in these cells, we analyzed all significantly differentially enriched genes in the stress‐clusters (Materials and Methods). We found that, in either cluster, more than half of the top 50 coding genes were part of “response to stress” (GO:0006950), and apoptosis‐related terms were among the strongest enriched terms (Dataset EV1, Materials and Methods). The biggest enriched stress pathways were “regulation of cell death,” (GO:0010941) “response to hypoxia,” (GO:0001666) and “response to endoplasmic reticulum stress” (GO:0034976). Surprisingly, metabolic terms were both among the strongest and largest enriched terms, highlighting that metabolic shift is a hallmark of stressed cells in organoids.

We then calculated the GO‐term enrichment within the 150 strongest enriched coding genes of both stress clusters together and visualized these on the protein–protein interaction (PPI) map (Fig 1E, Materials and Methods). We highlighted enriched GO‐terms (FDR < 5e‐7) forming connected PPI clusters, revealing the interplay of glycolysis, hypoxia, unfolded protein response, and translation with the general stress response (Fig 1E, Dataset EV1). To identify all genes co‐regulated with stress, we applied scWGCNA (single‐cell weighted gene co‐expression network analysis (Morabito *et al*, 2021)) and found 12 gene modules (Appendix Fig S1A), one of which was specific to stressed cells (stress module, Fig 1F). Gene set enrichment analysis (GSEA) on the stress module identified the strongest enrichment for “response to hypoxia,” “cellular response to ER stress,” and GO‐terms of glycolytic processes further highlighting the relevance of these pathways in stressed cells (Fig 1G, Appendix Fig S1B). To test whether stress occurred in all samples, we quantified the contribution of each dataset to the stress clusters. We found that all datasets contained stressed cells with a median of 13%, but highly variable fraction (50% CV, Fig 1H). Thus, stressed cells are characterized by deregulation of defined pathways and a general feature of organoids regardless of conditions, laboratory of origin or protocol used.

While the initial clustering‐based approach identified cells with stress signatures, it had three major limitations. First, while some clusters are too large and comprise mixed populations (Appendix Fig S1C), others can be too small to find marker genes by differential gene expression analysis (DGEA). Second, cluster boundaries are often not well‐defined, especially when dealing with developmental trajectories. Third, the resulting limitations in DGEA obstruct the identification of stress genes, so that results vary by the dataset and parameters used. Together, these limitations affecting DGEA could explain why previous studies identified disparate gene sets, such as “Glycolytic cells” in (Nowakowski *et al*, 2017; Kanton *et al*, 2019) vs. “ER stressed cells” in (Bhaduri *et al*, 2020; Tanaka *et al*, 2020). To overcome these issues, we tested different clustering resolutions (Appendix Fig S1D). As none of these could separate the distinct populations of cells within “Stressed Neurons” (Fig 1B), we concluded that a new approach is needed to identify and exclude stressed cells.

### Granular functional filtering identifies stressed cells unbiasedly

#### Functional scoring highlights cellular stress regardless of cluster boundaries

**Figure 2:**
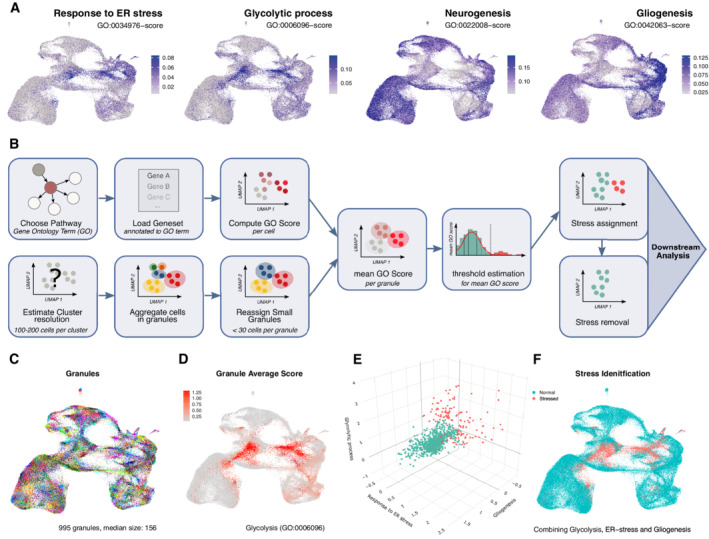
Granular functional filtering identifies stressed cells unbiasedly Granular functional filtering identifies stressed cells unbiasedly.A
Gene‐set scores per cell for the two strongest stress signatures (ER‐stress and glycolysis) and the two cardinal processes in the developing brain (neurogenesis and gliogenesis). The complementary expression signatures suggest a mutually exclusive neural‐ or stressed‐ fate.B
Overview of Gruffi's stress classification. After preprocessing steps including the computation of PCA and UMAP embeddings, a gene ontology pathway is selected, respective gene sets are retrieved, and per cell GO‐scores are calculated. At the same time, an ideal clustering resolution is estimated, such that cells are assigned to granules of (in median) ~ 100–200 cells, and small clusters (< 30) are reclassified. Next, to overcome high variability and detection noise caused by single‐cell resolution, average and cell number normalized granule scores are calculated, and respective score‐thresholds are estimated based on the score's dispersion. Finally, stressed granules are identified by a combination of scores, and isolated from the dataset for separate analysis or dataset cleaning and further downstream analysis is possible.C
Gruffi defined 995 granules by snn‐clustering containing a median of 156 cells.D
Granule scores for glycolysis shown on UMAP.E
Three‐dimensional stress score threshold estimation by Gruffi using default setting, requiring high glycolytic and ER‐stress, low gliogenesis score to define stressed cells.F
Stressed cells classified by Gruffi based on ER‐stress, glycolysis and gliogenesis are highlighted on the UMAP.

To universally identify stressed cells, we established a sample‐ and data‐independent definition for stressed genes. Using gene lists from well‐characterized pathways defined as GO‐terms (Materials and Methods), we aggregated information from all genes per pathway by an expression‐scoring method widely used for cell cycle scoring (Tirosh *et al*, 2016). Therein, we downloaded gene lists per GO‐term from Ensembl, calculated their average expression, and normalized it to randomly sampled control genes of matching expression level (Materials and Methods). Finally, we evaluated whether functional scoring helps to characterize stressed cells. We found that high scores for “glycolytic process” (GO:0006096) and “response to endoplasmic reticulum stress” (GO:0034976) were the strongest signatures of stress‐clusters (Fig 2A) and provided clearer separation between stressed and non‐stressed cells than cluster boundaries. Importantly, high scores marked mostly overlapping cell populations. The coactivation of additional scores, such as “response to starvation” (GO:0042594, Fig EV1F) and “cellular response to hypoxia” (GO:0071456, Fig EV1G) corroborated a complex stress‐identity. Comparing neuronal and glial cell types revealed that all nondividing glial cells showed higher ER stress scores (Fig 2B). We therefore designed our algorithm to accommodate for cell‐type‐specific background when identifying stressed cells.

#### Change of cellular identity in stressed cells

Besides increased stress‐gene expression, stress clusters were characterized by low expression of pan‐neural markers (*NEUROD6*, *DCX*, *MAP2*, *NCAM1*, and *ELAVL4*; Appendix Fig S1E). To test whether this marker depletion is also reflected by a general change in glial or neural fates, we extended our scoring approach. We calculated scores for the two cardinal cell states in neural development, “neurogenesis” (GO:0022008), and “gliogenesis” (GO:0042063). Both terms were depleted in stressed cells (Fig 2A). Compared to both glial and neural clusters, stressed cells also showed remarkably low scores for “cell differentiation” (GO:0030154), and “forebrain development” (GO:0030900), suggesting that stressed cells are in a metabolic survival state characterized by a lack of specification to neurons or glia (Appendix Fig S2A and B). Thus, chronic stress in organoids comes at the expense of neurogenic cell differentiation and leads to an undifferentiated, metabolic, and stressed cell‐state that we refer to as the “stress identity.”

#### Granular evaluation overcomes noise inherent to single‐cell data

Single‐cell gene expression measurements are inherently noisy. While GO‐scores are computed across multiple genes per cell, these may still suffer from high variability and noise. Indeed, some cells within the stress‐clusters showed low stress‐scores, even if clustering together (Fig 2A). At the same time, sporadic cells in well‐defined cell types showed high stress‐scores. These cells expressed stress genes inconsistently, but expressed respective cell‐type markers, which are otherwise absent in stress‐identity cells.

To overcome variability in single‐cell measurements, one can either denoise the data, for example, by model‐based imputations, or group cells and evaluate them together. Many different imputation methods have been developed recently; however, imputed values often vary (Hou *et al*, 2020), and they can induce false signals (Andrews & Hemberg, 2018). This is probably due to the complexity of the imputation problem. We therefore took a grouping approach where we partitioned cells into groups of 100–200 cells by ultrahigh‐resolution SNN‐clustering in PCA‐space (Materials and Methods) resulting in small groups of cells that we term *granules*.

The ultrahigh‐resolution clustering approach can overcome the problems of boundaries by breaking down the data into minute groups of cells. To get sufficient coverage for robust gene scoring, and because clustering creates some very small granules, we added a reclassification step, where cells in granules with < 30 cells are reassigned to the closest granule above threshold (Materials and Methods).

To test the granular approach, we compared stressed cells identified by Gruffi's granular method (gSC) and stressed cells identified on single‐cell scores (scSC). We contrasted cells only identified by either, both or neither of the approaches, thus validating whether top stress markers alone is superior in filtering stressed cells. scSC were evenly scattered across all clusters, while most gSC were close to the previously identified stress clusters and the cells identified by both methods (Appendix Fig S2C). By definition, single‐cell selection on stress scores identified the cells with highest stress‐scores. Thus, we tested whether the scSC‐only selected cells had other hallmarks of stressed cells. scSC cells showed less of all other features defining stress‐identity: lack of cell differentiation, lower mitochondrial, and higher ribosomal mRNA content (Appendix Fig S2D–I). In contrast, granular identification found cells that shared these features with cells identified by both methods and showed strong stress‐identity (Appendix Fig S2D–I). Thus, single‐cell scoring based on top stress markers alone is not sufficient to identify stressed cells. While the intersection of both scSC and gSC classification showed strong stress‐identity, this was also the case for gSC‐only cells. We implemented both methods in Gruffi, but we concluded that the granular approach is more suitable if one aims to exclude stress‐identity, whereas the single‐cell approach is more suitable if one aims to simply find cells with the highest stress gene expression, but otherwise properly specified cells.

#### Granular functional filtering (Gruffi) isolates and removes stressed cells

As clustering‐based identification failed to detect stressed cells specifically and robustly, we built on the concepts above and developed *granular functional filtering* or Gruffi (Fig 2B). Gruffi takes a number of gene ontology pathways (1) to obtain corresponding gene sets (2), and computes cell‐wise GO scores (2). At the same time, it identifies a suitable resolution by parameter search (I), clusters cells into granules (small clusters) (II), and reassigns cells of too small granules (III). Merging these, it then computes the multiple granule scores (4), estimates a threshold separating stressed and non‐stressed cells (5) and assigns a “stress” label integrating multiple scores (6).

To uniformly determine the prevalence of stressed cells across organoids and protocols, we applied Gruffi to the integrated organoid dataset. After pathway scores calculation, Gruffi identified the clustering resolution where the median cluster size is 154 cells, resulting in 995 granules, after reassignment (Fig 2C). Stress identification must be robust across all datasets; therefore, we incorporated three scores: the two most specific pathways: “glycolytic process” (GO:0006096) and “response to endoplasmic reticulum stress” (GO:0034976), and a negative filter on “gliogenesis” (GO:0042043) score accounting for the higher native expression of ER genes in glia. The addition of further negative filters did not improve identification; however, we implemented this option in our algorithm.

Gruffi then calculated the average and cell number normalized functional scores per granule resulting in a 3‐dimensional functional score for each granule (Fig 2D–E). This combinatorial approach is flexible to the type and number of scores used, which may be useful for applications beyond its original scope. Next, Gruffi estimated the thresholds separating stressed from normal cells, accounting for score variability among non‐stressed cells (Materials and Methods). At this point, the retrieved thresholds can and are advised to be inspected and refined via the implemented interactive Shiny App interface. Throughout our analysis presented here, we did not adjust manually the thresholds estimated by Gruffi. Finally, combining all thresholds, Gruffi classified stressed and non‐stressed cells (Fig 2F), which largely overlapped with the expression of key stress markers, and with high stress scores (Fig 1C).

Finally, we investigated how Gruffi compares to simple approaches for quality control. Therein, we looked at the effect of excluding cells based on different thresholds of low UMI‐count, high mitochondrial, or high ribosomal UMI‐fractions (Materials and Methods). These metrics are often used to exclude low‐quality, dying, and outlier cells. While low‐read count cells were enriched among stressed cells, this metric was not sufficient to remove stress‐identity cells, and it resulted in biased depletion of specific cell types at higher stringency (Appendix Fig S3A–C). Similarly, high mitochondrial or ribosomal UMI‐fractions were not specific to stressed cells. The mitochondrial cutoff is often used to filter dying cells. The lack of overlap with stressed cells suggest that hypoxic stress does not lead to cell death detectable by scRNAseq. As for read count, strict filtering thresholds either removed or depleted other specific cell types (Appendix Fig S3D–I). Thus, simple quality control metrics were not sufficient to remove stressed cells. While this suggested that Gruffi was superior in identification of stress‐identity cells, we continued the in‐depth analysis of stress in organoids.

### Stress‐identity is restricted to a subpopulation, which is not present *in vivo*

#### Stressed cells show a profound transcriptomic change

**Figure 3:**
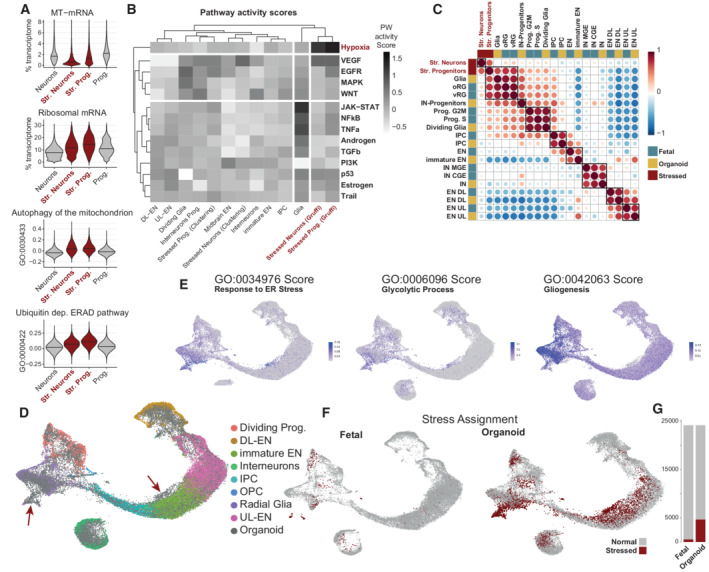
Stress has a profound, yet limited transcriptional effect, and stress‐identity is not present *in vivo* A
Functional consequences of stress on the transcriptome. Mitochondrial (MT) mRNAs were decreased (top), while ribosomal mRNA were increased in stressed neurons and progenitors (second from top). Mitochondrial autophagy was increased in stressed cells, possibly explaining the reduced MT‐mRNA (third from top). Increased protein degradation via the ERAD pathway in the ER is a likely reason for increased ribosomal reads. All differences were significant on Kruskal–Wallis test (*P* = 0) followed up by a Bonferroni corrected Dunn's test (*P*.adj < 1e‐100 for all, except %ribo: Prog. vs. Str. Neurons; *P*.adj < 5e7). Group averages and relative changes quantified in Dataset EV4.B
Activity of multiple pathways defined by PROGENy clearly separates stressed cells. Hypoxia is the only uniquely activated pathway in stressed cells. Hierarchical clustering based on pathways independently validates that stressed clusters as identified by Gruffi are distinct from other cell types. Stressed neurons and progenitors were divided into cells that are part of the stress cluster and identified by Gruffi [Stressed N./P. (Gruffi)], or cells that are part of the stress cluster but not identified in Gruffi (Clustering).C
Correlation of fetal reference to organoid clusters. Only clusters that are found in both datasets and stressed cells are shown. Gene modules of co‐regulated genes were computed in the fetal reference data. Aggregated expression per cluster and module was correlated. The color code marks the origin of the clusters (blue for fetal and yellow for organoid). Stressed neuron and progenitor clusters are marked in red. Fetal clusters correspond to original clusters with adjusted names (Polioudakis *et al*, 2019).D
CCA Integration of ~ 24 K fetal cells and an equal number of randomly sampled organoid cells from Fig 1B show that most cell types intermingle. Cluster annotation represents the original annotation of the fetal dataset. Gray points represent organoid cells.E
Gruffi's single‐cell pathway scores for ER‐stress (GO:0034976), glycolytic process (GO:0006096) and gliogenesis (GO:0042063) on UMAP. Granule clustering at resolution 37 (determined by Gruffi), resulting in 249 granules with a median of 193 cells per granule, after reassignment of small clusters.F
Stressed‐cell assignment by Gruffi identifies the vast majority of stressed cells in organoid samples.G
Quantification of F. In total, 5,171 cells (10.68% of all cells) were identified as stressed. Five hundred and three of these are fetal (2.16% of fetal cells) and 4,648 cells are from the organoid datasets (19.2% of organoid cells).

As all samples had stress‐identity cells, we searched for their defining features and their consequences on the whole organoid. Stressed cells fell in two distinct clusters with either a more glial or a more neuronal character (Fig 1). We therefore divided Gruffi's stressed cells into these two categories to investigate the heterogeneity of stress response in organoids (Materials and Methods). We quantified the total expression of mitochondrially encoded genes and of ribosomal mRNAs, which correspond, respectively, to ~ 2% and ~ 10% of the total transcriptome, respectively. These are widely used to assess quality and cell state in scRNAseq (Luecken & Theis, 2019). We hypothesized that chronic hypoxia and glycolysis diminish the need for oxidative phosphorylation, which may translate to fewer mitochondrial UMIs. Indeed, stressed neurons showed 52%, whereas stressed progenitors showed a 25% reduction in mitochondrial read fractions, as compared to their non‐stressed counterparts (Fig 3A). In contrast, ribosomal mRNA fractions were 40% and 23% higher, respectively (Appendix Table S1), perhaps to compensate for ER‐dysfunction (Bonferroni corrected Dunn's test *P*.adj < 5e‐7 in all cases).

#### Increased catabolism in stressed cells

As stressed cells had a decrease in mitochondrial mRNAs, we looked for transcriptomic signatures for the active degradation of mitochondria. To that end, we applied Gruffi's scoring method for relevant GO terms, in four broad categories of cells (Appendix Fig S4A) and found opposite trends: Both stressed populations (progenitors and neurons) showed on average positive scores for “autophagy of the mitochondrion” (GO:0000422), while normal cells did not (Fig 3A, *P*.adj < 1e‐100 in all cases). At the same time, the groups scored similarly for “autophagy” (GO:0006914; Appendix Fig S4B and Table S1).

Translation in ER‐stressed cells might lead to protein degradation via the ubiquitin‐dependent ERAD pathway. Therefore, we calculated the corresponding score (GO:0030433), and as for mitophagy, found that stressed cells scored positively, while normal cells scored negatively (Fig 3A, Appendix Table S1). Altogether, these changes show that stress induces major changes in the cell's physiology and metabolism that go beyond acute stress response.

As a complementary approach to GO‐term defined pathway scores, we turned to PROGENy, which derives pathway‐responsive genes from a large compendium of perturbation experiments and assigns a *Z*‐score normalized activity to each pathway (Schubert *et al*, 2018). Therein, we analyzed cell types by clustering PROGENy's activity‐score across signaling pathways and clusters. We found that stressed cells form an outgroup and are marked by the upregulation of Hypoxia and VEGF pathways, and the downregulation of the PI3K pathway, highlighting oxygen deficiency and quiescence (Fig 3B, Appendix Fig S4C–E, Materials and Methods). To ask how stressed cells identified by Gruffi differ from those detected by the naïve clustering‐based approach (Fig 1), we subcategorized the stressed clusters into cells identified by Gruffi or cells in the stressed clusters but not classified by Gruffi. The complete lack of separation of cells found by only the naïve approach contrasted the salient stress features found by Gruffi (Fig 3B). This suggests that a clustering‐based approach is impractical for excluding stressed cells as it is variable between datasets and would exclude non‐stressed cells, further supporting the versatility of Gruffi.

#### The presence of stressed cells does not affect specification and maturation of non‐stressed neurons

A previous study reported that stress in organoids leads to impaired cell‐type fidelity, and incomplete maturation as a global phenomenon in organoids (Bhaduri *et al*, 2020). To test those effects, we compared cell types in the organoid datasets to fetal cortical data of comparable age (de la Torre‐Ubieta *et al*, 2018; Polioudakis *et al*, 2019; Materials and Methods). We defined the fetal brain as the reference data and constructed modules from co‐expressed genes (Dataset EV2, Materials and Methods) as described before (Trapnell *et al*, 2014). The resulting 65 aggregated gene modules were used to calculate Pearson's correlation across clusters and visualized in a heatmap (Fig 3C). Major cell types (excitatory neurons, interneurons, and progenitors) formed the largest clusters across the dataset. Most organoid cell types pairwise best matched the corresponding fetal cell type, indicating that organoids undergo proper cell‐type specification unlike suggested previously (Bhaduri *et al*, 2020). Stressed neurons, however, were uncorrelated to all cell types, except stressed progenitors (Fig 3C). This dissimilarity to all fetal and organoid cell types, along with the analyses above, indicated that stress neurons lost most of their identity and formed a new transcriptional cell‐state. Interestingly, while stressed progenitors showed a similarity to stressed neurons, they also showed a strong progenitor identity, suggesting either an increased robustness or more distinct transcriptome of the progenitor state. Altogether, we found no evidence of impaired cell‐type fidelity in organoids, as cell types in organoids match respective cell types *in vivo*, and that stressed cells show little resemblance to cell types found in the fetal cortex.

#### Stressed cells in organoids have no fetal counterpart

Stressed cells might also exist *in vivo*, even if they did not form a recognized cluster in published studies. While some previous reports have argued that stressed cells similar to organoids exist *in vivo* (Tanaka *et al*, 2020; Gordon *et al*, 2021), others claim that it is an artifactual population specific to organoids (Bhaduri *et al*, 2020). Therefore, we integrated the fetal brain dataset with a matching number of randomly downsampled cells from the organoid dataset. Using the published fetal cell‐type annotation, we found that the organoid dataset was generally well recapitulating the fetal data (Fig 3D). At the same time, CGE and MGE (caudal and medial ganglionic eminences) interneuron differences and deep layer excitatory neuron differentiation were clearer in fetal data.

Interestingly, there were two populations entirely of organoid origin: a population near the neural trajectory (I) and a progenitor population (II, Fig 3D). To test for stress identity, we calculated stress scores as before, and found that precisely these populations score high for ER stress and glycolysis (Fig 3E). To quantify stressed cells and validate our method on both datasets, we ran Gruffi on the integrated object. This identified stressed cells almost exclusively in the two aforementioned populations and almost exclusively in organoid samples (Fig 3F–G). Notably, the few stressed cells identified *in vivo* did not share the characteristic enrichment of hypoxia that was found in organoid cells (Appendix Fig S4F). This suggests that stress‐identity cells are specific to the *in vitro* culture condition.

**Figure EV2:**
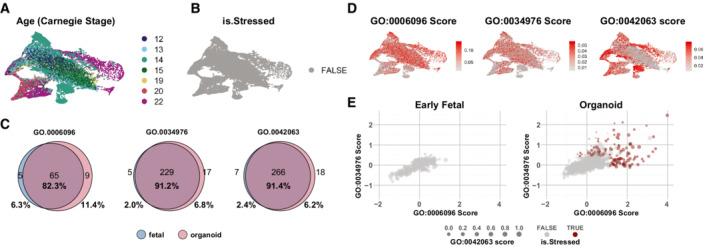
Early radial glia are not stressed A
UMAP of early radial glia cells from (Eze *et al*, 2021) color‐coded for age in Carnegie stages.B
Gruffi stress‐classification on early radial glia does not detect any stressed cells.C
Comparison genes expressed with the GO‐terms used for stress classification. The large overlap between fetal and organoid datasets shows that it is not a difference in expressed genes that underlies the lack of stress identified in Eze *et al*, thus, excluding a technical or sequencing depth bias in GO scores.D
Per‐cell glycolysis, ER‐stress and gliogenesis scores.E
Per‐granule scores for glycolysis (*x*) and ER‐stress (*y*) in early fetal and organoid datasets. Stress scores are generally low in the fetal sample, whereas in organoids, stress‐classified granules are outliers with high scores. Each granule is sized by the expression of the gliogenesis score, which reveals that individual clusters with high glycolysis and ER‐stress that were not classified as stressed in the organoid are indeed gliogenic.

Our analyses focused on mature organoids, in which hypoxia is a well‐known problem. To test whether similar stress is indeed not present in the human brain, we additionally investigated published samples of early brain development (Fig EV2A, Carnegie Stage 12–22; Eze *et al*, 2021). Gruffi classification revealed that there were no stressed cells during early stages of development (Fig EV2B), whereas the GO term's genes were still detected (Fig EV2C). Notably, there was no population that was uniquely enriched in stress GO terms, and overall scores were low (Fig EV2D and E). Thus, stress is neither found in early radial glia nor at later stages during development. Altogether, our analysis suggests that stress‐identity is an *in vitro* artifact, and there is minimal to no stress‐identity *in vivo*.

### Stressed cells do not affect the maturation of other cells, but their removal improves data quality and interpretability

#### The removal of stressed cells reveals clear trajectories that recapitulate fetal neurodevelopment

Removing stressed cells might create a better model of the fetal brain development. Stress genes majorly contribute to variable genes, which determine both clustering and visualization (Luecken & Theis, 2019). In the organoid integration dataset, 22 from the top 50 and 36 from the top 100 variable genes were enriched in “Response to stress” (GO:0006950; FDR: 3.4e‐3 & 2e‐3). We therefore removed all stressed and low‐quality cells, reidentified variable genes, and recalculated all dependent representations (PCA, UMAP, snn‐graph) and downstream analysis with identical parameters.

**Figure 4:**
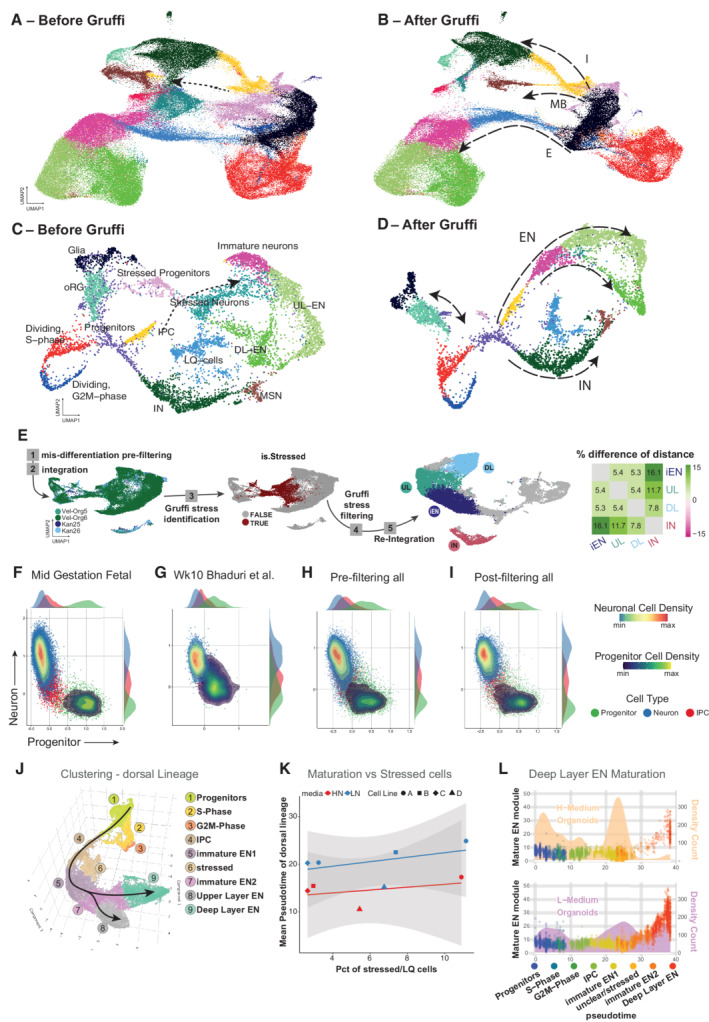
Stressed cells do not affect the maturation of other cells, but their removal improves data quality and interpretability A, B
UMAPs of the integrated organoid dataset before Gruffi (A), and after Gruffi (B). Dotted arrow in (A) highlights the broken trajectory in the development of midbrain cells. Dashed arrows in (B) highlight the developmental trajectories of interneurons, midbrain neurons, and cortical excitatory neurons. Clustering and colors are the same as in (Fig 1A).C, D
We repeated the analysis on a smaller subset of the data downsampled to ~ 8,200 cells, a typical outcome of a single 10× experiment (this subset does not contain midbrain neurons). Note that almost any lineage relationship could be inferred from the representation before Gruffi (A), but not the IPC to excitatory neurons relationship (dotted line). After Gruffi (B) the lineage trajectories show the known relationships allowing, for instance, pseudotime calculations.E
Exemplary workflow of Gruffi in dataset integration and analysis on the subset of datasets analyzed in Appendix Fig S5: after mis‐differentiation pre‐filtering (1) cells are integrated (2). Gruffi identifies stressed cells (3), that are removed (4). After re‐integration (5) the separation of cell types of interest improves, as evidenced by an increase in the distance between cell types (heatmap).F
Progenitor‐ (*x*‐axis) vs. excitatory neuron‐ (*y*‐axis) scores on mid‐gestational fetal cells (Polioudakis *et al*, 2019, Fig EV5B) identifies two separate populations. Each cell is colored based on neuronal or progenitor cell cluster identity. Color depicts progenitors (green), intermediate progenitor cells (IPC, red) and neurons (blue). In addition, the density of progenitors (blue to yellow) and neurons (blue to red) is shown. The margins of the plot depict the density distribution of the three different cell types across the progenitor (*x*‐axis, top) and neuron (*y*‐axis, right) score.G
Example dataset showing impaired cell subtype specification, as described in a previous study (Bhaduri *et al*, 2020).H
Subtype specification of all datasets analyzed in this study. While most neurons and progenitors show high values of the respective scores, there are some cells without specification to either neurons or progenitors (see Fig EV5C‐F for individual datasets).I
Subtype specification after filtering out stressed cells. The remaining cells specify properly to neurons and progenitors, with only IPCs localizing between the two populations (see Fig EV5C‐F for individual datasets).J
Clustering of dorsal lineage cells of datasets grown in two separate media conditions (Materials and Methods). Pseudotime analysis was performed from progenitors (cluster 1) to upper layer (cluster 8) and deep layer neurons (cluster 9, see Fig EV5G for pseudotime plot).K
The maturation along pseudotime is measured as the mean pseudotime value of neurons (*y*‐axis) and plotted against the percentage of stressed cells identified in the datasets (*x*‐axis). The color code shows the two different media formulations of individual datasets (marked by different symbols) and the linear regression model (line with CI). The percentage of stressed cells did not correlate with the maturation difference of non‐stressed neurons.L
Maturation of deep layer excitatory neurons (DL‐EN) in two different media formulations. Cells are color coded for clusters (J) and plotted along the pseudotime (*x*‐axis). The density‐count of cells along pseudotime is shown behind the dots (yellow area for H‐medium, purple area for L‐medium, right *y*‐axis). The position of the points reflects the expression of a module of co‐regulated genes enriched in DL‐EN (left *y*‐axis, Fig EV5H). While proper maturation indicated by expression of the DL‐EN module occurs in both media conditions, in L‐medium maturation is much more frequent (see Fig EV5I‐K for upper layer ENs and for individual datasets).

Starting from the progenitors, the resulting UMAP revealed three trajectories (“E,” “I,” and “MB” in Fig 4A and B), representing cortical excitatory, cortical inhibitory, and midbrain neurons, respectively. Before removing stressed cells, no midbrain trajectory was visible although midbrain cells were obviously present (Fig 1B). Instead, midbrain cells were linked to their progenitors only by a small, separated population of the “yellow” cluster. After stressed cell removal, these trajectories now lead to distinct populations of mature neurons, as opposed to the continuum of connected clusters before Gruffi (before stress removal). Notably, this lineage separation recapitulates fetal neurodevelopment.

**Figure EV3:**
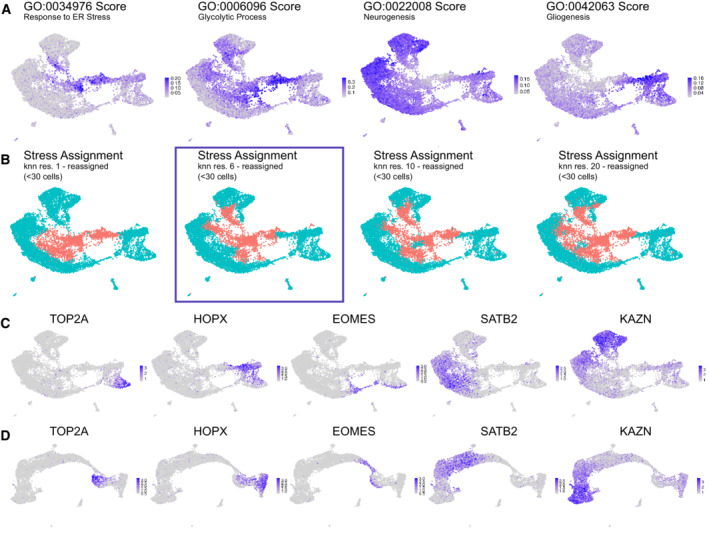
Benchmarking Gruffi on a single‐experiment sized dataset A
UMAP plots of one dataset (Velasco 7) cell‐wise GO score for response to Endoplasmic Reticulum stress (GO:0034976), glycolytic process (GO:0006096) and gliogenesis (GO:0042063).B
Stress assignment performed on k‐nearest‐neighbor clustering with resolution 1, 6, 10 and 20 plus reassignment of granules with a cell count below 30 (left to right). Resolution 6 (+ reassignment, the proposed resolution for a median cell number between 100 and 200 cells, see Materials and Methods), resulted in 53 granules with a median cell number of 197.C, D
Expression profiles of markers for progenitor cells (*TOP2A*, *HOPX*), Intermediate Progenitors (*EOMES*), upper layer excitatory neurons (*SATB2*) and deep layer excitatory neurons (*KAZN*) show that the developmental trajectory is refined in a newly computed UMAP after stress filtering (D) compared to before stress filtering (C). For the new UMAP, we recomputed (and scaled) the most variable genes, Principal Components and the UMAP embedding.

To ensure the robustness of our approach, we repeated the analysis on a single dataset (Fig EV3A–D), as well as downsampling the full dataset to ~ 8,200 cells, which is the typical output of a single 10× experiment (Fig 4C–D). Both analyses revealed that expected lineage trajectories are missing or broken in the UMAPs before Gruffi (midbrain in Fig 4A, and excitatory neurons in Fig 4C), but they are correctly recovered after running Gruffi (B, D). Correct and continuous trajectories in low‐dimensional representations are essential for most pseudotime methods that use these as basis for pseudotemporal cell assignment.

Prior to integrating organoid datasets, non‐telencephalic cells found in some organoids had to be removed (Materials and Methods). To investigate the relationship between properly specified telencephalic lineages, non‐telencephalic mis‐differentiation, and stressed cells we reanalyzed individual examples and the effect of integration (Appendix Fig S5). Non‐telencephalic cells were very distant from the neuronal lineage and could be easily distinguished in individual datasets (Appendix Fig S5A–C). However, combining mis‐differentiated samples with pure telencephalic datasets resulted in the artificial merging of these rare unwanted cell types with glia, demonstrating the need for prior cleaning (Appendix Fig S5D–F). In contrast to non‐telencephalic cells, stressed cells were more closely related to the neuronal lineage. This aligns with a model in which non‐telencephalic cells fail to undergo neural induction early during organoid differentiation, whereas stressed cells emerge during telencephalic differentiation at later stages (Appendix Fig S5G–I). To ensure proper integration and cleaning, individual datasets should first be evaluated, and mis‐differentiated cells must be removed. Then, stressed cells can be detected and filtered out after integration. This results in an improved cell‐type separation as evidenced by a larger distance between terminally differentiated cell types (Fig 4E, Appendix Fig S5J–L).

**Figure EV4:**
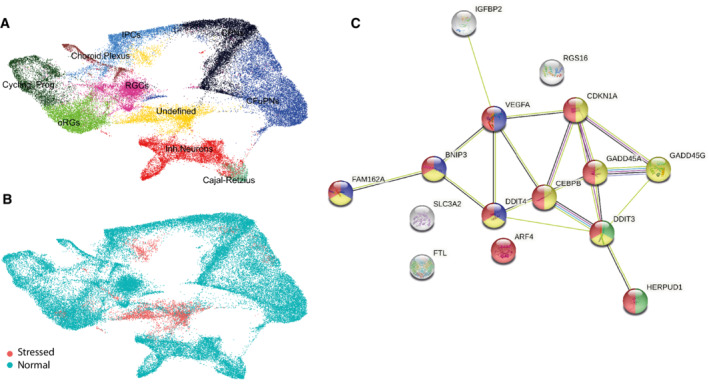
Stress identification in Samarasinghe *et al* (2021) A
Reproduction of Fig 4A UMAP in (Samarasinghe *et al*, 2021).B
Stress assignment by Gruffi using res.25 (auto determined resolution, 260 granules).C
Protein interaction map of marker genes of stress cells. Response to stress (red, GO:0006950, 0.02); Apoptotic process (yellow, GO:0006915, FDR = 0.0003); PERK‐mediated unfolded protein response (limegreen, GO:0036499, 0.0221); Response to hypoxia (blue, GO:0001666, 0.0334); DGEA and all enrichment terms are in (Dataset EV5).

To test whether our method can be applied to an independent dataset, we reanalyzed the data from a recent publication reporting a large “undefined” cluster (Samarasinghe *et al*, 2021). We ran Gruffi on the precomputed R data object obtained from the authors and marked stressed cells (Fig EV4A and B). This dataset is particularly well suited to demonstrate the versatility of Gruffi, as it contained secretory choroid plexus cells, which are undergoing high physiological ER‐stress due to secretion. We therefore derived a choroid plexus score as an additional negative filter score. As no GO‐term exists for choroid plexus, we turned to the recent publication of choroid plexus organoids (Pellegrini *et al*, 2020) identified marker genes, and derived the choroid plexus score (Materials and Methods). Gruffi labeled 74% of the “Undefined” cluster as stressed and an average of 1% of cells from other clusters (Dataset EV3). To ensure proper stress identification, we then performed DGEA on stressed vs. non‐stressed cell and GO‐term enrichment on all enriched coding genes (Materials and Methods). Visualization of enrichments on the protein interaction network using STRING (Fig EV4C) showed that apoptosis, stress, unfolded protein response, and hypoxia dominate cells identified as stressed in this dataset as well.

In the organoid field, a considerable effort has gone into improving culture conditions. This way complex problems could be studied, like the relationship of vascular and brain cells using induced endothelial cells (Cakir *et al*, 2019), or cortical layering (Qian *et al*, 2020) and axon projection (Giandomenico *et al*, 2019) in organoid slice culture. To test whether these methodologies reduced stressed cells in organoids, we applied Gruffi to the published datasets (Appendix Fig S6). Non‐telencephalic cell types such as endothelial cells in (Cakir *et al*, 2019) were not classified as stressed (Appendix Fig S6A–C). Despite these improved culture conditions, stressed cells were present in all three datasets. This was confirmed by the activity of the relevant pathways, key stress genes, and independent PROGENy analysis, suggesting that stress remains a prevalent problem of *in vitro* culture (Appendix Fig S6A–J). Notably, stressed cells made up similar fractions of the datasets as for organoids generated with standard protocols (Appendix Fig S6K and L). These data suggest that while improved methods are useful for investigating processes that are difficult to study in classical organoid preparations, they nevertheless contain stress‐identity cells.

#### Organoids show proper cell‐type specification along the excitatory lineage

**Figure EV5:**
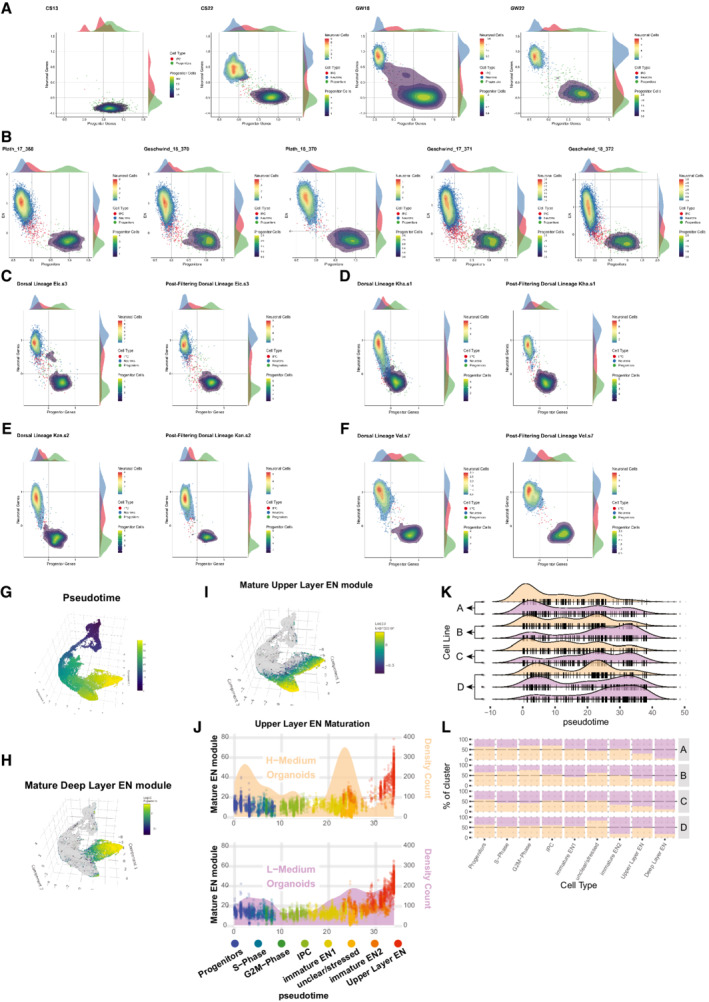
Proper specification and maturation in non‐stressed cells A
Progenitor (*x*‐axis) and excitatory neuron scores (*y*‐axis) calculated in fetal brain datasets across brain development (Materials and Methods, Bhaduri *et al*, 2020). Early datasets from Carnegie stage 13 (CS13) are enriched in progenitors, while late datasets from gestational week (GW) 18 and 22 show both neurons and progenitors.B
Progenitor (*x*‐axis) and excitatory neuron scores (*y*‐axis) applied to multiple datasets of mid‐gestation (Fig 4F) show reliable separation of neurons and progenitors, while only intermediate progenitors (IPCs, red) cluster in between the two populations.C–F
Examples of pre‐ and post‐filtering plots for subtype specification of individual datasets analyzed in this study. After filtering out stressed cells only IPCs remain in between neurons and progenitors.G
3D UMAP of dorsal lineage also shown in Fig 4J colored for pseudotime.H
Expression of the deep layer excitatory neuron (DL‐EN) gene module (Table EV5) is specifically enriched in the DL‐EN cluster (Cluster 9 in Fig 4J).I
Expression of the upper layer excitatory neuron (UL‐EN) gene module (Table EV5) is specifically enriched in the UL‐EN cluster (Cluster 8 in Fig 4J).J
Maturation of UL‐EN in two different media formulations analogous to DL‐EN maturation in Fig 4L. Cells are color coded for clusters (Fig 4J) and plotted along pseudotime (*x*‐axis). The density‐count of cells along pseudotime is shown behind the dots (yellow area for H‐medium, purple area for L‐medium, right *y*‐axis). The position of the points reflects the expression of a module of co‐regulated genes enriched in UL‐EN (left *y*‐axis, Fig EV5I). While proper maturation indicated by expression of the UL‐EN module occurs in both media conditions, in L‐medium maturation is much more frequent.K
Distribution of cells (black lines) and densities (areas) of individual organoids across pseudotime. Organoids derived from the same cell line (A to D) grown in the different media conditions (yellow for H‐medium, purple for L‐medium) are shown on top of each other.L
Cluster contributions per individual organoids (as shown in Fig EV5K). The increased maturation in L‐medium organoids is also reflected by higher proportions of mature cell types (UL‐ and DL‐EN) in L‐medium organoids. Cell numbers were downsampled to account for different library sizes.

A previous study reported that stress in organoids not only leads to impaired cell‐type fidelity but also leads to incomplete maturation (Bhaduri *et al*, 2020). To investigate whether stressed‐identity cells affect specification along the excitatory lineage, we compared progenitor and neuron signatures (Materials and Methods, Dataset EV4). This led to the expected bimodal separation of the fetal samples (Fig EV5A). As this dataset was used to derive the signatures, we confirmed the separation of neurons vs progenitors using an independent fetal dataset with multiple samples around mid‐gestation (Figs 4F and EV5B). Next, we asked how organoid samples separate using those signatures. We calculated signature scores on Bhaduri *et al* organoid datasets and reproduced the previously reported lack of specification (Fig 4G). In contrast, when testing the other datasets analyzed in this study, we detected a fetal‐like specification (Figs 4H and EV5C–F), suggesting proper specification in most organoid datasets.

Nevertheless, organoid cells still separated less than fetal cells, and more cells were scoring low on both progenitor and neural axes. As stressed cells are characterized by the lack of both glial and neural signatures (Fig 2A), we hypothesized that stressed cells may populate the area between neurons and progenitors. After annotating stressed cells, we found two populations in between progenitors and neurons: stressed cells and intermediate progenitors, or IPCs. After stress removal, most of those remaining cells are IPCs (Fig 4I, in red), which are indeed a transitory stage between glia and neurons. Altogether, we find no evidence for general misspecification in organoids. Instead, progenitors and excitatory neurons properly separate, while two specific populations, IPCs, and stressed cells, lack specific neuronal or progenitor signatures.

#### The presence of stressed cells does not affect the maturation of other cell types

Besides a lack of cell‐type specification, incomplete maturation due to stress was previously suggested (Bhaduri *et al*, 2020). As a positive control for maturation, we took two media conditions that affected maturation (Eichmüller *et al*, 2022). While the original organoid medium contains high concentrations of nutrients and amino acids and thus supports progenitor expansion, it is less suitable for neuronal maturation. A low‐nutrient media composition, in contrast, based on a defined 2D neuron culture medium (Bardy *et al*, 2015) more closely resembles the cortical environment and enabled further maturation of neurons.

To test whether stress affects maturation, we grew pairwise matched samples in the two different media conditions and analyzed them together (Fig 4J). We calculated the pseudo‐temporal trajectory of the excitatory lineage and graded each dataset for maturation along this trajectory (Fig EV5G, Materials and Methods). Our results showed that the fraction of stressed cells does not explain maturation differences (Fig 4K), but the choice of media does: low‐nutrient media improved neural maturation. To understand the impact on maturation on single‐cell level, we plotted individual cells along the maturation trajectory, split by media condition (Fig 4L). In addition, to assess expression changes associated with mature states, we generate scores for mature neurons (Figs 4L and EV5H–J, Dataset EV5). This revealed that while organoids grown in either condition had abundant excitatory neurons, those in high‐nutrient media remained mostly immature, while organoids grown in low‐nutrient media contained more mature neurons (Figs 4L and EV5J–L). In sum, the presence and abundance of stressed cells in a sample have negligible effects on neural maturation, while measurable differences arise by the choice of media.

#### Gruffi can be applied to other organoid systems

**Figure EV6:**
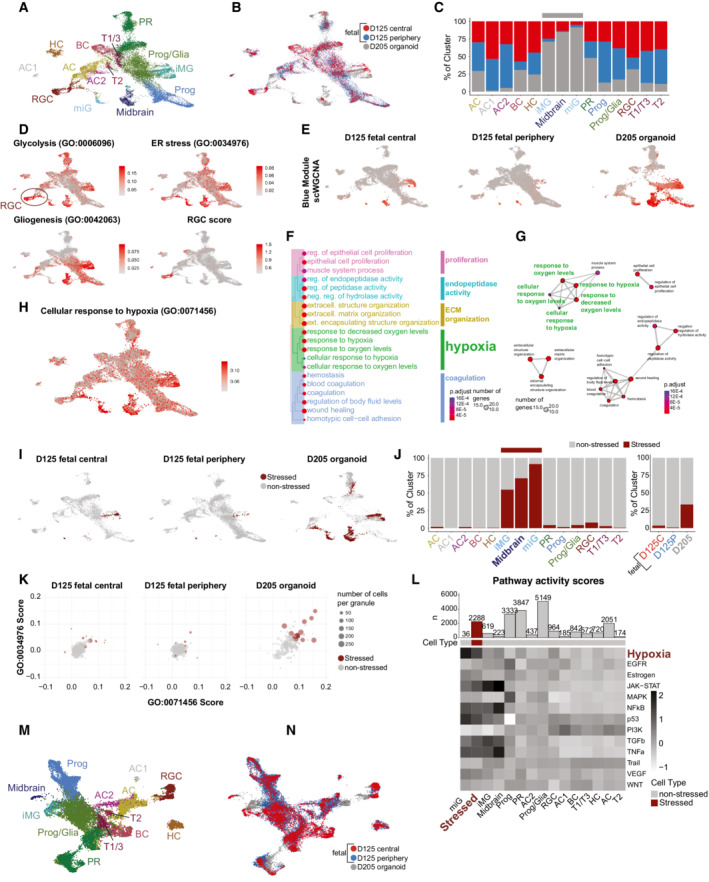
Gruffi identifies stressed cells in retinal organoids A
UMAP showing CCA integrated and re‐clustered datasets as in analysis of fig 6 in Sridhar *et al* (2020) and colored based on annotation provided by the authors.B
UMAP color coded for datasets: two fetal (125 days central and periphery) and one organoid dataset (205 days).C
Bar plot with downsampled contribution per cluster. There are three clusters mostly consisting of retinal organoid cells.D
GO‐term scores as used previously in Gruffi. Note that glycolysis is very high in RGCs, a cell type known to be very metabolically active. Additionally, a score for RGCs is shown consisting of the marker genes: *ISL1*, *SNCG*, *RBPMS*.E
scWGCNA analysis on retinal data identifies one module (blue module) that is enriched in organoid cells.F, G
GSEA of blue module from scWGCNA reveals enrichment of hypoxia related terms in this module.H
The GO‐term “cellular response to hypoxia” (GO:0071456) marks specific populations in the dataset and will be used in the stress annotation.I
Gruffi stress identification using response to hypoxia and to ER stress as selecting terms, as well as gliogenesis and the RGC score as negative filters. Color code shows stressed cells and UMAP depicts each different dataset.J
The three clusters that were biased toward organoid cells in (C) show strong enrichment in stressed cells, whereas other clusters have generally low percentages (left). Similarly, the organoid dataset shows about 33% of stressed cells, while in fetal there are almost none (right).K
Per granule score of GO‐terms with response to hypoxia on the *x*‐axis and response to ER stress on the *y* axis shows that stress scores are generally low in fetal but increased in organoids. The size depicts the number of cells that are within the respective granule.L
PROGENy pathway activity score for the clusters as shown in L reveals that Hypoxia is uniquely enriched in stressed cells. Note that miG is a remnant of prior cell‐type annotation and contains just 36 cells. It is therefore to be regarded as an unrepresentative leftover population.M, N
After stress filtering the datasets are re integrated and all downstream visualizations are re‐computed.

In our analyses, we focused on brain organoids as they are among the most diverse and largest organoid systems. However, to test whether Gruffi can be used in the context of other systems, we applied it to a recent publication of retinal organoids (Sridhar *et al*, 2020). To this end, we used mature (205‐day‐olds) retinal organoids as well as two fetal reference datasets (Fig EV6A–C). To consider the different stressors and metabolic pathways of the retina, we optimized the stress scoring by including a different stress selecting score instead of glycolysis (“cellular response to hypoxia”), as well as an additional negative filter (RGC score, Materials and Methods, Fig EV6D–H). Gruffi identified abundant stressed cells in the retinal organoids, whereas they were rare in fetal retina samples. Notably, stressed cells were mainly localized to clusters that were strongly biased toward organoid cells and showed the hypoxia pathway activation also found in stressed to identity cells in brain organoids (Fig EV6I–N). Thus, the prevalent stressors and the unique metabolic pathways of each system should be considered in the stress score selection. If done so, Gruffi can also be applied to any dataset outside of brain organoids.

## Discussion

Brain organoids generate complex, structured tissue *in vitro* (Eiraku *et al*, 2008; Kadoshima *et al*, 2013; Lancaster *et al*, 2013; Paşca *et al*, 2015; Qian *et al*, 2016). Besides their tremendous potential for modeling human diseases (Sidhaye & Knoblich, 2021), it is critical to understand and account for their limitations. Here, we showed that a population of stressed cells exists in all analyzed organoid samples, as a biologically distinct population, which is not found *in vivo*. We provide an in‐depth analysis of these cells that hopefully will help to decipher the needs of 3D tissue in culture.

While an experimental solution is the end goal, stress is present in published and current experiments. We found that neither endothelial co‐culture, nor slice‐culture eliminated stressed cells, underlining that experimental solutions are challenging. For example, for media to flow through vasculature, the vascular system must be free of any closure, and the diameter of vessels must either permit flow without direct pumping or requires such. For slice culture, continued cell proliferation leads to the thickening of the tissue and might be recreating the original problem. This could be addressed by re‐slicing as in (Qian *et al*, 2020), but either other stressors, or the persistence of stress‐identity cells leads to a detectable population of ER‐stressed‐ and hypoxic‐cells.

To tackle this issue, we developed Gruffi, an *in silico* tool to bioinformatically identify and remove these cells, based on stress pathway activity scoring. Gruffi comes with a graphical and interactive interface. It integrates into a typical single‐cell analysis workflow using Seurat but can be used in other pipelines as well. The resulting stress‐decontaminated samples displayed a clearer representation of the fetal neural development and showed higher similarity to *in vivo* samples. Even if future organoid protocols may resolve cellular stress, earlier published data still suffer from stress, which negatively impacts data integration. Gruffi, however, can recover such data for comparison, reducing the need for performing new experiments.

We observed diverse stress pathway activity, and it is important to understand how they are connected on a cell biological level. Our results are compatible with earlier observations that the organoid core, but not surface, is hypoxic (Qian *et al*, 2019), explaining why stress characterizes only in a defined set of cells. This is further supported by a recent preprint employing spatial transcriptomics to identify hypoxic metabolic programs in the organoid core (Uzqiano *et al*, 2022).

The central role of hypoxia can explain the other transcriptional changes. The lack of oxygen triggers a metabolic shift, from oxidative phosphorylation to anaerobic glycolysis. Hypoxia also triggers ER stress, in two ways. First, glycolysis is much less efficient in energy production, leading to energy depletion, and consequently a stronger metabolite transport is needed. These transporters are secreted via the ER‐pathway (Loike *et al*, 1992), triggering the unfolded protein response (UPR; Lee *et al*, 2020). At the same time, the depletion of energy leads to a pH imbalance, affecting organelles that rely on ATP‐dependent transporters for ion homeostasis (Chiche *et al*, 2010).

Our results are consistent with a previous observation that acute hypoxia in cortical spheroids triggers a strong ER‐stress response (Pașca *et al*, 2019). However, a simplistic, one‐dimensional distance‐to‐surface model of nutrient availability cannot explain the heterogeneity of stress marker expression. It is an interesting future direction to determine different cellular niches, for example, by local variation in oxygen and nutrient levels. Similarly, an interesting question for future studies is how cellular heterogeneity leads to the differential expression of stress markers in close neighboring cells.

Importantly, stress identification is just the first application and proof of principle for granular functional filtering. This flexible framework can be extended to many other applications in single‐cell analysis. As long as cells form an identity, so that they group together in any low‐dimensional representation and co‐express a defined gene set (GO‐terms, KEGG‐pathways, etc.), the cells can be identified, studied and removed. Here, we applied Gruffi to remove stressed cells from brain organoid datasets, but we think that there are many other applications possible, such as selecting cells from a lineage or cells responding to a treatment. Currently, brain organoids are the largest and longest‐cultured 3D organoid systems and are therefore particularly affected by stress. As 3D tissue models and investigations become ever more sophisticated, cell culture‐induced artifacts are more important to account for. Therefore, we expect that our approach will find many applications beyond its original scope.

## Materials and Methods

### Experiments

#### Stem cell culture

We obtained the “HPSI0114i‐rozh_5” (female) line from the HipSci catalog (Streeter *et al*, 2017), hiPSC cells were cultured following the HipSci guidelines. We also grew organoids from the feeder‐free human ES cell line (H9; WA09 from WiCell, Female). The two iPSC Lines SCCF – 177 (177J clone#8, female) and SCCF – 178 (178J clone#5, male) were generated at the IMBA Stem Cell Core Facility and are part of the IPSC Biobank. The study was approved by the local ethics committee of the Medical University of Vienna (MUV). After informed consent, a skin biopsy was taken from three healthy donors, and fibroblasts were isolated for iPSC reprogramming. iPSC lines were generated using the Sendai virus (CytoTuneTM‐iPS 2.0 Sendai Reprogramming Kit, Thermo Fisher Scientific) carrying the Yamanaka reprogramming factors OCT3/4, SOX2, c‐MYC and KLF4 factors. All cell lines were used within 10 passages from last STR profiling and tested regularly for mycoplasma contamination. We additionally used the above cell lines (177 and 178) for the media comparison experiments (Fig 4). The cell lines were evaluated and cultured as the HipSci lines. Briefly, cells were seeded on vitronectin (Stemcell Technologies, cat#07180) coated plates and fed every day with E8 essential media. Cells were passaged as single cells using Accutase (Sigma) with Revitacell cell supplement (1/100, Invitrogen, cat#A2644501), and grew until 70% confluency, then we replated. Cultured cell lines were routinely tested for mycoplasma contamination by PCR (Janetzko *et al*, 2014).

#### Organoid culture

Organoids were generated as described in Esk *et al* (2020). Briefly, 150 μl/well of Essential 8 media supplemented with RevitaCell (1/100) containing the corresponding cell suspension for 9,000 cells/well were plated for each cell line using low attachment 96‐well plates (Sigma CLS7007). Briefly, the protocol entailed the following steps: On day 3, media was replaced to Essential 8 media and from day 6 on, embryoid bodies were transferred to neural induction media (NI) and 200 μl/well was exchanged every day. On day 10, when embryoid bodies are about 500–600 μm in thickness and neuroepithelium is evident, the aggregates were transferred to 10‐cm dishes and embedded in matrigel (MG) droplets. On day 13 and 14, the media was changed to Improved Differentiation Media without ascorbic acid (Imp‐A) containing 3 μM CHIR. After that, the media was replaced every 3–4 days. On day 19, the dishes were transferred to an orbital shaker. On day 25, the organoids were fed with Improved Differentiation media with ascorbic acid (Imp+A) and the media was replaced every 3–4 days. On day 40, the two different culture methods (Brainphys & Imp+A) diverged. The “Imp+A” organoids were further cultured in Imp+A supplemented with 1%MG, BDNF (20 ng/ml), GDNF (20 ng/ml) db‐cAMP (1 mM). The “Brainphys” (BP) organoids were gradually transitioned to BP media in three feeding steps: 75–25%: 50–50%, 25–75% (Imp+A & BP). From that point, they were cultured in BP supplemented with 1%MG, BDNF (20 ng/ml), GDNF (20 ng/ml) db‐cAMP (1 mM).

#### Media composition

NI media: Neural Induction medium consisting of DMEM/F12 (Thermo Fisher Scientific) with 1% N2 Supplement (Thermo Fisher Scientific), 1% MEM‐NEAA (Sigma Aldrich), 1% Glutamax (Thermo Fisher Scientific) and 1 μg/ml Heparin. Imp‐A: of 50% DMEM/F12 (Thermo Fisher Scientific), 50% Neurobasal (Thermo Fisher Scientific), 0.5% N2 Supplement (Thermo Fisher Scientific), 2% B27—Vitamin A (Thermo Fisher Scientific), 1% Glutamax (Thermo Fisher Scientific), 0.5% MEM‐NEAA (Sigma Aldrich), 50 μM 2‐ME solution, 1% Penicillin/Streptomycin (Sigma Aldrich) and 0.025% Insulin solution (Sigma Aldrich). Imp + A (HN): Imp‐A with 2.5 mM Ascorbic Acid, 2 g/l Bicarbonate (Sigma Aldrich). BP (LN): BrainPhys Neuronal Medium (Stem Cell Technologies), 2% B27 + A (50×, Thermo Fisher Scientific), 1% N2 supplement (Thermo Fisher Scientific), 200 nM Ascorbic Acid (Sigma Aldrich), 0.2% CD Lipid Concentrate (Thermo Fisher Scientific), 7.4% glucose, and 1% Penicillin/Streptomycin.

#### Single‐cell sequencing

Organoids were cultured to 120 days, then washed in PBS and dissociated using the gentleMACS Dissociator (Miltenyi Biotec) in program NTDK1 using the enzyme mix: Trypsin (Sigma Aldrich)/Accutase (Sigma Aldrich; 1:1) containing 10 U/ml DNaseI (Thermo Fisher Scientific). The washed cell suspension was passed through a 70 μm cell strainer.

In the newly generated datasets, we pooled cells from samples from four different genotypes and were combined in a lane (other cell lines used for other purposes). We additionally used sample barcoding using lipid‐anchor barcoding following instructions as in (McGinnis *et al*, 2019) with reagents kindly provided by the authors, but we relied on SNP‐based cell line demultiplexing as described in (Kang *et al*, 2018; described in the following section) and sample barcoding was not used.

Cells were counted and the suspension was loaded onto a Chromium Single Cell 3′ B Chip (10× Genomics, PN‐1000073) and processed through the Chromium controller to generate single‐cell GEMs (Gel Beads in Emulsion). scRNA‐seq libraries were prepared with the Chromium Single Cell 3′ Library & Gel Bead Kit v.3 (10× Genomics, PN‐1000075). Ready 10× libraries were sequenced paired end (R1:28, R2: 89 cycles) on NovaSeq (Illumina).

### Data analysis

#### Public datasets

We used the following public datasets for this study: dbGaP Study Accession: phs001836.v1.p1. (de la Torre‐Ubieta *et al*, 2018; Polioudakis *et al*, 2019); ENA PRJEB33917 (Kanton *et al*, 2019); GEO GSE132672 (Bhaduri *et al*, 2020); EGA EGAD00001006332 (Eichmüller *et al*, 2022); GEO GSE129519 (Velasco *et al*, 2019); GEO GSE124174 (Giandomenico *et al*, 2019); GEO GSE134049 (Cakir *et al*, 2019); GEO GSE137941 (Qian *et al*, 2020); from UCSC Cell Browser (Eze *et al*, 2021); GEO GSE142526 (Sridhar *et al*, 2020).

#### Cell line demultiplexing

For pooled 10× GEX libraries, the donor cell‐line of the assayed cells was determined by genotype‐based demultiplexing using souporcell (Heaton *et al*, 2020). The pipeline was run with default settings, providing all donor genotypes through the known_genotypes parameter, and providing the cellranger bam, the cellranger filtered barcodes file, and the reference fasta as input. Donor genotype vcfs were pre‐generated using HaplotypeCaller from the Genome Analysis Toolkit (GATK) v4.1.2.0 on bwa mem 0.7.17 aligned WGS reads following the nf‐core/sarek v2.5.1 pipeline. WGS reads were obtained from respective sources: (i) SRA database for H9/SRR6377128, (ii) from the ENA database for the HIPSCI line rozh_5/ERR1871976 or (iii) WGS data generated by the IMBA stem‐cell facility for SCCF—177 (177J clone#8, female) and SCCF—178 (178J clone#5, male).

#### Single‐cell analysis

We first aligned reads to the GRCh38 human reference genome with Cell Ranger 3.1 (10× Genomics) using pre‐mRNA gene models and default parameters to produce the cell‐by‐gene, unique molecular identifier (UMI) count matrix. UMI counts were then analyzed in R, using the Seurat v4. We filtered for high‐quality cells based on the number of genes detected (> 500). Thereafter, expression matrices of high‐quality cells were normalized (“LogNormalize”) and scaled to a total expression of 10 K UMI for each cell. Regression of variables at this step did not improve clustering results; hence, no variables were regressed or removed.

#### Non‐telencephalic cell exclusion

Before integration datasets were checked for quality, as certain IPS lines are prone to mis‐differentiation. As a consequence, multiple datasets included non‐telencephalic cells that would interfere with the CCA integration; henceforth, we applied initial filtering for CNS cells (Appendix Fig S5). To exclude mis‐differentiated cells, we used a recently published fetal organ atlas (Cao *et al*, 2020). Processed data were downloaded, and cell‐type annotation was modified to reflect major cell types for a basic classification. All CNS cell types were grouped together under one annotation to determine properly specified clusters. Next, an xgboost classifier was trained to distinguish major cell types on the RNA assay data using the top variable genes of the fetal dataset with parameters determined by cross‐validation. This classifier was applied to each dataset: (i) Datasets were preprocessed individually and clustered in UMAP space; (ii) The expression of the RNA assay was used to classify each cell according to the cell groups of the training dataset; (iii) Classification was summarized per cluster; and (iv) all clusters that were not classified as CNS cells and were clustering separate of neuronal lineages were filtered out. The cleaned datasets were used for CCA integration.

To establish the maximal mitochondrial‐, and ribosomal RNA fractions, we plotted these against feature counts and each other, and set thresholds to remove extreme outliers. A group of cells showed a distinctly high ribosomal fraction (> 30%). We found that these cells correspond to one cluster coming from one dataset (Velasco organoid 21, Dataset EV1) and are non‐neural in gene expression. We used the same threshold value for maximal mitochondrial read fraction for simplicity.

#### Downstream analysis

Variable genes were identified by Seurat's FindVariableFeatures implementation (“FastLogVMR”). Next, we aligned and merged sequencing libraries by Seurat's canonical correlation analysis or CCA (dimensions: 50; Butler *et al*, 2018) using the intersection variable genes across datasets.

Next, principal components were calculated on the variable genes, and the first 50 components were then used to calculate UMAP coordinates. For clustering, we used Seurat's implementation of snn/Louvain clustering. Therein, we first calculate the k‐nearest‐neighbor (knn) graph of cells in PCA‐space (dimensions:50). Based on Jaccard similarity scores on the knn graph, the shared nearest neighbor (snn) graph is computed. Louvain clustering on the snn graph identified clusters of cells. Differentially expressed genes were identified by Wilcoxon‐test, and filtered for *P*‐values below 0.001, and fold change larger than 2.

We found a group of 1,304 interneurons that formed a separate cluster on the very top of the UMAP (Fig 1). These constituted 7.61% of all interneurons and were 94% originating from the Kanton S3 dataset. Both interneuron clusters showed similar expression of classic interneuron markers, and pairwise differential gene expression analysis showed no meaningful differences. Therefore, we lumped these two clusters together.

#### Differential gene expression analysis (DGEA)

We used the Wilcoxon test via the FindAllMarkers() and FindMarkers() to identify differentially expressed genes, with the folllowing parameters: return.thresh = 0.001; min.pct = 0.1; min.diff.pct = 0.01; min.cells.group = 100; min.cells.feature = 100; logfc.threshold = 0.25.

#### GO‐term enrichment

GO‐term enrichment was performed on coding, differentially expressed genes, using the STRING toolkit (Szklarczyk *et al*, 2019). Enriched terms were filtered by false discovery rate (FDR), so that the Benjamini–Hochberg multiple testing corrected *P*‐values < 0.05. Next, terms were ranked by Enrichment Strength, that is the Log10(observed/expected) codung gene count. Where “expected” is the number of proteins with a given annotation that is expected in a random gene set of the same size.

#### Integration of organoid and fetal data

We obtained raw data for fetal cortical single‐cell datasets covering age comparable to organoid datasets (de la Torre‐Ubieta *et al*, 2018; Polioudakis *et al*, 2019), preprocessed and analyzed it, the same way as we did for the organoids. The individual sequencing lanes were merged per fetal datasets and integrated with Seurat, as before. The integrated organoid dataset was uniformly downsampled to 24,211 cells, to match the total sample size of the fetal datasets. The resulting individual (original) datasets were then reference‐integrated to the fetal dataset as follows: First, 3,000 integration anchors were computed with Seurat's SelectIntegrationFeatures() and FindIntegrationAnchors() functions, where the fetal datasets were defined as reference. By default, the integration by IntegrateData() was performed using CCA, setting parameter k.weight to 50. Further steps, such as the determination of variable features, scaling, the computation of PCA and UMAP embeddings and the SNN Graph were performed as for the organoid integration.

#### Effect of different basic QC filtering parameters

The integrated dataset containing all cells above 500 UMIs was analyzed with different cutoffs for basic metrics that are often used in QC: UMI count, fraction of mitochondrial, and fraction of ribosomal reads (Appendix Fig S3). Different UMI count cutoffs were applied to the integrated dataset, and the fraction of cells lost in each cluster of (Fig 1A) was quantified. Next, the fraction of UMIs from the mitochondrial DNA was quantified per cell (MT* genes), and cells were binned into very‐high (> 20%), high (20% > *x* > 10%), and normal bins. Cells corresponding to each class were visualized on the umap and contrasted to stressed cells. The same analysis was repeated for ribosomal transcripts (RPL* and RPS* genes). Cells with very‐high (> 30%) ribosomal content was visualized on the UMAP of the unfiltered integrated object. This cutoff was used to generate (Fig 1A). Cells with high (*x* > 20%) ribosomal content was visualized on the UMAP of (Fig 1A) and the fraction of cells lost per cell type at this threshold was quantified.

#### Analysis of early radial glia

The (Eze *et al*, 2021) dataset was downloaded the UCSC Cell Browser, filtered for cells with a gene count higher than 500 and less than 15% mitochondrial reads and analyzed using Seurat as described above including log‐normalization, scaling, the computation of the 2000 most variable genes and PCA computation (Fig EV2). The UMAP coordinates were used as provided by the authors. The detected genes for the GO‐terms of interest were compared with the organoid integration dataset to ensure similarity. Gruffi stress scoring was applied. To compare granule‐wise scores in the fetal and organoid dataset, the scores for each of the relevant GO‐terms were aggregated per granule and colored for Gruffi stress annotation as shown in Fig EV2E.

#### Individual analysis of Velasco 7 dataset

The dataset was filtered for high‐quality cells with a higher gene count than 1,000 and analyzed using Seurat as described above including log‐normalization, scaling, the computation of the 2,000 most variable genes, PCA and UMAP embedding computation (Fig EV3).

#### Effect of integration with non‐telencephalic cells

The Velasco 5 dataset was plotted individually without non‐telencephalic cell exclusion, and cells were colored based on non‐telencephalic cell classifier (Appendix Fig S5). A cluster‐wise correlation to the (Cao *et al*, 2020) reference data was performed to support cell‐type annotation. To represent distance of different cell types in a linear way, we used PCA space (Appendix Fig S5 and Fig 4E). To account for different weights of PCs, each PC was multiplied by the standard deviation it explained. This weighted Euclidean distance was presented as a heatmap using pheatmap 1.0.12, or as a graph embedding visualizing similarity with edge strengths (Epskamp *et al*, 2012). Differences in GO‐terms among variable features were visualized using revigo (Supek *et al*, 2011) and clusterProfiler 4.2.2 and enrichplot 1.14.2 (Wu *et al*, 2021). GO terms were calculated comparing both datasets in clusterProfiler, and only those that were unique to each condition were plotted with enrichplot.

#### Effect of different culture conditions on stressed cells

Datasets for: (Cakir *et al*, 2019), (Giandomenico *et al*, 2019), and (Qian *et al*, 2020) were downloaded from GEO (see “Public datasets” section) and filtered for cells with more than 500 genes and < 15% mitochondrial reads (Appendix Fig S6). Processing was performed in Seurat and Gruffi was applied as for other analyses. For the Qian *et al*, dataset the target granule size was set to lower.median = 75 and upper.median = 150 to optimize granule size in this smaller dataset. PROGENy pathway scoring and visualization was performed as for other analyses.

#### Application of Gruffi to retinal dataset

Datasets from (Sridhar *et al*, 2020) were downloaded from GEO (see “Public datasets” section), and cell‐wise metadata annotation was kindly provided by the authors (Fig EV6). Processing was performed in Seurat, and Gruffi was applied. As RGCs showed strong glycolysis in fetal datasets, a phenomenon already described (Liu & Prokosch, 2021), we decided this score was not suitable for filtering. We selected genes that are specific for RGCs as an additional score (*ISL1*, *SNCG*, *RBPMS*; Langer *et al*, 2018). After applying scWGCNA as done previously, we selected “cellular response to hypoxia” as new second stress score. Thus, Gruffi was used with response to hypoxia and response to ER‐stress as positive, and with gliogenesis and the RGC score as negative selectors. All other analyses were performed as detailed for other Figures.

### The Gruffi package

The Gruffi package contains all functions for the identification, inspection, and filtering of stressed cells using command line or graphical user interface (Shiny app). Gruffi functions encompass the following major steps as in (Fig 2B): (1–3) Accession of GO‐term gene sets and single‐cell stress scoring; (I–III) Data partition into granules and small‐granule reclassification; (4) Aggregate score calculation per ensemble; (5) Automatic estimation of stress threshold, with possible manual adjustment and inspection; (6) Stressed cell assignment and filtering.

#### Single‐cell scoring

We defined specific GO‐terms relevant for functional processes in stress and differentiation. Gene lists for each GO‐term were downloaded from Ensembl via BiomaRt (Durinck *et al*, 2009) and intersected with detected genes. Alternative database access is also implemented (see R‐package documentation). We then generalized a widely used cell‐cycle scoring method based on aggregated gene set activity (Tirosh *et al*, 2016) and used its implementation in Seurat (*AddModuleScore*). Briefly, in the *AddModuleScore* function, the following steps have been implemented as in the original: (i) Take a target set of genes; (ii) Calculate their average expression; and (iii) Create control sets of genes. The control gene‐sets are used to control for the cell‐to‐cell variability in quality and depth. To create the control sets, first all genes are binned by expression levels (25 bins), then for each gene in the target set, randomly select 100 genes from its expression bin, and finally (iv) subtract the average of control from each target gene. The expression binning is important, because expression levels affect the variability of gene expression (Tirosh *et al*, 2016). Gene lists of “glycolytic process” (GO:0006096) and “response to endoplasmic reticulum stress” (GO:0034976) were downloaded and intersected with detected genes, then used to evaluate stress state.

#### Data partitioning and reclassification

Gene detection in single cells is noisy. To overcome this noise, we grouped cells into small clusters (that we call granules) by high‐resolution snn‐clustering (as in the manuscript, using the algorithm of Seurat). Gruffi's *aut.res.clustering()* performs a simple parameter search (for *clustering‐resolution*) that results in a median of 100–200 cells per granule, which we call the optimal resolution, as clusters contain a statistically robust number of cells. All these parameters are also manually customizable. Next, clustering is performed at the determined resolution, resulting in cells separated into 100's of granules, depending on the size of the dataset. Finally, all cells in granules with < 30 cells are reassigned to the nearest cluster center in the 3D UMAP space (Euclidean distance) using *reassign.small.clusters()*.

#### Thresholding and stress annotation

Finally, the average GO‐scores for each granule were calculated, and stress level per granule was evaluated. We propose two possible methods to estimate an upper threshold for the assignment of stressed granules for one GO term. (a) Determining manually, based on the expression of stress genes and stress score values on UMAP. Based on these, an empirical quantile (90% if observing 10% stressed cells) as threshold can be assigned. (b) Automatic stress threshold estimation by *Shiny.GO.thresh()*. For this, we refer to cell number normalized, mean GO scores per granule. In the following this will be referred to as granule score. We assume that: (b1) The granules consist of a statistically sufficient number of cells. (b2) GO scores of non‐stressed cells independently follow the same unknown distribution. (b3) GO scores of stressed cells are significantly higher than GO scores of non‐stressed cells. (b4) The dataset consists of more non‐stressed than stressed cells. Assumptions (b1) and (b2) together with cell number normalization allow us to use the central limit theorem and hence fit a normal distribution to the granule scores of non‐stressed granules. Based on (b3) and (b4) we conclude that respective non‐stressed GO‐scores are small, and the mean of the normal distribution can be estimated by the median of granule scores. The standard variation of the normal distribution is now estimated only w.r.t. to GO scores smaller than the median of granule scores. Now, the theoretical 99% quantile of the fitted normal distribution can be computed and used as an upper threshold.

Assumption (b1) is fulfilled by the automatic clustering resolution search and the reassignment of cell granules with less than 30 cells. Although we based our analysis on thresholds retrieved by this method, as we cannot assure that assumptions (b2) to (b4) hold true, we highly recommend further inspections and refinements of suggested thresholds in any case. To do so, we propose to visually monitor further manual adjustments via the implemented Shiny App interface.

When considering a combination of GO terms, for example, response to endoplasmic reticulum stress and glycolytic process, we combine the respective thresholds such that a cell is assigned as stressed if either upper threshold is crossed. In case one additionally wants to include a GO term for non‐stressed cells, for example, gliogenesis, the above threshold method can be applied, too, but in this case, granules with a score higher than the threshold are assigned as non‐stressed. Finally, based on the thresholds on each score, stressed cells are annotated and can be excluded from the dataset.

### Other analyses

#### Protein–protein interaction maps

We selected all genes enriched in either stress clusters, and jointly ranked them by descending log2FC. We selected the top 150 coding genes, and visualized the “high‐confidence” connected component of the protein–protein interaction network using the STRING database (v11.5; Szklarczyk *et al*, 2019), links denoting the confidence of connection (permalink: bxso1NJafq8R).

#### Pathway visualization using ShinyGO and KEGG

The top 150 coding genes (as above) were provided for ShinyGO v0.741 (Ge *et al*, 2020) with default parameters and the background gene list of all 26,439 detected genes (from the RNA assay). ShinyGO's visualized enriched KEGG pathways using Pathview and relevant pathways were selected.

#### Comparison of granular and single‐cell scoring

For granular scoring, we used the annotation and approach from (Fig 2). For single‐cell scoring, we used the exact same approach, but skipped the granule average calculation of stress scores, and instead, we calculated the stress threshold on single‐cell scores. For fair comparison, we adjusted the quantile cutoff parameter in the single‐cell scoring, so that it results in a similar number of stressed cells, as in the granular approach. We then took the symmetric difference of these to find cells only flagged by either, but not both methods. Group median values for were plotted for the four categories (Both, gSC, scSC, Non‐stressed).

#### The separation of stressed neurons

Stressed cells clearly separated into two major groups, as also seen in (Fig 1). Therefore, we separated Gruffi's classification into two categories. Low‐resolution clustering (res.0.1.ordered) separated glia (cl.1–2) from neurons (remaining clusters) both better (less clustering artifacts) and simpler than higher resolutions (0.3, 0.5). Intersecting this binary annotation with Gruffi's stress annotation (T, F), separated cells into the four clusters: Neurons, Stressed Neurons, Stressed Progenitors, Progenitors, visible in (Appendix Fig S4B).

#### Progeny pathway activity scoring

Progeny pathways scoring was performed as in vignette, with the following parameters top 200 genes. To visualize the differences between stressed cells identified by typical clustering (Fig 1B) and Gruffi (Fig 2F), we separated “Stressed Neurons” and “Stressed Prog.” clusters into subsets identified, or not identified by Gruffi, yielding four groups: “Stressed Prog. (Clustering),” “Stressed Prog. (Gruffi),” “Stressed Neurons (Clustering),” “Stressed Neurons (Gruffi)” (Appendix Fig S4C). As progeny failed to run on the full object, we randomly downsampled the full dataset to 33.3% of the cells (> 50 K cells). We visualized the scores using pheatmap with ward.D2 hierarchical clustering and separated the three most distinct clusters. For (Fig 3B) we displayed all clusters > 2% of all cells (Appendix Fig S4D), and all clusters are displayed in (Appendix Fig S4E).

#### Choroid Plexus scoring and stress identification in Samarasinghe *et al*

We obtained the Seurat R object of (Pellegrini *et al*, 2020) from cells.ucsc.edu and performed DGEA by Wilcoxon test in Seurat. We used the clustering presented in the paper and contrasted “mature choroid plexus” to all other clusters. We calculated a choroid plexus score from the resulting 192 genes (log2fc > 1, *P*.adj < 0.01, pct.expr > 33%, Dataset EV4) and provided this to Gruffi as a negative score (like gliogenesis). We then calculated differential gene expression on stressed cells vs. non‐stressed cells, as identified by Gruffi. The resulting 16 genes (log2fc > 1, *P*.adj < 0.01) were then analyzed in STRINGdb as before (permalink: bwEKXY7CP0p8).

#### Neural and glial identity scores for cell‐type specification

As previously (Bhaduri *et al*, 2020), we grouped all neural or progenitor classes to define the respective signatures in fetal samples, based on DGE analysis. After intersection with genes that are detected in organoid datasets, the top 30 genes were used for EN, or progenitor signatures (Dataset EV5). From these, per‐cell subtype scores were calculated using the AddModuleScore() function of Seurat. XY‐scatter density plots were drawn by plotting a progenitor score and a neuronal score on the *X* and *Y* axis, respectively. The cells were colored based on cell‐type reflecting progenitors, neuronal cells, and intermediate progenitors (IPCs), which would physiologically be an intermediate state.

#### Pseudotime analysis of maturation

For analysis of the “dorsal lineage maturation,” datasets of pairwise H‐ and L‐medium organoids were integrated as outlined above. The datasets were then transferred to monocle3 and UMAP was calculated with three dimensions. The trajectory graph was constructed on the three‐dimensional dataset from progenitors to mature neurons. To compare the maturation of gene expression modules of co‐regulated genes were calculated with the *find_gene_modules()* function. A module for upper layer and deep layer neurons was selected for each mature dataset (Dataset EV5). To plot maturation in the different datasets, each cell was plotted along pseudotime (*x*) versus the expression of the respective score (*y*).

## Author contributions

**Ábel Vértesy:** Conceptualization; data curation; software; formal analysis; supervision; funding acquisition; validation; investigation; visualization; methodology; writing – original draft; project administration; writing – review and editing. **Oliver L Eichmüller:** Conceptualization; resources; data curation; formal analysis; validation; investigation; visualization; methodology; writing – original draft; writing – review and editing. **Julia Naas:** Data curation; software; formal analysis; validation; investigation; visualization; methodology; writing – review and editing. **Maria Novatchkova:** Data curation; formal analysis; validation; investigation; methodology. **Christopher Esk:** Resources; supervision; funding acquisition; investigation. **Meritxell Balmaña:** Resources; investigation. **Sabrina Ladstaetter:** Validation; investigation. **Christoph Bock:** Resources; supervision; funding acquisition; writing – review and editing. Resources; supervision; funding acquisition; writing – review and editing. **Juergen A Knoblich:** Conceptualization; resources; supervision; funding acquisition; project administration; writing – review and editing.

In addition to the CRediT author contributions listed above, the contributions in detail are:

A.V., O.L.E., and J.A.K. conceived, and J.A.K. and A.H. supervised the project. A.V. and J.N. developed the stress identification algorithm. Data analysis was performed by A.V., O.L.E., J.N., and M.N. Organoids were grown by C.E. and M.B.E., and sequencing libraries were prepared by A.V. and S.L. A.V. and O.L.E. wrote the manuscript with input and editing from J.A.K. and J.N. Funding acquisition: J.A.K., A.v.H., C.B., and A.V.

## Disclosure and competing interests statement

J.A.K. is on the supervisory and scientific advisory board of a:head bio AG (aheadbio.com). J.A.K. is an inventor on several patents relating to cerebral organoids. The authors declare that they have no conflict of interest.

## Supporting information




Appendix S1

Expanded View Figures PDF

Dataset EV1

Dataset EV2

Dataset EV3

Dataset EV4

Dataset EV5

## Data Availability

The single‐cell RNA‐sequencing data have been uploaded to gene expression omnibus (GEO) under reference number GSE205554 (http://www.ncbi.nlm.nih.gov/geo/query/acc.cgi?acc=GSE205554). We used publicly available raw sequencing data from the following publications (Cakir *et al*, 2019; Giandomenico *et al*, 2019; Kanton *et al*, 2019; Polioudakis *et al*, 2019; Velasco *et al*, 2019; Bhaduri *et al*, 2020; Qian *et al*, 2020; Sridhar *et al*, 2020; Eze *et al*, 2021; Eichmüller *et al*, 2022) and obtained wild‐type patient data from the authors of complying with ethical and data safety requirements (Khan *et al*, 2020; Samarasinghe *et al*, 2021). The Gruffi package is made available under github.com/jn‐goe/gruffi. The code for analysis will be accessible on Github: github.com/vertesy/Limited.Stress.in.Brain.organoids. The following custom function libraries were used for the analysis: *Stringendo*, *ReadWriter*, *CodeAndRoll2*, *MarkdownHelpers*, *ggExpress*, *Seurat.Utils*, all freely available under github.com/vertesy.
