# Supplementary material for: Gruffi: an algorithm for computational removal of stressed cells from brain organoid transcriptomic datasets
Source: EMBO J. 2022 Aug 2;41(17):e111118. doi: 10.15252/embj.2022111118 (PMC9433936; doi:10.15252/embj.2022111118)
Supplement: Supplementary file 1 — Appendix S1 [file EMBJ-41-e111118-s005.pdf]

Appendix Supplementary Figures

Appendix Figure S1 – WGCNA modules and the limitations of large cluster-based analysis.... 2

Appendix Figure S2 – Differentiation related GO-terms are depleted in stressed cells............. 4

Appendix Figure S3 – Filtering on simple QC metrics is not sufficient for stressed cell exclusion.. 6

Appendix Figure S4 - Mitochondrial and ribosomal balance and degradation pathways in stressed cells.. 8

Appendix Figure S5 – Exclusion of non-telencephalic cells and stressed cells improves dataset quality.. 10

Appendix Figure S6 – Endothelial cell-induction or organoid slicing does resolve stress-identity 12

Appendix Table S1 14

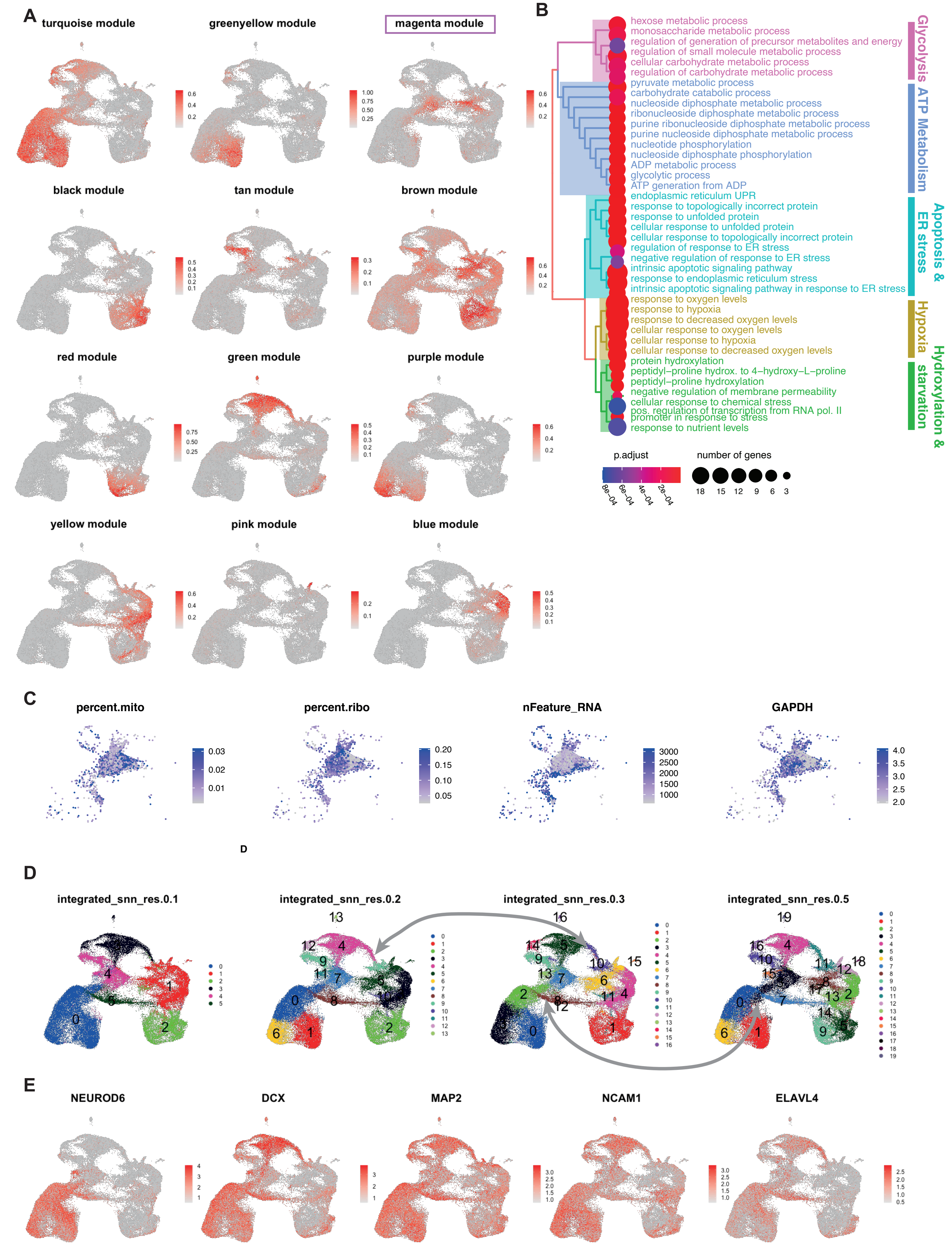
A
turquoise module
greenyellow module
magenta module
black module
tan module
brown module
red module
green module
purple module
yellow module
pink module
blue module
B
hexose metabolic process
monosaccharide metabolic process
regulation of generation of precursor metabolites and energy
regulation of small molecule metabolic process
cellular carbohydrate metabolic process
regulation of carbohydrate metabolic process
pyruvate metabolic process
carbohydrate catabolic process
nucleoside diphosphate metabolic process
ribonucleoside diphosphate metabolic process
purine ribonucleoside diphosphate metabolic process
purine nucleoside diphosphate metabolic process
nucleotide phosphorylation
nucleoside diphosphate phosphorylation
ADP metabolic process
glycolytic process
ATP generation from ADP
endoplasmic reticulum UPR
response to topologically incorrect protein
response to unfolded protein
cellular response to unfolded protein
cellular response to topologically incorrect protein
regulation of response to ER stress
negative regulation of response to ER stress
intrinsic apoptotic signaling pathway
response to endoplasmic reticulum stress
intrinsic apoptotic signaling pathway in response to ER stress
response to oxygen levels
response to hypoxia
response to decreased oxygen levels
cellular response to oxygen levels
cellular response to hypoxia
cellular response to decreased oxygen levels
protein hydroxylation
peptidyl−proline hydrox. to 4−hydroxy−L−proline
peptidyl−proline hydroxylation
negative regulation of membrane permeability
cellular response to chemical stress
pos. regulation of transcription from RNA pol. II promoter in response to stress
response to nutrient levels
Glycolysis
ATP Metabolism
Apoptosis & ER stress
Hypoxia
Hydroxylation & starvation
p.adjust
8e−04
6e−04
4e−04
2e−04
number of genes
18
15
12
9
6
3
C
percent.mito
percent.ribo
nFeature_RNA
GAPDH
D
integrated_snn_res.0.1
integrated_snn_res.0.2
integrated_snn_res.0.3
integrated_snn_res.0.5
E
NEUROD6
DCX
MAP2
NCAM1
ELAVL4

Appendix Figure S1 – WGCNA modules and the limitations of large cluster-based analysis

(**A**) UMAP plots of 12 module-scores identified by single cell WGCNA. (**B**) GSEA of stress module (magenta), related to Figure 1G. (**C**) Heterogeneous composition of the 'stressed-neuron' cluster. The cluster shows salient and complimentary patterns of mitochondrial (I) or ribosomal (II) read fractions, as well as feature count (III). The expression of GAPDH (IV) signals the population of cells with high glycolytic scores. (**D**) Clustering resolutions 0.1 to 0.5 on integrated organoid dataset. Small resolution with large clusters merges stressed and unstressed cell types. With increasing resolution cluster boundaries change and make clear identification of stressed cell clusters difficult. (**E**) Expression of classic or pan-neural markers is diminished in stressed neurons.

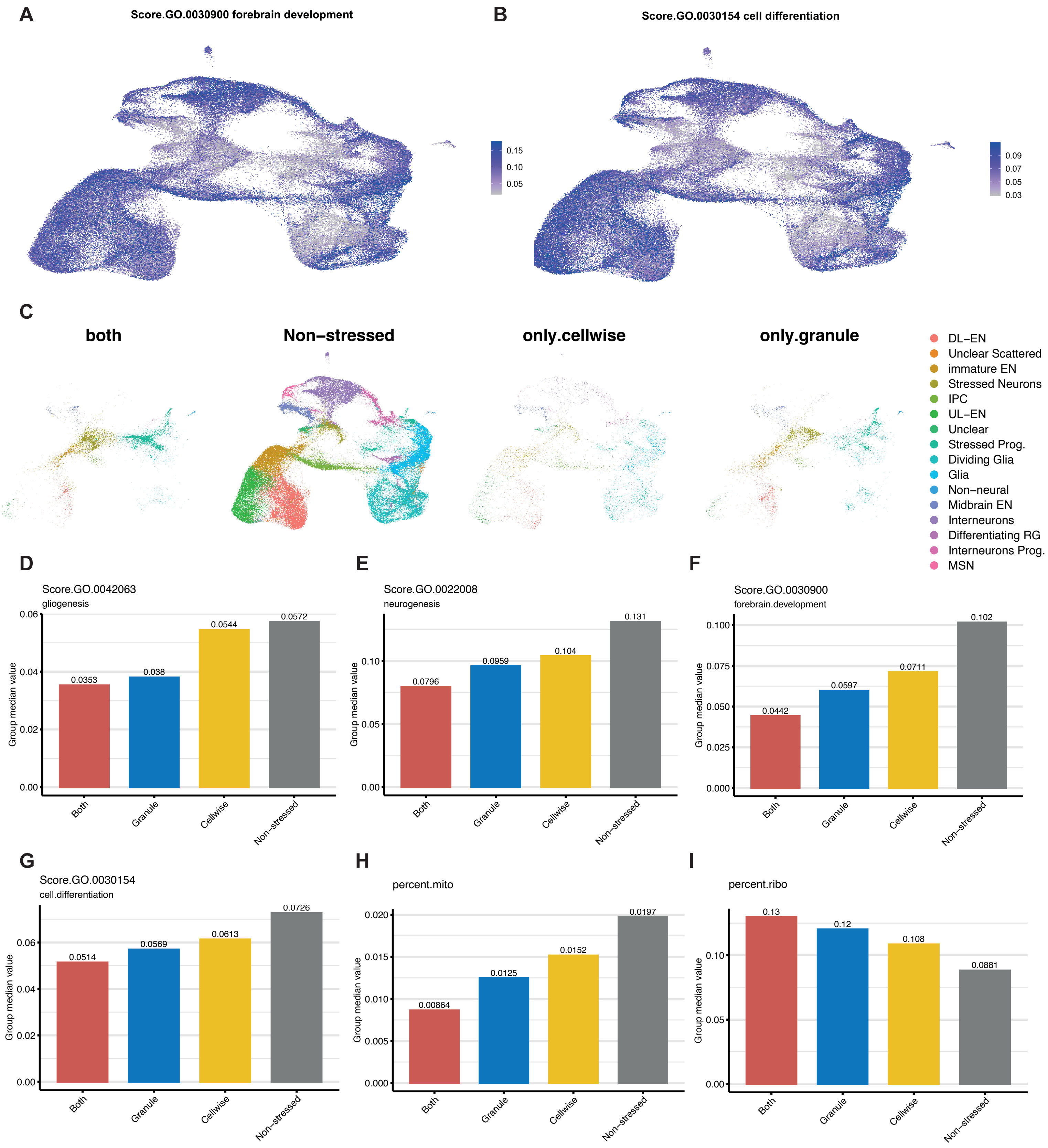
A
Score.GO.0030900 forebrain development
0.15
0.10
0.05
B
Score.GO.0030154 cell differentiation
0.09
0.07
0.05
0.03
C
both
Non−stressed
only.cellwise
only.granule
DL−EN
Unclear Scattered
immature EN
Stressed Neurons
IPC
UL−EN
Unclear
Stressed Prog.
Dividing Glia
Glia
Non−neural
Midbrain EN
Interneurons
Differentiating RG
Interneurons Prog.
MSN
D
Score.GO.0042063
gliogenesis
Group median value
0.0353
0.038
0.0544
0.0572
Both
Granule
Cellwise
Non−stressed
E
Score.GO.0022008
neurogenesis
Group median value
0.0796
0.0959
0.104
0.131
Both
Granule
Cellwise
Non−stressed
F
Score.GO.0030900
forebrain.development
Group median value
0.0442
0.0597
0.0711
0.102
Both
Granule
Cellwise
Non−stressed
G
Score.GO.0030154
cell.differentiation
Group median value
0.0514
0.0569
0.0613
0.0726
Both
Granule
Cellwise
Non−stressed
H
percent.mito
Group median value
0.00864
0.0125
0.0152
0.0197
Both
Granule
Cellwise
Non−stressed
I
percent.ribo
Group median value
0.13
0.12
0.108
0.0881
Both
Granule
Cellwise
Non−stressed

Appendix Figure S2 – Differentiation related GO-terms are depleted in stressed cells

Stressed clusters showed remarkably low pathway scores for 'forebrain development' (GO:0030900) **(A)** and for 'cell differentiation' (GO:0030154) **(B)**. (**C**) UMAP comparison of stressed cells identified by Gruffi's granular method (gSC) and stressed cells identified on single-cell scores (scSC). Cells are colored by clusters as in (**Fig. 1**) and separated by stress identification classes (identified by either, both or neither of the approaches). Class median values for gliogenesis ('GO:0042063', **D**); neurogenesis ('GO:0022008', **E**); forebrain development ('GO:0030900', **F**); cell differentiation ('GO:0030154', **H**); mitochondrial- and ribosomal mRNA content (**H, I**).

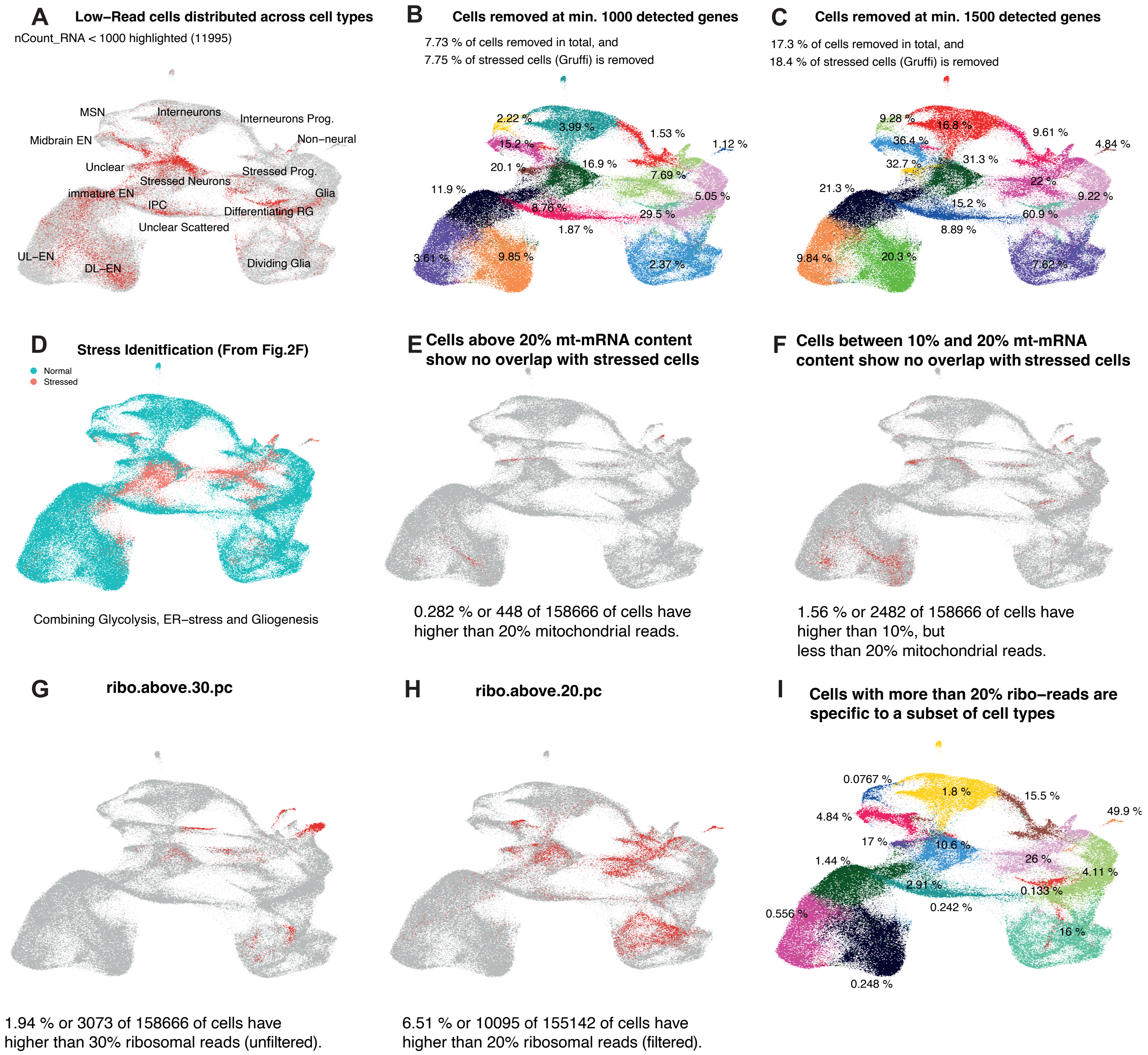
A
Low–Read cells distributed across cell types
nCount_RNA < 1000 highlighted (11995)
MSN
Interneurons
Interneurons Prog.
Midbrain EN
Non–neural
Unclear
Stressed Prog.
Stressed Neurons
immature EN
Glia
IPC
Differentiating RG
Unclear Scattered
UL–EN
DL–EN
Dividing Glia
B
Cells removed at min. 1000 detected genes
7.73 % of cells removed in total, and
7.75 % of stressed cells (Gruffi) is removed
C
Cells removed at min. 1500 detected genes
17.3 % of cells removed in total, and
18.4 % of stressed cells (Gruffi) is removed
D
Stress Idenitfication (From Fig.2F)
Normal
Stressed
Combining Glycolysis, ER–stress and Gliogenesis
E
Cells above 20% mt-mRNA content
show no overlap with stressed cells
0.282 % or 448 of 158666 of cells have
higher than 20% mitochondrial reads.
F
Cells between 10% and 20% mt-mRNA
content show no overlap with stressed cells
1.56 % or 2482 of 158666 of cells have
higher than 10%, but
less than 20% mitochondrial reads.
G
ribo.above.30.pc
1.94 % or 3073 of 158666 of cells have
higher than 30% ribosomal reads (unfiltered).
H
ribo.above.20.pc
6.51 % or 10095 of 155142 of cells have
higher than 20% ribosomal reads (filtered).
I
Cells with more than 20% ribo–reads are
specific to a subset of cell types

Appendix Figure S3 – Filtering on simple QC metrics is not sufficient for stressed cell exclusion

(**A**) Distribution of low-quality cells as defined as cells with less than 1000 genes. (**B**) Percentage of cells that are removed if filtering for high-quality cells (more than 1000 genes). (**C**) Percentage of cells that are removed if filtering for more than 1500 genes. These cutoffs show that stringent read-count cutoffs introduce cell type biases. (**D**) Stress identification of Figure 2F for reference. (**E**) and (**F**) Cells with very-high or high mitochondrial reads show enrichment for specific cell types, but not for stressed cells. (**G**) Cells with very-high (>30%) ribosomal content showed highest overlap with non-telencephalic cells, whereas many cells (**H**), especially glial and dividing cell had high (>20%) ribosomal content (**I**), showing that none of the classic quality metrics are sufficient to identify hypoxic stress in organoids.

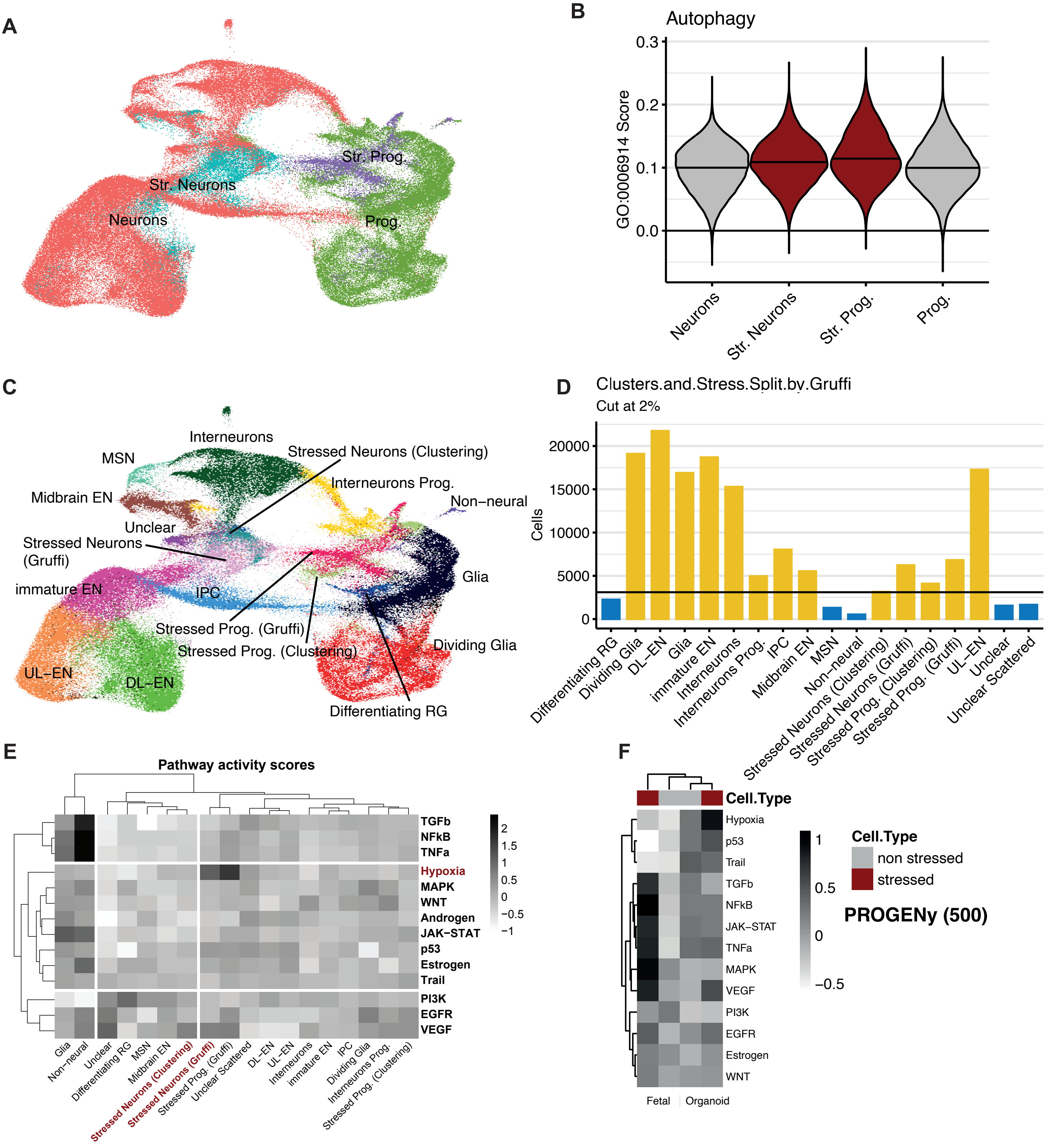
A
Str. Prog.
Str. Neurons
Neurons
Prog.
B
Autophagy
GO:0006914 Score
0.3
0.2
0.1
0.0
Neurons
Str. Neurons
Str. Prog.
Prog.
C
Interneurons
MSN
Stressed Neurons (Clustering)
Interneurons Prog.
Midbrain EN
Non−neural
Unclear
Stressed Neurons
(Gruffi)
immature EN
IPC
Glia
Stressed Prog. (Gruffi)
Dividing Glia
Stressed Prog. (Clustering)
UL−EN
DL−EN
Differentiating RG
D
Clusters.and.Stress.Split.by.Gruffi
Cut at 2%
Cells
20000
15000
10000
5000
0
Differentiating RG
Dividing Glia
DL−EN
Glia
immature EN
Interneurons
Interneurons Prog.
IPC
Midbrain EN
MSN
Non−neural
Stressed Neurons (Clustering)
Stressed Neurons (Gruffi)
Stressed Prog. (Clustering)
Stressed Prog. (Gruffi)
UL−EN
Unclear
Unclear Scattered
E
Pathway activity scores
TGFb
NFkB
TNFa
Hypoxia
MAPK
WNT
Androgen
JAK−STAT
p53
Estrogen
Trail
PI3K
EGFR
VEGF
2
1.5
1
0.5
0
−0.5
−1
Glia
Non−neural
Unclear
Differentiating RG
MSN
Midbrain EN
Stressed Neurons (Clustering)
Stressed Neurons (Gruffi)
Stressed Prog. (Gruffi)
Unclear Scattered
DL−EN
UL−EN
Interneurons
immature EN
IPC
Dividing Glia
Interneurons Prog.
Stressed Prog. (Clustering)
F
Cell.Type
Hypoxia
p53
Trail
TGFb
NFkB
JAK−STAT
TNFa
MAPK
VEGF
PI3K
EGFR
Estrogen
WNT
Fetal
Organoid
1
0.5
0
−0.5
Cell.Type
non stressed
stressed
PROGENy (500)

Appendix Figure S4 - Mitochondrial and ribosomal balance and degradation pathways in stressed cells

(**A**) UMAP classification of cells into four categories corresponding to (**Figure 3A**) and panel **B** here**.** (**B**) Expression of autophagy score in normal and stressed cell populations show minor variation, unlike mitophagy in (**Figure 3A**). (**C**) UMAP showing Gruffi-classified stressed cells separated into stressed vs normal neurons and progenitors, as well as the remaining parts of the initial stress clusters in (**Figure 1A**). (**D**) Cells per cluster. All clusters are shown in next panel here, and (**Figure 3B**) is the same, without the smallest clusters (>2% all cells), for clarity. (**E**) Pathway activity score of all clusters, related to (**Figure 3B**). (**F**) Pathway activity scores of Gruffi-stress classified cells from the 'fetal & organoid' integration. Note that hypoxia activation is much stronger in organoid cells, suggesting that the stress-identity is unique to the *in vitro* condition, and fetal cells are classified only because they were part of the same granule as higher stressed organoid cells.

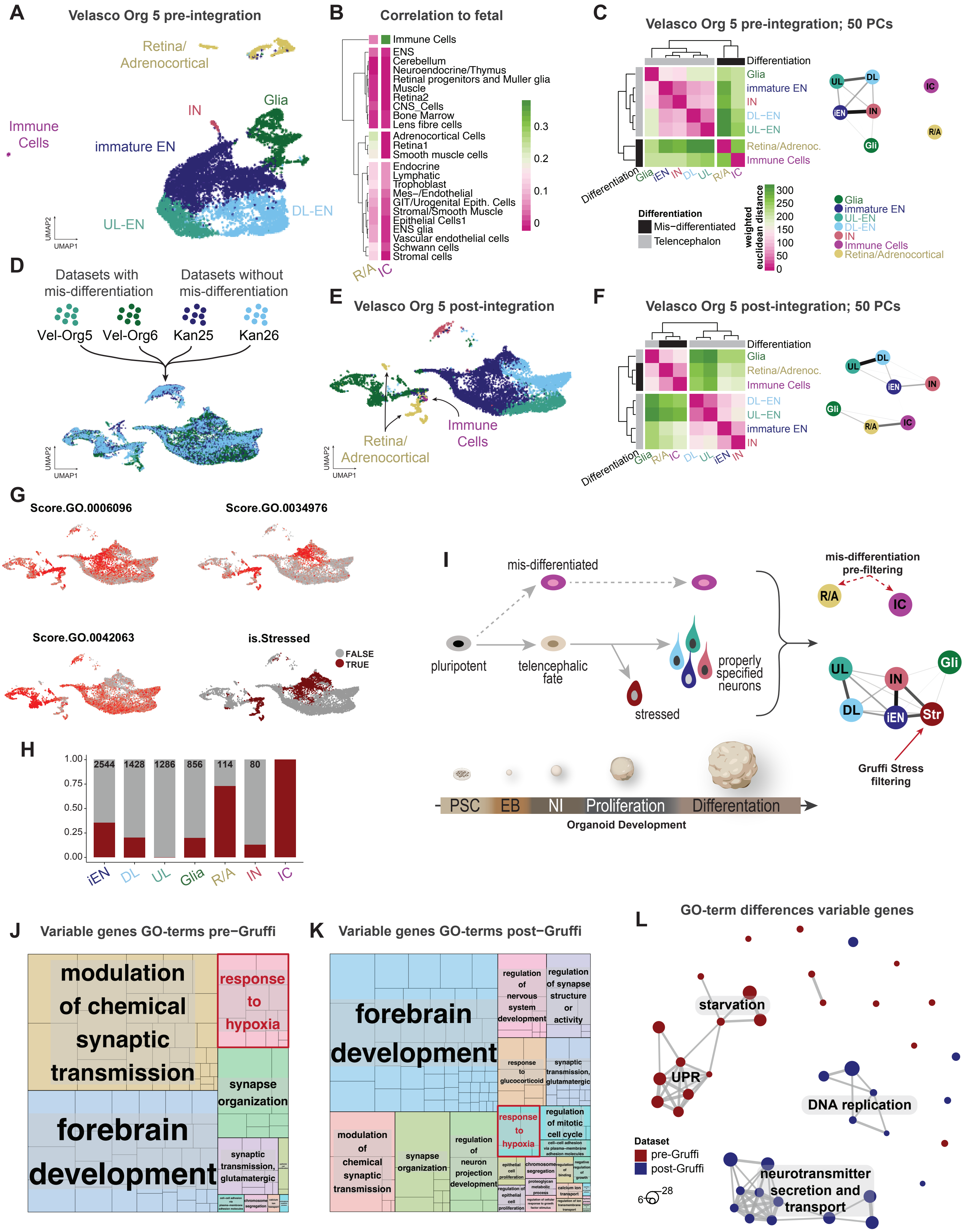

A
Velasco Org 5 pre-integration
Retina/ Adrenocortical
Glia
IN
Immune Cells
immature EN
UL-EN
DL-EN
UMAP2
UMAP1
B
Correlation to fetal
Immune Cells
ENS
Cerebellum
Neuroendocrine/Thymus
Retinal progenitors and Muller glia
Muscle
Retina2
CNS_Cells
Bone Marrow
Lens fibre cells
Adrenocortical Cells
Retina1
Smooth muscle cells
Endocrine
Lymphatic
Trophoblast
Mes−/Endothelial
GIT/Urogenital Epith. Cells
Stromal/Smooth Muscle
Epithelial Cells1
ENS glia
Vascular endothelial cells
Schwann cells
Stromal cells
R/A IC
C
Velasco Org 5 pre-integration; 50 PCs
Differentiation
Glia
immature EN
IN
DL-EN
UL-EN
Retina/Adrenoc.
Immune Cells
Differentiation
Mis−differentiated
Telencephalon
weighted euclidean distance
D
Datasets with mis-differentiation
Datasets without mis-differentiation
Vel-Org5
Vel-Org6
Kan25
Kan26
E
Velasco Org 5 post-integration
Retina/ Adrenocortical
Immune Cells
F
Velasco Org 5 post-integration; 50 PCs
G
Score.GO.0006096
Score.GO.0034976
Score.GO.0042063
is.Stressed
FALSE
TRUE
H
I
mis-differentiated
pluripotent
telencephalic fate
stressed
properly specified neurons
mis-differentiation pre-filtering
Gruffi Stress filtering
PSC EB NI Proliferation Differentation
Organoid Development
J
Variable genes GO-terms pre−Gruffi
modulation of chemical synaptic transmission
response to hypoxia
synapse organization
forebrain development
K
Variable genes GO-terms post−Gruffi
forebrain development
regulation of nervous system development
regulation of synapse structure or activity
response to glucocorticoid
synaptic transmission, glutamatergic
response to hypoxia
regulation of mitotic cell cycle
regulation of neuron projection development
L
GO-term differences variable genes
starvation
UPR
DNA replication
neurotransmitter secretion and transport
Dataset
pre-Gruffi
post-Gruffi

Appendix Figure S5 – Exclusion of non-telencephalic cells and stressed cells improves dataset quality

(**A**) Organoid 5 from (Velasco et al., 2019) projected in UMAP identifies outlier clusters that are classified as non-telencephalic cells. (**B**) Correlation to fetal cells from (Cao et al., 2020) that was also used for training the classifier, which supports the cell type annotation of outlier clusters. (**C**) The weighted Euclidean distance across the top 50 principal components (PCs) between all clusters revealed that the non-telencephalic clusters were very distant from properly specified neuronal lineages and also from each other. This is also evident in a graph network embedding visualizing similarity as edge strength (right) (Epskamp et al., 2012). (**D**) and (**E**) Integration test of datasets with non-telencephalic cells (Vel-5 and -6) with datasets with only telencephalic cells (Kan-25 and -26). The result of integrating without the removal of rare, mis-specified cells resulted in originally distinct outlier clusters clustering close to glial cells and each other. (**F**) Weighted Euclidean distance across the top 50 PCs in a heatmap and graph embedding revealed that after integration non-telencephalic cells were artificially merged with glia. Note, how also the distance between the two mis-differentiated cell types that was very large in (C) is now completely lost (F). (**G**) and (**H**) Stress scoring on integration dataset including non-telencephalic cells demonstrates that stress classification depends on stress pathway activity and not on telencephalic or non-telencephalic fate alone. (**I**) Model for the emergence of mis-differentiated vs. stressed cells: non-telencephalic cells fail to undergo neural induction during early stages of organoid development, whereas stressed cells emerge during later telencephalic lineage progression in organoids. Hence, mis-differentiated cells form defined outlier clusters that can and should be filtered prior to integration, while stressed cells are much closer to properly specified cells and require a more sophisticated approach. (**J**) Before stress exclusion a large fraction of variable features that are used for downstream computation are related to hypoxia response, while the general complexity of the processes that are covered is low. (**K**) After Gruffi hypoxia makes up a smaller fraction of the variable genes' GO-terms and more, and deeper level processes are identified. (**L**) The difference in GO-terms of variable genes is also evidenced by UPR and starvation terms in pre- and neurotransmitter terms in post-Gruffi datasets.

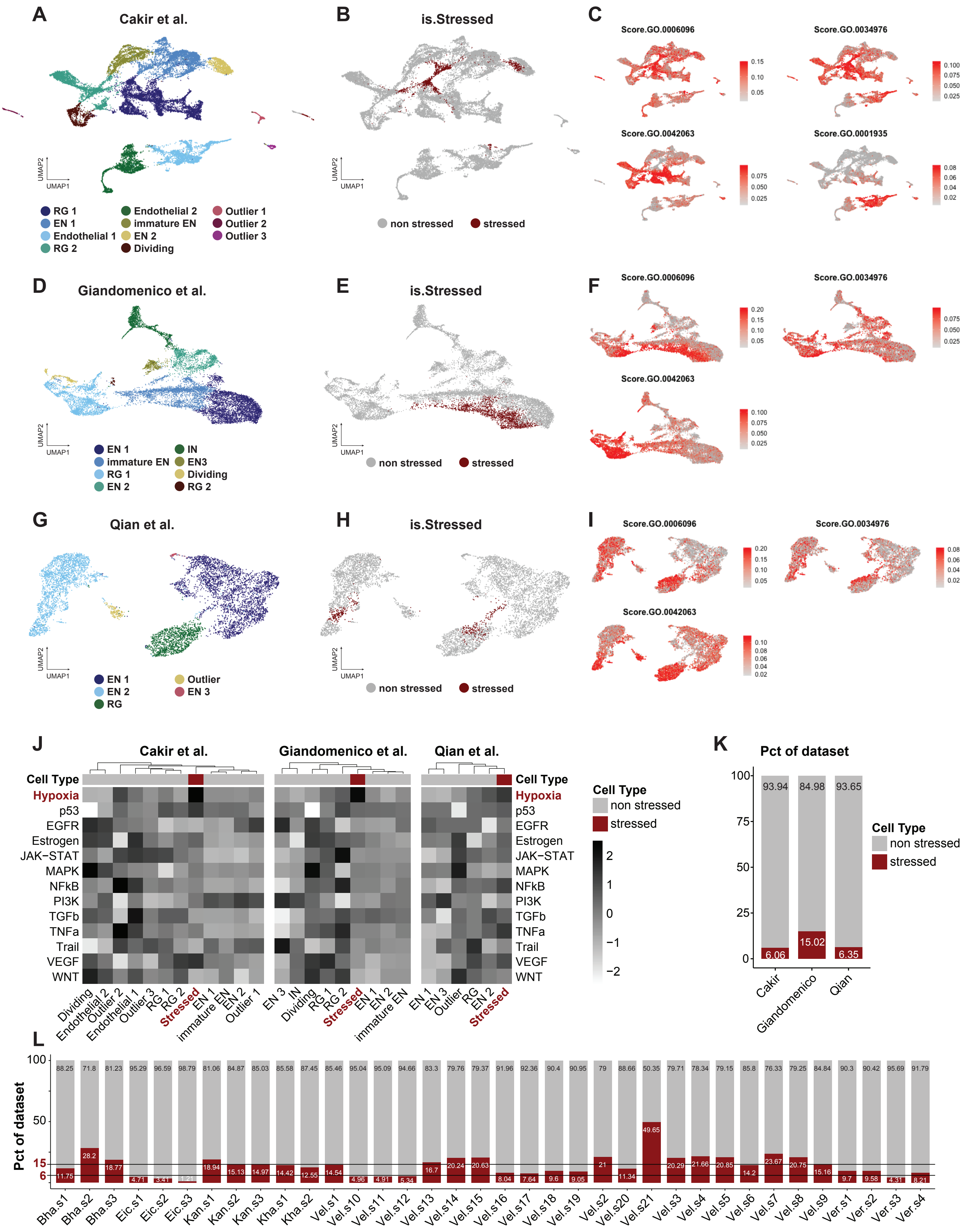
A
Cakir et al.
UMAP1
UMAP2
RG 1
EN 1
Endothelial 1
RG 2
Endothelial 2
immature EN
EN 2
Dividing
Outlier 1
Outlier 2
Outlier 3
B
is.Stressed
non stressed
stressed
C
Score.GO.0006096
Score.GO.0034976
Score.GO.0042063
Score.GO.0001935
D
Giandomenico et al.
EN 1
immature EN
RG 1
EN 2
IN
EN3
Dividing
RG 2
E
is.Stressed
non stressed
stressed
F
Score.GO.0006096
Score.GO.0034976
Score.GO.0042063
G
Qian et al.
EN 1
EN 2
RG
Outlier
EN 3
H
is.Stressed
non stressed
stressed
I
Score.GO.0006096
Score.GO.0034976
Score.GO.0042063
J
Cakir et al.
Giandomenico et al.
Qian et al.
Cell Type
Hypoxia
p53
EGFR
Estrogen
JAK−STAT
MAPK
NFkB
PI3K
TGFb
TNFa
Trail
VEGF
WNT
Cell Type
non stressed
stressed
Dividing
Endothelial 2
Outlier 2
Endothelial 1
Outlier 3
RG 1
RG 2
Stressed
EN 1
immature EN
EN 2
Outlier 1
EN 3
IN
Dividing
RG 1
RG 2
Stressed
EN 1
EN 2
immature EN
EN 1
EN 3
Outlier
RG
EN 2
Stressed
K
Pct of dataset
93.94
84.98
93.65
6.06
15.02
6.35
Cakir
Giandomenico
Qian
Cell Type
non stressed
stressed
L
Pct of dataset
Bha.s1 88.25 11.75
Bha.s2 71.8 28.2
Bha.s3 81.23 18.77
Eic.s1 95.29 4.71
Eic.s2 96.59 3.41
Eic.s3 98.79 1.21
Kan.s1 81.06 18.94
Kan.s2 84.87 15.13
Kan.s3 85.03 14.97
Kha.s1 85.58 14.42
Kha.s2 87.45 12.55
Vel.s1 85.46 14.54
Vel.s10 95.04 4.96
Vel.s11 95.09 4.91
Vel.s12 94.66 5.34
Vel.s13 83.3 16.7
Vel.s14 79.76 20.24
Vel.s15 79.37 20.63
Vel.s16 91.96 8.04
Vel.s17 92.36 7.64
Vel.s18 90.4 9.6
Vel.s19 90.95 9.05
Vel.s2 79 21
Vel.s20 88.66 11.34
Vel.s21 50.35 49.65
Vel.s3 79.71 20.29
Vel.s4 78.34 21.66
Vel.s5 79.15 20.85
Vel.s6 85.8 14.2
Vel.s7 76.33 23.67
Vel.s8 79.25 20.75
Vel.s9 84.84 15.16
Ver.s1 90.3 9.7
Ver.s2 90.42 9.58
Ver.s3 95.69 4.31
Ver.s4 91.79 8.21

Appendix Figure S6 – Endothelial cell-induction or organoid slicing does resolve stress-identity

(A) UMAP projection of the (Cakir et al., 2019) dataset including telencephalic cells (RGs and ENs), as well as putative endothelial cells. (**B**) Gruffi identified stressed cells among the telencephalic lineages; notably, endothelial cell clusters are not classified as stressed. (**C**) Expressions of single GO terms supports Gruffi stress identification. Putative endothelial cell clusters express the GO term GO:0001935 “endothelial cell proliferation”, also used by (Cakir et al. 2019). (**D**) UMAP projection of the (Giandomenico et al., 2019) dataset, generated from organoids sliced only once. The dataset contains only telencephalic lineages (RGs, EN, IN). (**E**) and (**F**) Gruffi identified stressed cells next to the normal telencephalic lineage (F), further supported by individual GO scores (F). (**G**) UMAP projection of (Qian et al., 2020) dataset generated from organoids with repeated slicing, containing only excitatory neuronal lineages (RGs, EN). (**H**) and (**I**) Gruffi identified stressed cells, but less than in (E), further supported by individual GO scores (I). (**J**) Confirmatory analysis with PROGENy. Pathway scores on all three datasets supports the Gruffi stress annotation, as stressed cells in all three datasets score highly on “Hypoxia”. (**K**) and (**L**) The abundance of stressed cells in these protocols (K) was comparable to organoids grown in standard conditions (L).

Str. Neurons	11.60	1.16	0.02	0.07	0.10
Neurons	8.31	2.40	-0.03	0.02	0.10
Str. Prog.	14.92	1.98	0.04	0.10	0.11
Prog.	12.08	2.63	-0.01	0.04	0.12
Increase / Decrease					
Str.N./ N.	140%	48%	-64%	363%	97%
Str.P./P.	123%	75%	-302%	255%	93%

Appendix Table S1

Comparison of ribosomal, mitochondrial reads and stress scores in stressed and unstressed clusters.
