# Supplementary material for: Gruffi: an algorithm for computational removal of stressed cells from brain organoid transcriptomic datasets
Source: EMBO J. 2022 Aug 2;41(17):e111118. doi: 10.15252/embj.2022111118 (PMC9433936; doi:10.15252/embj.2022111118)
Supplement: Supplementary file 2 — Expanded View Figures PDF [file EMBJ-41-e111118-s004.pdf]

Expanded View Figures

Figure EV1. Metabolic changes and marker genes in stressed cells.

A UMAP of organoid integration shown in Fig 1B colored by number of RNA features per cell (nFeature_RNA).

B Low-quality (LQ) cells as determined by expression of < 1,000 features. In the background the clustering of Fig 1B is shown. On top per cluster the percentage of LQ cells per cluster (color) and the number of LQ cells (size) are depicted.

C Expression of additional endoplasmic reticulum (ER) stress genes enriched in the stress clusters.

D Expression of additional glycolysis genes enriched in the stress clusters.

E Vimentin (*VIM*) is expressed in all progenitor populations regardless of lineage or stress state.

F, G Additional GO-terms scores 'response to starvation' (GO:0042594) and 'cellular response to hypoxia' (GO:0071456) are also characteristic of stressed cells.

H–J Stress cluster marker genes in relevant significantly associated KEGG pathways: HIF-1α signaling, (Genes: 12, Fold Enrichment: 14.4, FDR: 2.30e-9); Glycolysis, (7, 13.7, 2.3e-5); Protein processing in the ER, (8, 5.7, 1.9e-3). The top 150 coding stress marker genes were used for this analysis (as in Fig 1E). Enriched genes are marked red.

© 2022 The Authors

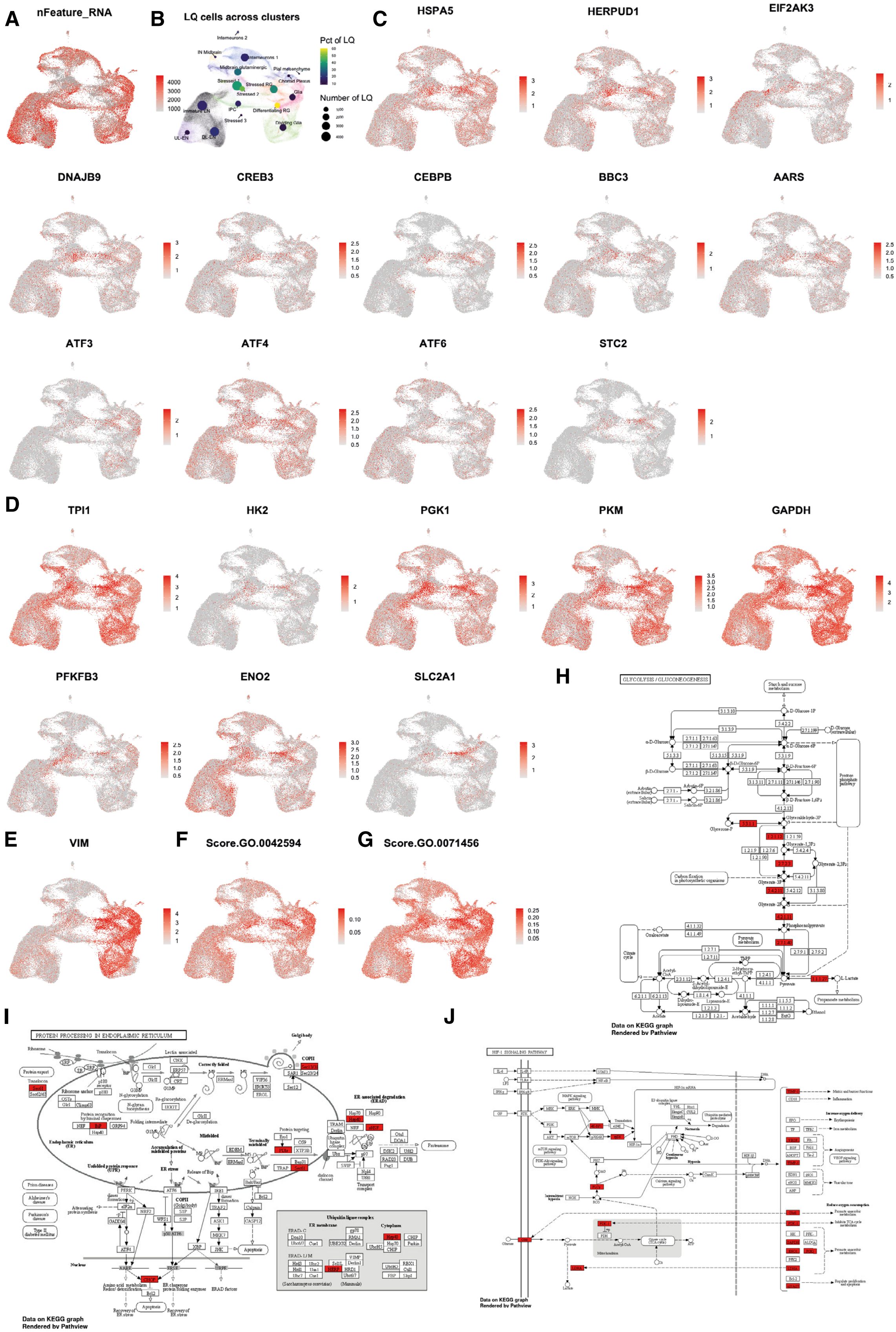

Figure EV1.

© 2022 The Authors

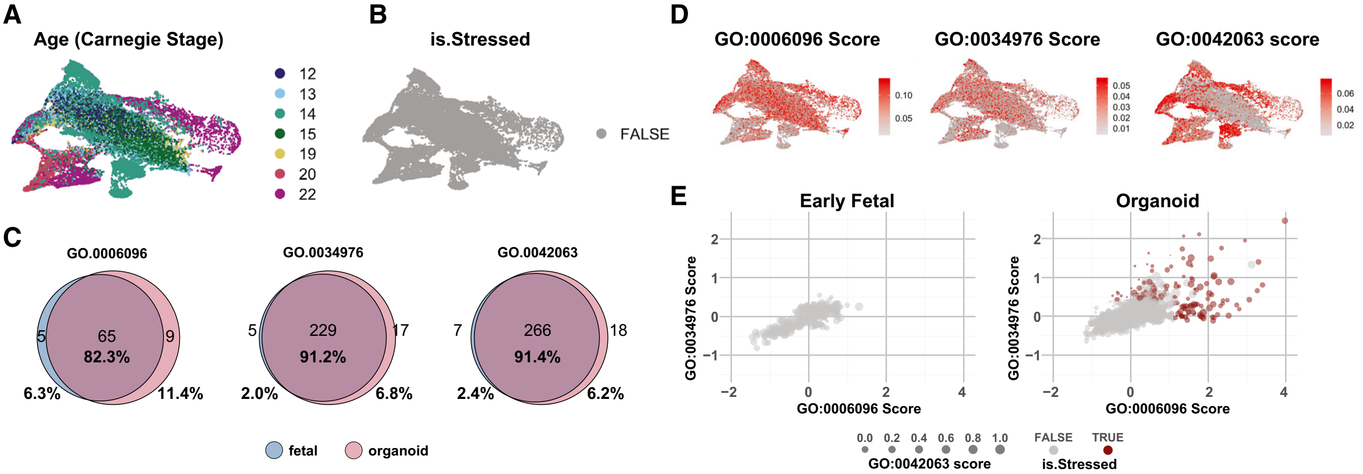

Figure EV2. Early radial glia are not stressed.

A UMAP of early radial glia cells from (Eze *et al*, 2021) color-coded for age in Carnegie stages.

B Gruffi stress-classification on early radial glia does not detect any stressed cells.

C Comparison genes expressed with the GO-terms used for stress classification. The large overlap between fetal and organoid datasets shows that it is not a difference in expressed genes that underlies the lack of stress identified in Eze *et al*, thus, excluding a technical or sequencing depth bias in GO scores.

D Per-cell glycolysis, ER-stress and gliogenesis scores.

E Per-granule scores for glycolysis (*x*) and ER-stress (*y*) in early fetal and organoid datasets. Stress scores are generally low in the fetal sample, whereas in organoids, stress-classified granules are outliers with high scores. Each granule is sized by the expression of the gliogenesis score, which reveals that individual clusters with high glycolysis and ER-stress that were not classified as stressed in the organoid are indeed gliogenic.

© 2022 The Authors

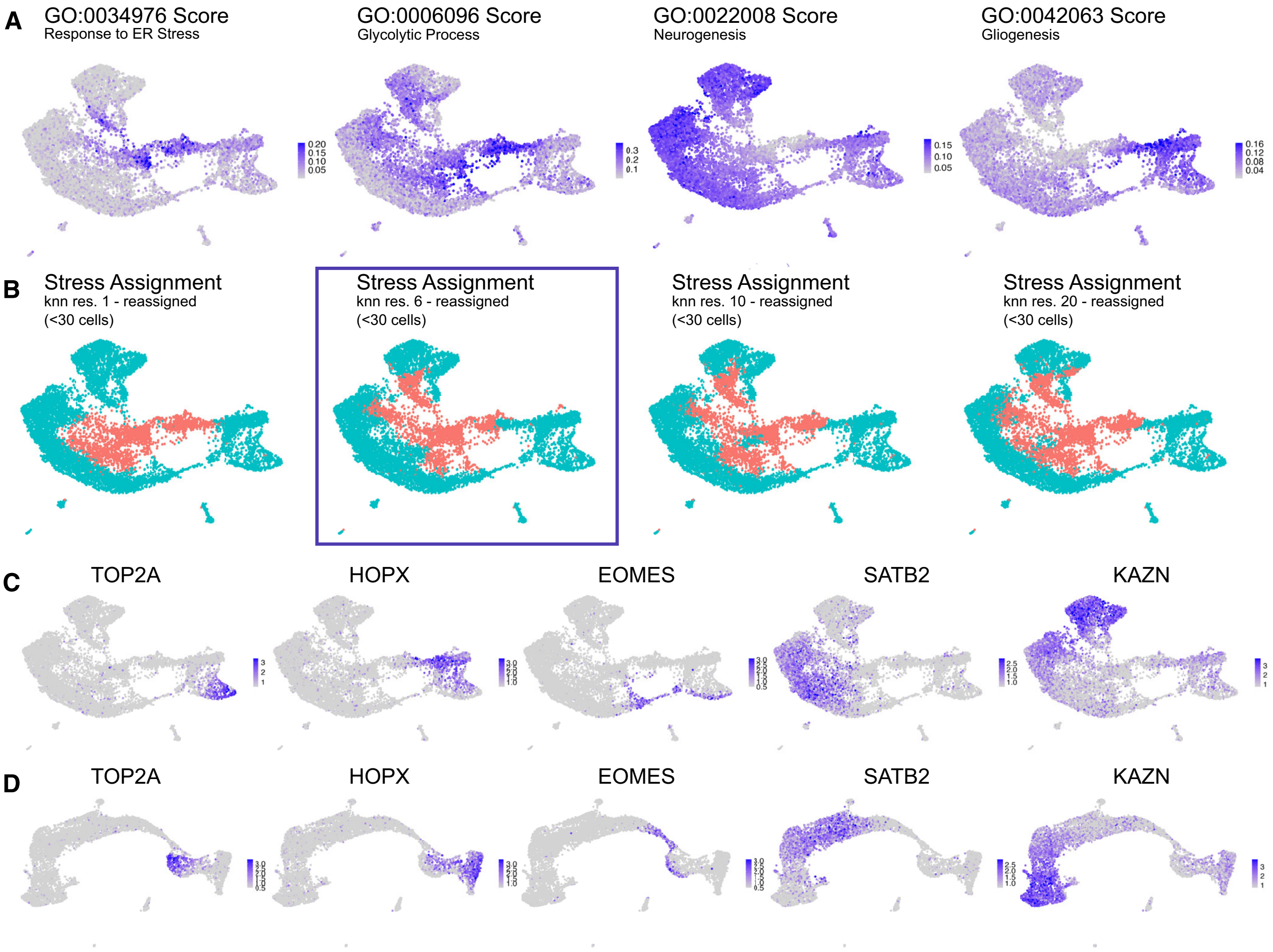

Figure EV3. Benchmarking Gruffi on a single-experiment sized dataset.

A UMAP plots of one dataset (Velasco 7) cell-wise GO score for response to Endoplasmic Reticulum stress (GO:0034976), glycolytic process (GO:0006096) and gliogenesis (GO:0042063).

B Stress assignment performed on k-nearest-neighbor clustering with resolution 1, 6, 10 and 20 plus reassignment of granules with a cell count below 30 (left to right). Resolution 6 (+ reassignment, the proposed resolution for a median cell number between 100 and 200 cells, see Materials and Methods), resulted in 53 granules with a median cell number of 197.

C, D Expression profiles of markers for progenitor cells (*TOP2A*, *HOPX*), Intermediate Progenitors (*EOMES*), upper layer excitatory neurons (*SATB2*) and deep layer excitatory neurons (*KAZN*) show that the developmental trajectory is refined in a newly computed UMAP after stress filtering (D) compared to before stress filtering (C). For the new UMAP, we recomputed (and scaled) the most variable genes, Principal Components and the UMAP embedding.

 © 2022 The Authors

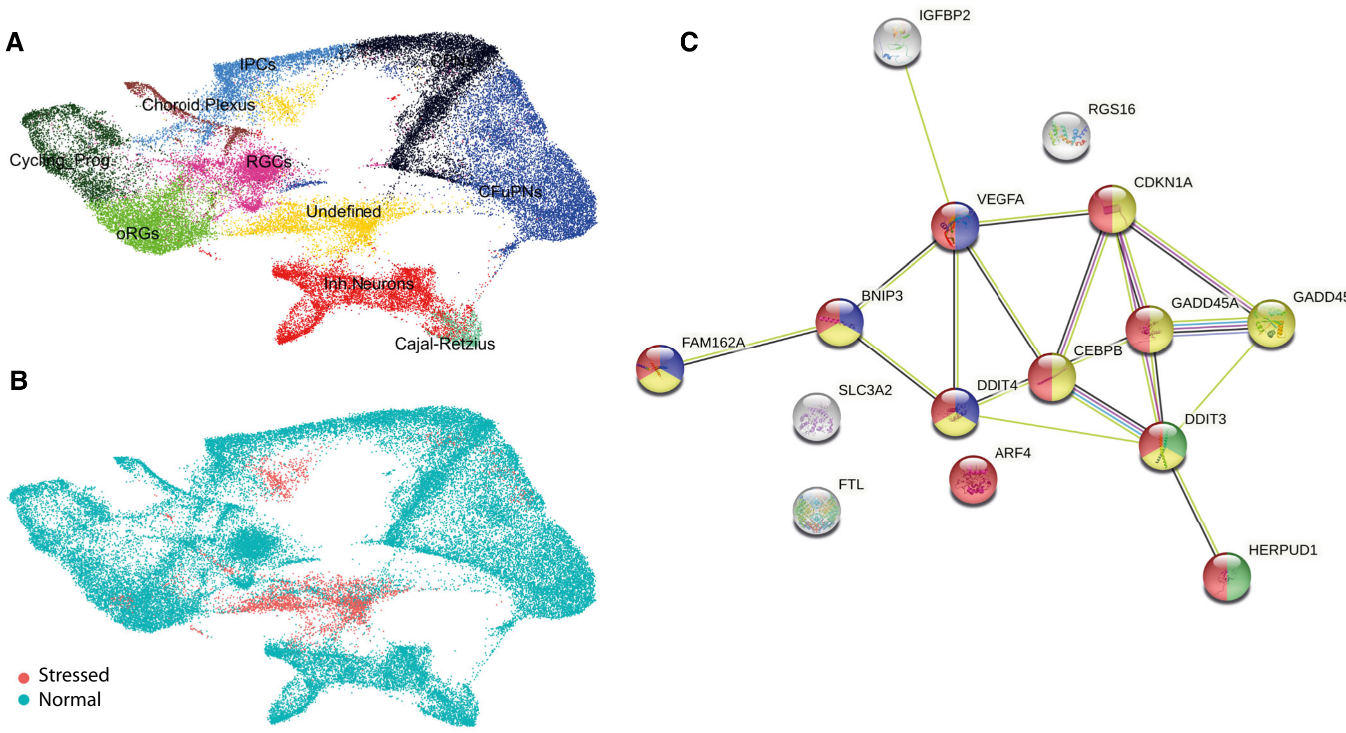

Figure EV4. Stress identification in Samarasinghe *et al* (2021).

A Reproduction of Fig 4A UMAP in (Samarasinghe *et al*, 2021).
B Stress assignment by Gruffi using res.25 (auto determined resolution, 260 granules).
C Protein interaction map of marker genes of stress cells. Response to stress (red, GO:0006950, 0.02); Apoptotic process (yellow, GO:0006915, FDR = 0.0003); PERK-mediated unfolded protein response (limegreen, GO:0036499, 0.0221); Response to hypoxia (blue, GO:0001666, 0.0334); DGEA and all enrichment terms are in (Dataset EV5).

Figure EV5. Proper specification and maturation in non-stressed cells.

A Progenitor (*x*-axis) and excitatory neuron scores (*y*-axis) calculated in fetal brain datasets across brain development (Materials and Methods, Bhaduri *et al*, 2020). Early datasets from Carnegie stage 13 (CS13) are enriched in progenitors, while late datasets from gestational week (GW) 18 and 22 show both neurons and progenitors.
B Progenitor (*x*-axis) and excitatory neuron scores (*y*-axis) applied to multiple datasets of mid-gestation (Fig 4F) show reliable separation of neurons and progenitors, while only intermediate progenitors (IPCs, red) cluster in between the two populations.
C–F Examples of pre- and post-filtering plots for subtype specification of individual datasets analyzed in this study. After filtering out stressed cells only IPCs remain in between neurons and progenitors.
G 3D UMAP of dorsal lineage also shown in Fig 4J colored for pseudotime.
H Expression of the deep layer excitatory neuron (DL-EN) gene module (Table EV5) is specifically enriched in the DL-EN cluster (Cluster 9 in Fig 4J).
I Expression of the upper layer excitatory neuron (UL-EN) gene module (Table EV5) is specifically enriched in the UL-EN cluster (Cluster 8 in Fig 4J).
J Maturation of UL-EN in two different media formulations analogous to DL-EN maturation in Fig 4L. Cells are color coded for clusters (Fig 4J) and plotted along pseudotime (*x*-axis). The density-count of cells along pseudotime is shown behind the dots (yellow area for H-medium, purple area for L-medium, right *y*-axis). The position of the points reflects the expression of a module of co-regulated genes enriched in UL-EN (left *y*-axis, Fig EV5I). While proper maturation indicated by expression of the UL-EN module occurs in both media conditions, in L-medium maturation is much more frequent.
K Distribution of cells (black lines) and densities (areas) of individual organoids across pseudotime. Organoids derived from the same cell line (A to D) grown in the different media conditions (yellow for H-medium, purple for L-medium) are shown on top of each other.
L Cluster contributions per individual organoids (as shown in Fig EV5K). The increased maturation in L-medium organoids is also reflected by higher proportions of mature cell types (UL- and DL-EN) in L-medium organoids. Cell numbers were downsampled to account for different library sizes.

© 2022 The Authors

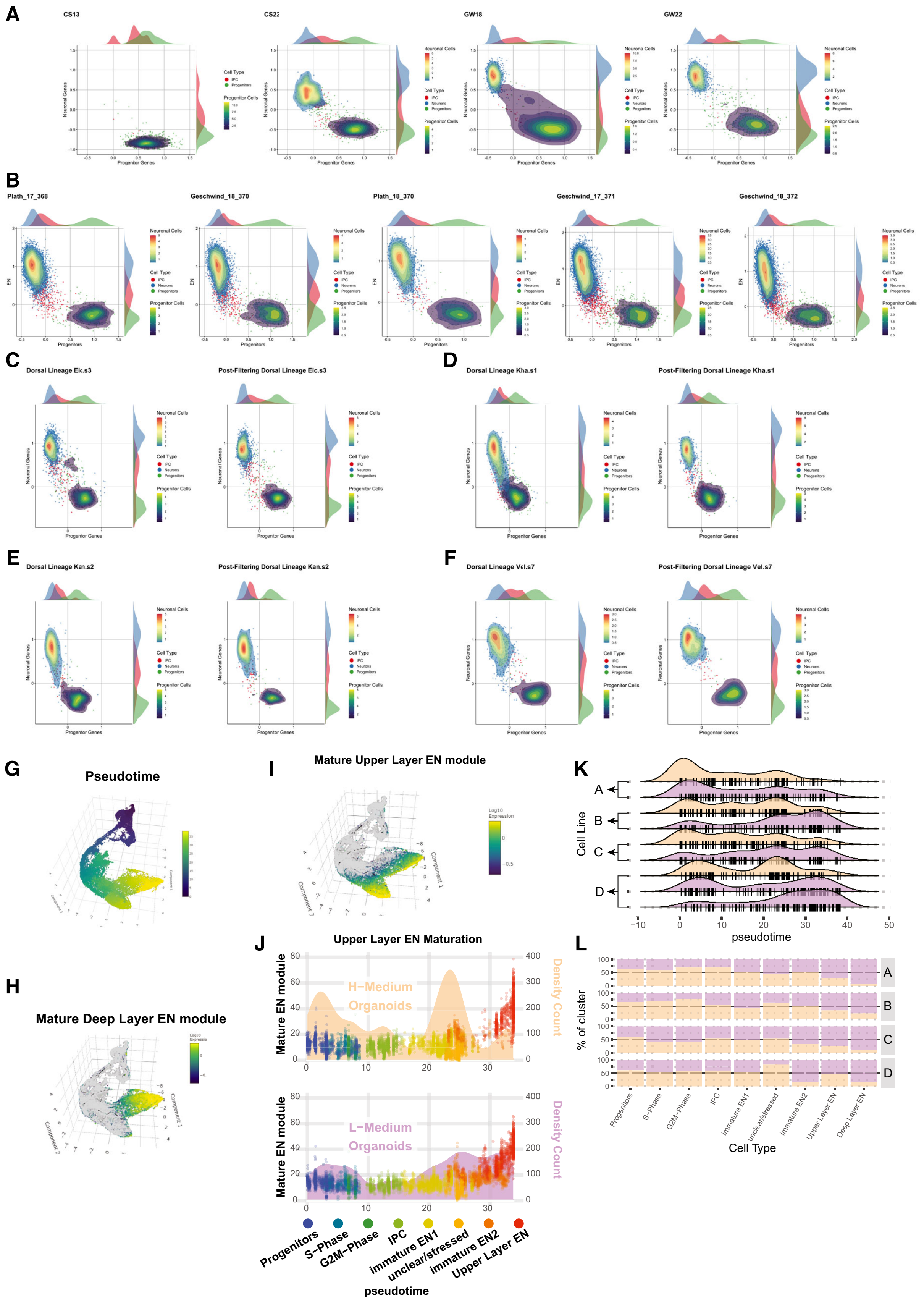

Figure EV5.

 © 2022 The Authors

Figure EV6. Gruffi identifies stressed cells in retinal organoids.

A UMAP showing CCA integrated and re-clustered datasets as in analysis of fig 6 in Sridhar *et al* (2020) and colored based on annotation provided by the authors.
B UMAP color coded for datasets: two fetal (125 days central and periphery) and one organoid dataset (205 days).
C Bar plot with downsampled contribution per cluster. There are three clusters mostly consisting of retinal organoid cells.
D GO-term scores as used previously in Gruffi. Note that glycolysis is very high in RGCs, a cell type known to be very metabolically active. Additionally, a score for RGCs is shown consisting of the marker genes: *ISL1*, *SNCG*, *RBPMS*.
E scWGCNA analysis on retinal data identifies one module (blue module) that is enriched in organoid cells.
F, G GSEA of blue module from scWGCNA reveals enrichment of hypoxia related terms in this module.
H The GO-term "cellular response to hypoxia" (GO:0071456) marks specific populations in the dataset and will be used in the stress annotation.
I Gruffi stress identification using response to hypoxia and to ER stress as selecting terms, as well as gliogenesis and the RGC score as negative filters. Color code shows stressed cells and UMAP depicts each different dataset.
J The three clusters that were biased toward organoid cells in (C) show strong enrichment in stressed cells, whereas other clusters have generally low percentages (left). Similarly, the organoid dataset shows about 33% of stressed cells, while in fetal there are almost none (right).
K Per granule score of GO-terms with response to hypoxia on the *x*-axis and response to ER stress on the *y* axis shows that stress scores are generally low in fetal but increased in organoids. The size depicts the number of cells that are within the respective granule.
L PROGENy pathway activity score for the clusters as shown in L reveals that Hypoxia is uniquely enriched in stressed cells. Note that miG is a remnant of prior cell-type annotation and contains just 36 cells. It is therefore to be regarded as an unrepresentative leftover population.
M, N After stress filtering the datasets are re integrated and all downstream visualizations are re-computed.

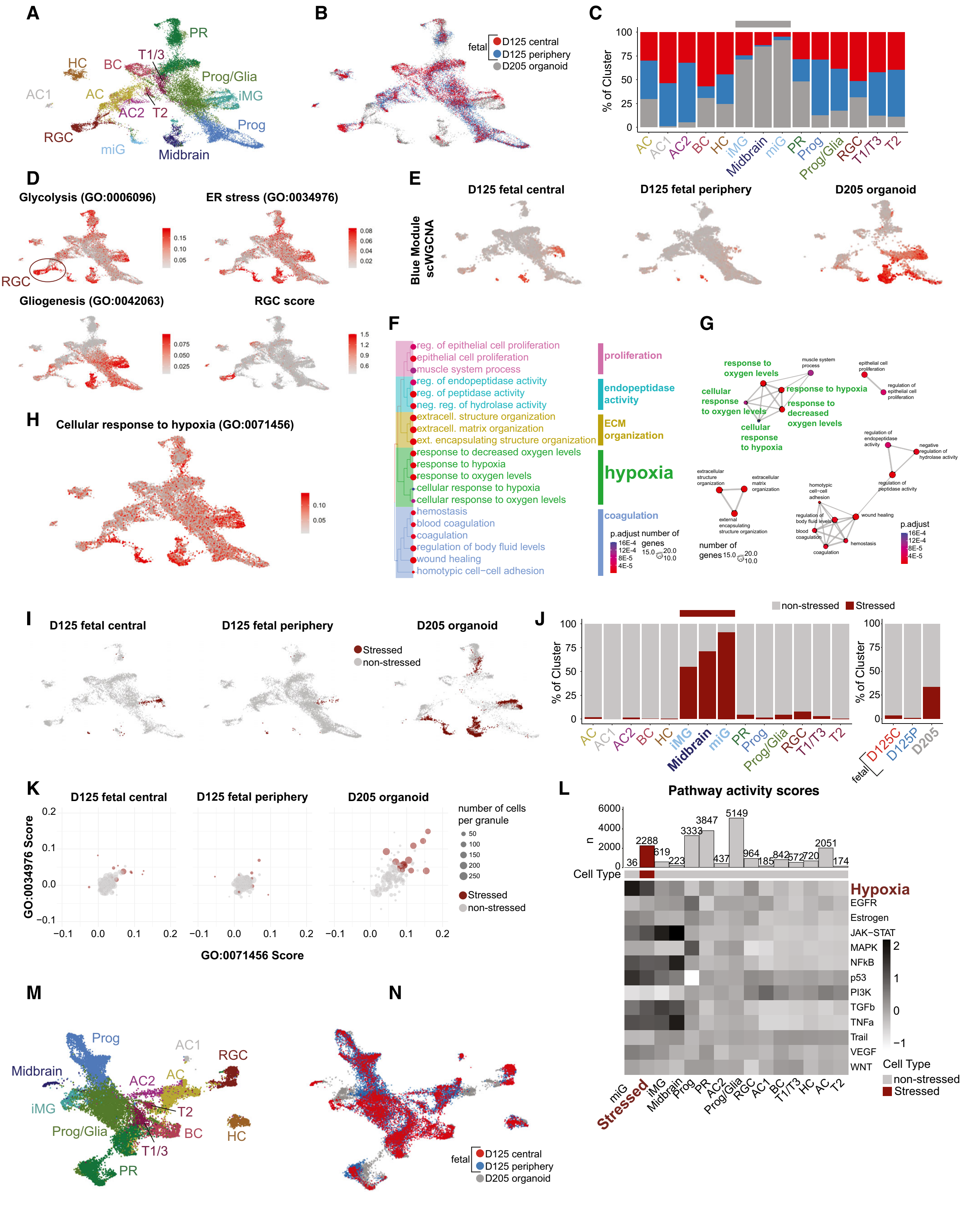

Figure EV6.

    © 2022 The Authors
