# Supplementary material for: Gruffi: an algorithm for computational removal of stressed cells from brain organoid transcriptomic datasets
Source: EMBO J. 2022 Aug 2;41(17):e111118. doi: 10.15252/embj.2022111118 (PMC9433936; doi:10.15252/embj.2022111118)
Supplement: Supplementary file 8 [file EMBJ-41-e111118-s007.pdf]

THE EMBO JOURNAL

Gruffi: An algorithm for computational removal of stressed cells from brain organoid transcriptomic datasets

Abel Vertesy, Oliver Eichmueller, Julia Naas, Maria Novatchkova, Christopher Esk, Meritxell Balmaña, Sabrina Ladstätter, Christoph Bock, Arndt von Haeseler, and Juergen Knoblich
DOI: 10.15252/embj.2022111118

Corresponding author(s): Juergen Knoblich (juergen.knoblich@imba.oeaw.ac.at)

Review Timeline:

Submission Date:	9th Mar 22
Editorial Decision:	21st Apr 22
Revision Received:	23rd May 22
Editorial Decision:	27th Jun 22
Revision Received:	30th Jun 22
Accepted:	4th Jul 22

Editor: Karin Dumstrei

Transaction Report:

(Note: With the exception of the correction of typographical or spelling errors that could be a source of ambiguity, letters and reports are not edited. Depending on transfer agreements, referee reports obtained elsewhere may or may not be included in this compilation. Referee reports are anonymous unless the Referee chooses to sign their reports.)

1st Editorial Decision 21st Apr 2022

Dear Jürgen,

Thank you for submitting your manuscript to The EMBO Journal. Your study has now been seen by two referees and their comments are provided below. I had also involved a third referee, but as I have not heard back from this referee I will go ahead with the two reports on hand.

As you can see from the comments below the referees find the analysis interesting and insightful. Should you be able to address the raised concerns then I would like to invite you to submit a revised version.

I think it would be helpful to discuss the raised points further and I am available to do so via email or video.

When preparing your letter of response to the referees' comments, please bear in mind that this will form part of the Review Process File, and will therefore be available online to the community. For more details on our Transparent Editorial Process, please visit our website: https://www.embopress.org/page/journal/14602075/authorguide#transparentprocess

We generally allow three months as standard revision time. As a matter of policy, competing manuscripts published during this period will not negatively impact on our assessment of the conceptual advance presented by your study. However, we request that you contact the editor as soon as possible upon publication of any related work, to discuss how to proceed. Should you foresee a problem in meeting this three-month deadline, please let us know in advance and we may be able to grant an extension.

Thank you for the opportunity to consider your work for publication. I look forward to your revision.

with best wishes

Karin

Karin Dumstrei, PhD
Senior Editor
The EMBO Journal

Instructions for preparing your revised manuscript:

I have attached a document with helpful tips on how to prepare the revised version.

Guide For Authors: https://www.embopress.org/page/journal/14602075/authorguide

We realize that it is difficult to revise to a specific deadline. In the interest of protecting the conceptual advance provided by the work, we recommend a revision within 3 months (20th Jul 2022). Please discuss the revision progress ahead of this time with the editor if you require more time to complete the revisions. Use the link below to submit your revision:

https://emboj.msubmit.net/cgi-bin/main.plex

Referee #1:

Brain organoids are generated from human pluripotent stem cells, and have been used for a number of researches in investigating human brain development and diseases. However, they are artificial structures, and raise concerns in formation of cells of non-physiology. Vertesy et al. reanalyzed the published scRNA-seq from brain organoids as wells as generated their own data, and searched for the artifacts in the brain organoids. They found that there are clusters of cells with signature for stress, including the ER stress, glycolysis, and hypoxia. They developed analytical method, called Gruffi, which remove the stressed cells and showed that after filtering out these cells, the remaining cells better represent the developmental trajectories and mature cell formation. The manuscript deals with the important questions in the brain organoids field, and the analysis seems well performed. However, there are some issues that need addressed.

1. When the scRNA-seq data are analyzed, it is critical to perform Q/C to filter out the dead, damaged, or dying cells. Authors present data from filtering cells > 500 genes, < 20% mito, and < 30 rRNA reads. The major portion of the stressed cells are at the category of the cells < 20 % but > 10% of mito reads. The cells with higher mito-reads represent those undergoing apoptosis, and thus including the cells with higher mito-read may have resulted in the stressed cells with GO for response to stress, apoptosis, and glycolysis. Author should present the UMAP with different sets of the filtering to present which are the

major factors of stressed cells, and thus in future investigators consider the use of the filters. When the Gruffi is applied to these more stringent conditions, how the stressed cells are distributed, and what are the percentage?

2. In addition, authors filtered out non-neuronal cells to conclude that the stressed cells standout in clusters, and then the stressed cells are not neurons in conclusion. It is contradictory. Authors should present what the unbiased clusters like without filtering out non-neuronal cells, how much overlap the stressed cells make with those filtered non-neuronal cells, and belong to which cell types. Along the same line, the primary brain data also being filtered for the non-neural cells, the authors reach conclusion of the absence of stressed cells. Authors include all cells from the organoids and primary cells of fetal brain data, and show which cells the stressed cells represent.

3. One of major question in the paper is "Does stress occur in all or only certain organoid protocols?" But, authors only limited the datasets from the traditional organoids methods. Authors should test whether the stressed clusters are present, or become minimal at the setting where organoids were made with less hypoxic condition. Data from 1) Qian, et al, Cell Stem Cell, 2020, 2) Cakir, et al, Nature Methods, and 3) Giandomenico, et al, Nature Neuroscience, 2019, should be obtained and tested their hypothesis, and use Gruffi to see what are the impact of the methods in these three papers to reduce the stress-induced genes and cells.

Minor concerns
1. There are a few figures that contain non-relevant contents, like coordinates of x and y, like Figure 3B and 4G. It seems from the cropping of desktop figures. Please replace them.

Referee #2:

The manuscript titled "Cellular stress in brain organoids is limited to a distinct and bioinformatically removable subpopulation" by Vertesy et al. identified stress signature in a defined subset of cells in brain organoids. Removing the cells with these stress signatures improved overall data quality and revealed clear developmental trajectories. This manuscript addresses a pressing question in the organoid field and will be of wide impact: whether cellular stress affects organoid development generally and whether these stressed cells affect overall organoid development. This is an open question in the field of brain organoids specifically and the entire organoid field more broadly. Below, I have some suggestions for improvement for the authors.

Major issues:

1. The authors claim that the "that stressed cells are in a more 'basal' state compared to differentiating neurons". The authors need to be more exact as to what this 'basal' state is? Do these cells show higher expression of markers for less differentiated cell? Do they show higher expression of stem cell markers?
2. The authors claim there is a switch in cell fate in the stressed cells: "Thus, chronic stress in organoids comes at the expense of neurogenic cell differentiation and leads to a switch in cell fate". However, they don't actually show that the cell fate actually switches as opposed to being less differentiated. A switch in cell fate would show up on the trajectory analysis as a separate trajectory, which is not the case.
3. It is unclear how Granules were constructed and how Gruffi finds and optimal finds an optimal granule resolution. This should be more explicitly discussed in the methods section.
4. In various parts of the manuscript the authors rely on UMAP distance to make claims about the difference between cells (including in how they merge granules). This is certainly better than tSNE, but not entirely correct, as UMAP is a method that distorts the underlying data/distances making the distances somewhat arbitrary as discussed here: https://www.biorxiv.org/content/10.1101/2021.08.25.457696v3
5. The authors state that the single cell approach is suitable for identifying correctly specified cells with high levels of stress. How do the authors explain the high levels of stress in these cells? What is causing these cells stress and why are they less differentiated than the other cells in the cluster? Also, can the authors explain why are the scSC are not more abundant (enriched?) in the stress clusters. Could it be that these are not really stressed, but just different maturation trajectories?
6. In Figure 3A are changes significant across the autophagy/ubiquitin pathways? What comparisons were made?
7. In the section describing figure 3b hypoxia is only enriched in Gruffi identified cells and not in the naïve clusters. Could the authors comment on why these naïve clusters are not enriched for hypoxia as they contain appear to highly overlap with the Gruffi clusters. One concern is that as Gruffi uses similar stress terms to define the cells there may be confirmation bias that the authors and defining and measuring the Gruffi results based on the same or similar genes.
8. While the authors claim the section title that "Stressed cells in organoids have no fetal counterpart" there is clear evidence in figure 3F that stress cells exist in vivo, even if there are relatively few of them. It would be interesting to see if the abundance of these stress cells in vitro changes over developmental time and whether they are more abundant at earlier timepoints by using existing earlier data sets (i.e. Eze et al. Nat. Neuro).
9. In the discussion the authors claim that "Our results are compatible with earlier observations that the organoid core, but not surface, is hypoxic". To make this claim the authors should show localization of stress markers near the hypoxic core of the organoid.
10. It is unclear if Gruffi performs better that removing cells based on top stress markers? A comparison of the two methods would be useful.

11. The code and data which are an integral and important part of the manuscript are unavailable ("under reference number GSE1XXXXXXXXX"??) and all novel github links are broken (non-existent or set to private). These have to be made available to be able to determine whether this manuscript can be published.
12. Could these data be applied more broadly outside of brain organoids? Have the authors looked at that in other people's data. That would broaden the appeal of the Method.

Minor issues:
1. In the section titled "Stress is a common hallmark of the two largest unidentified clusters" it is not clear which clusters the authors are referring to. Which are the unidentified clusters? The authors claim that these clusters uniquely express stress markers which is not true as they later show that cell expressing stress markers are present in all clusters (Fig. S2c).
2. "...we analyzed all significantly enriched genes in the stress-clusters". How were significantly enriched genes determined? Are these the differentially expressed genes?
3. "Surprisingly, metabolic terms were both among the strongest and largest enriched terms". How was strength measured?
4. There are many figure references in the text which are incorrect. For example, Figure 1h is referred to as Fig 1f and figure 4e-k are all mixed up in the main text.
5. "Comparing neuronal and glial cell types revealed that all non-dividing glial cells showed higher ER stress scores (Fig 2B)." - figure 2B doesn't show thi point and there was no figure that clearly made this point. Overlaying the stress scores with cell types would be helpful to make this point.
6. "... we did not further adjust the by Gruffi initially estimated thresholds" - the word by should be removed
7. It would be helpful if the authors specified what PROGENy measure in the main text.
8. In figure 3d it would help if the organoid specific populations were clearly labeled on the graph.
9. The authors claim that "Stress genes majorly contribute to variable genes". The data supporting this claim should be shown.
10. The experiment using two media conditions the affect maturation need more details in the text. What is each condition and how does it affect maturation?

Point-by-point response for manuscript EMBOJ-2022-111118

Reviewer comments are shown in black.
Responses are shown in dark blue italics.
Quotes from the revised manuscript are shown in black italics.

Referee #1

Brain organoids are generated from human pluripotent stem cells, and have been used for a number of researches in investigating human brain development and diseases. However, they are artificial structures, and raise concerns in formation of cells of non-physiology. Vertesy et al. reanalyzed the published scRNA-seq from brain organoids as wells as generated their own data, and searched for the artifacts in the brain organoids. They found that there are clusters of cells with signature for stress, including the ER stress, glycolysis, and hypoxia. They developed analytical method, called Gruffi, which remove the stressed cells and showed that after filtering out these cells, the remaining cells better represent the developmental trajectories and mature cell formation. The manuscript deals with the important questions in the brain organoids field, and the analysis seems well performed. However, there are some issues that need addressed.

We thank the reviewer for his/her/their insightful review of our manuscript and the positive assessment. We have optimized the paper substantially based on the reviewer's comments and think that this reviewer helped in improving the paper.

1. When the scRNA-seq data are analyzed, it is critical to perform Q/C to filter out the dead, damaged, or dying cells. Authors present data from filtering cells > 500 genes, < 20% mito, and < 30 rRNA reads. The major portion of the stressed cells are at the category of the cells < 20 % but > 10% of mito reads. The cells with higher mito-reads represent those undergoing apoptosis, and thus including the cells with higher mito-read may have resulted in the stressed cells with GO for response to stress, apoptosis, and glycolysis. Author should present the UMAP with different sets of the filtering to present which are the major factors of stressed cells, and thus in future investigators consider the use of the filters. When the Gruffi is applied to these more stringent conditions, how the stressed cells are distributed, and what are the percentage?

We thank the reviewer for suggesting to look into these relationships. It turned out that some results were quite interesting and it helped to highlight why one needs Gruffi.

The effect of typical quality filters on stressed cell removal

1. Filtering of low gene-count cells.

We explicitly addressed this issue in Fig.EV1B, but now we clarified in Fig. S3 A-C. First of all, gene- and UMI-count per cell is very strongly correlated in all scRNAseq datasets we have seen, therefore either can be used as a filter. We calculated cells retained at 750, 1000, 1250, 1500, 1750, 2000 and 2500 min. genes/cell. We chose to display the results of min. 1000 and 1500 genes/cell, as we saw no surprising trend at higher stringency.

The results can be summarized as follows: (1) Hypoxic/ER−stressed cells cannot be filtered on read counts; (2) low-read count cells are strongly part of the stress clusters as in Fig.1, but they show no enrichment bona fide stressed

cells identified by Gruffi. (3) Increasing stringency leads to cell type depletion. A striking example is that at both 1000 and 1500 genes/cell twice as many DL- than UL-excitatory neurons are removed. The same retaliation holds for midbrain- vs. interneurons.

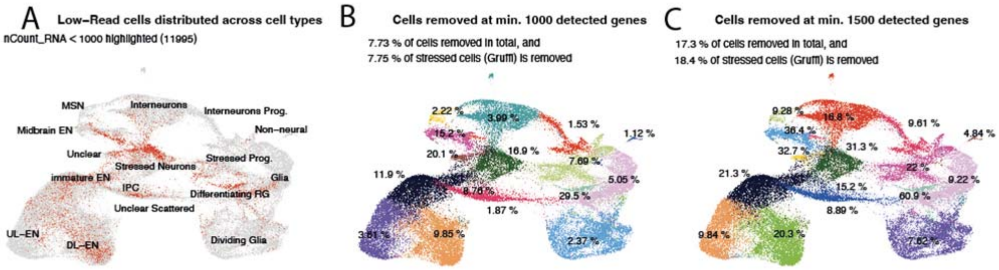

2. Filtering of mito-high cells.

We took the object that has only been filtered for min 500 genes / cell. Here, 0.28 % or 448 of 158,666 of cells have higher than 20% mitochondrial reads, and 1.56 % or 2482 cells have higher than 10%, but less than 20% mitochondrial reads. Both of which thus constitute the minority of the stressed cells. This is so far concordant with observations in Fig. 3A, which shows that stressed cells are actually mito-low.

To see how mito-high cells are distributed, we show both categories, and stressed cells below, and show that they occupy different regions on the UMAP. We also quantified that 0.646 % of stressed cells are above 10% mito. reads, whereas for all cells it is 1.56% (as in main figure, already filtered for 0.28% cells above 20% mito reads.) As a sidenote, comparing to the previous panels, it becomes clear that mito high cells overlap with "low-gene count cells" (S3 A vs F).

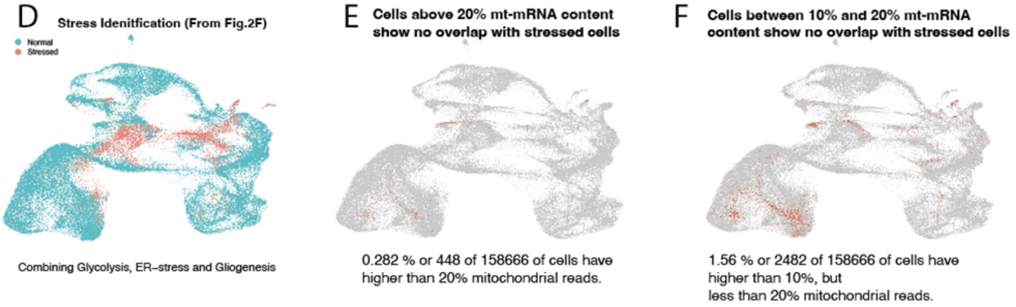

To answer the question how these cells would contribute to a gene signature, if included in a set of cells, we investigated the signatures specific to the 1.85 % or 2932 cells with >10% mito reads, by DGEA. We took genes with log2FC > 0.6, p adj. < 0.01 in mito-high cells (48 coding genes) and analyzed GO-term enrichment in STRING-db. Expectedly, the strongest enrichment was mitochondrial: "Mitochondrial atp synthesis coupled electron transport" (GO:0042775, 5 genes, FDR: 0.012), however a lot more genes were specific to Neurogenesis (GO:0022008, 15 genes, FDR: 0.012), genes largely overlapping with "Cell adhesion". The full results are available at https://version-11-5.string-db.org/cgi/network?networkId=bDQPpScOFDFv.

We found no enrichment related to apoptosis or cell death. ***So, to our surprise it seems that the accepted idea that mito-high cells (>10%) are dying or apoptotic does not appear generally true in brain organoids. Instead, high mitochondrial content is specific to certain cell types (see UMAP) and enrichments suggest that it may reflect the high metabolic needs of matur(ing) neurons.*** *(We did not analyze individual subpopulations, thus there may indeed be apoptotic cells within this population.)*

To confirm *the surprising finding of neural signature, we repeated the analysis on the top 76 coding genes enriched in mito-high cells (with log2FC > 0.5, p adj. < 0.01) and found even more neural,* ***especially neuron migration and synapse organization related terms****. See: https://version-11-5.string-db.org/cgi/network?networkId=bQ7vX89RgpGu*

We then repeated the DGEA analysis for 'extremely high' mitochondrial content. That is 0.28 % or 448 cells with >20% mitochondrial reads, which we excluded in the manuscript. There were only 17 and 18 coding genes above log2FC of 0.6 or 0.5, and it was only enriched in mitochondrial terms (https://version-11-5.string-db.org/cgi/network?networkId=bKoyINVwDSJb and bYSFTWtOwTPL). Therefore, we found no evidence that we should have included the 0.28% mito-high extreme outliers in our analysis, however, generally, ***based on these analyses we advise against filtering mito-high cells in cortical organoids (when one aims to remove dying cells).*** *Of note, removing rare and extreme outliers often make downstream analysis easier, thus researchers may choose to exclude cells they consider extreme.*

3. Filtering of ribo-high cells.

As expected from Fig. 3A, there is an overlap between ribo-high cells and stressed cells. In Fig. S3 G we show that, in the unfiltered object, the 1.94 % cells above 30% ribosomal content occur the strongest in non-telencephalic cells (top right in the umap), supporting the choice of threshold. Next we calculated the fractions of cells above 20% in the filtered dataset presented in the main figures Fig. S3 H-I. We found that these too are most enriched in non-telencephalic cells (50%), but also enriched in stressed progenitors (26%), Unclear (17%), and dividing cells (16%) cells. In conclusion, mis-differentiation in organoids (non-telencephalic cells) seems to be the strongest predictor of high ribosomal content, but ribosomal content between 20-30% is found in multiple unrelated cell types, suggesting distinct reasons for high translational capacity.

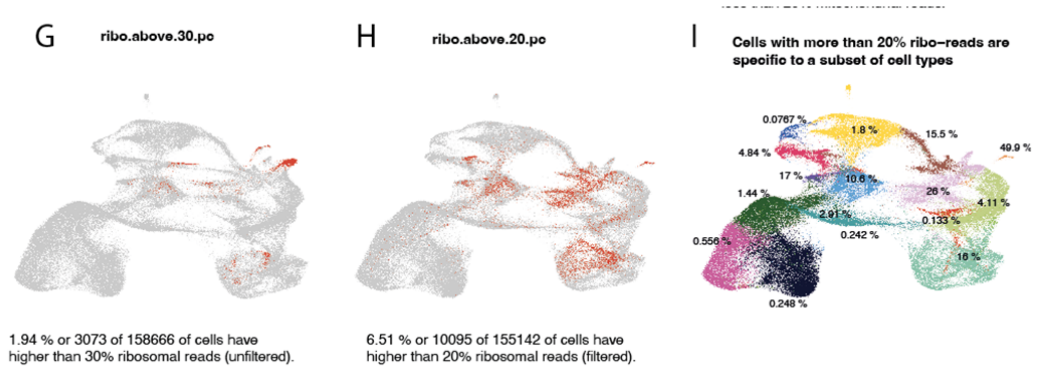

2. In addition, authors filtered out non-neuronal cells to conclude that the stressed cells standout in clusters, and then the stressed cells are not neurons in conclusion. It is contradictory. Authors should present what the unbiased clusters like without filtering out non-neuronal cells, how much overlap the stressed cells make with those filtered non-neuronal cells, and belong to which cell types. Along the same line, the primary brain data also being filtered for the non-neural cells, the authors reach conclusion of the absence of stressed cells. Authors include all cells from the organoids and primary cells of fetal brain data, and show which cells the stressed cells represent.

*We thank the reviewer for his/her/their insightful comment. We regret that the previous version of the manuscript did not sufficiently explain the differences between mis-differentiated cells (that are of non-telencephalic origin) and stressed cells. We have included substantial new analyses to address this important aspect raised by the reviewer (**Appendix Figure S5** and presented here below).*

*During the pre-processing of individual datasets we discovered outlier clusters that were of non-telencephalic origin. To stress the different relationship of these to all other cells they are now called "**non-telencephalic**" instead of "non-neuronal". One such example is presented in Fig. S5, where panel A shows a UMAP with neuronal lineage cells and two outlier clusters. Based on a classifier trained on fetal data (Cao et al., 2020) these cells were most likely mis-differentiated to immune cells and retinal/adrenocortical cells, respectively. This was further supported by a correlation to the fetal reference clusters (B). As UMAP embeddings are non-linear and can distort the distances of cell clusters, we investigated distances in PCA space to further stress the difference between the mis-differentiated non-telencephalic cells and the properly differentiated neuronal lineages (methods, C). This revealed in a heatmap (C, left) as well as in a network graph embedding (C, right visualizing similarity by edge strengths) that the non-telencephalic clusters were not among the neuroectodermal lineages and very distant from our target populations. Thus, we considered them as unwanted mis-differentiation, which motivated the exclusion.*

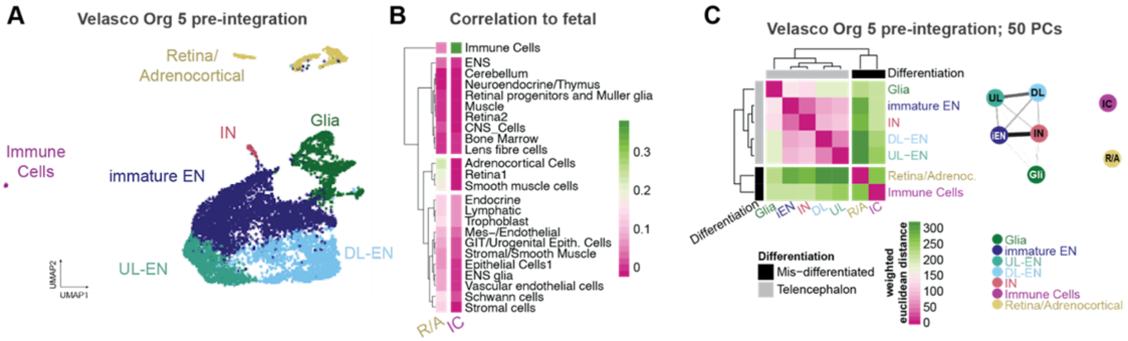

The reviewer suggested integrating all datasets including non-telencephalic cells to investigate the difference of stressed cells unbiasedly. While we agree that it is important to clarify the differences between these cell types, we illustrate below why we think it is not desirable to include such outlier clusters in the integration. scRNAseq data integration as done with canonical-correlation analysis (CCA) relies on defining anchors between datasets. If rare (and unwanted) cell types are included in some but missing from other datasets this can create distortions in the integration (D). After integrating two datasets with both telencephalic plus mis-differentiated cells, and two datasets with pure telencephalic fate (D), we observed that the previously identified non-telencephalic cells were now clustering together with glial cells (E). Strikingly, investigating weighted euclidean distance across the top 50 PCs as done before the integration revealed that all distance between these non-telencephalic cells and glia was lost and they were now very similar to the telencephalic glial progenitors (F). The distortion not only occured between telencephalic and non-telencephalic cells but also among the mis-differentiated clusters: while the two non-telencephalic clusters were very distant from each other before the integration (C) they were among the closest related clusters afterwards (F). This is to be regarded as an integration artifact stemming from the fact that these cells are only in some datasets and in general of low numbers. ***Thus, we concluded that to ensure proper downstream analysis these non-telencephalic cells have to be excluded in advance, which is why we filtered them out before integrating all datasets****.*

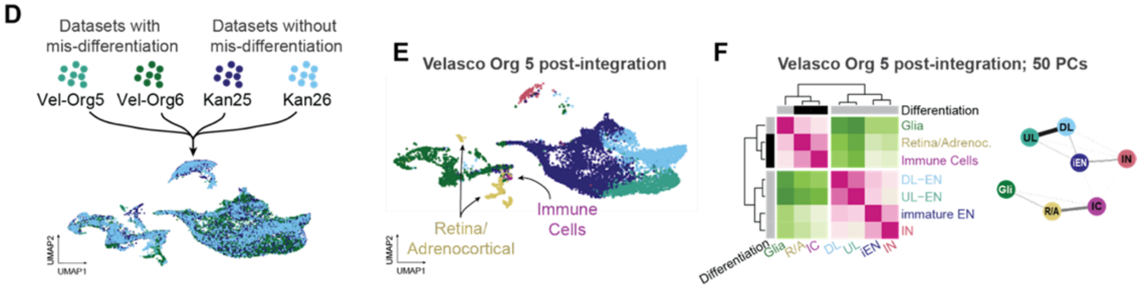

Nevertheless, as we have already identified these non-telencephalic cells in this small scale integration we investigated how they were classified in the stress scoring. GO scoring for glycolysis, ER stress, and negative filtering on gliogenesis revealed that some non-telencephalic cells were classified as "stressed" while others were not (G, R/A and IC in H). Thus, the ***stress***

classification depends on the level of stress programs in these cells and not on telencephalic or non-telencephalic fates alone. *We would like to mention here that these mis-differentiated cell types are most likely not representing properly specified cells as they occur as a tissue culture artifact. Therefore, we think it is not desirable to investigate them further.*

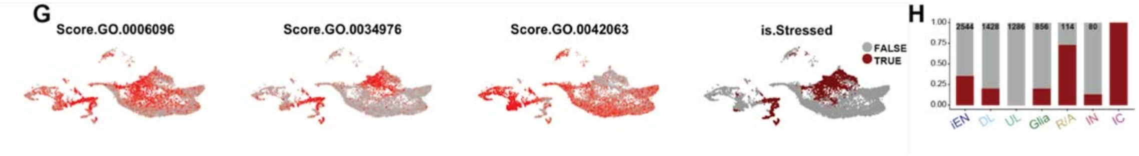

To further illustrate the difference of mis-differentiated and stressed cells we include a scheme in the supplementary figures (I), as well as further analyses demonstrating the improvement after stress filtering (J to L). As shown below on the left side, we argue that mis-differentiated non-telencephalic cells emerge in early stages of the protocol in the embryoid body (EB) and neural induction (NI) phase. In contrast, properly specified telencephalic cells will start to proliferate and generate neuronal lineages that will further differentiate. During this later process stressed cells will arise as an artificial population in the organoid. To further show the differences in their distance and origin we depict the distances of the different populations on the right from the Velasco 5 dataset: while the mis-differentiated cells are very distant from the neuronal lineage, stressed cells are very close to it. Accordingly, to ensure proper downstream analysis the non-telencephalic cells are pre-filtered. While this is easily possible due to their large distance to all other cells, the identification of artificial stressed cells in organoids requires a more nuanced analysis as presented in this work.

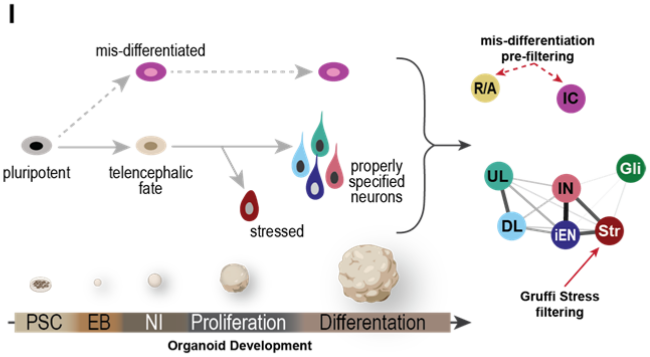

We used this dataset combination to show an example workflow for the analysis as outlined below (Figure 4E). After pre-filtering of mis-differentiated cells (1), datasets are integrated (2). On this integrated dataset of pure telencephalic fate the Gruffi algorithm is used to identify stressed cells (3), and to filter them out (4). At this stage the datasets can be re-integrated (5), which ensures that no stress genes are included in the integration features. Finally, as done in previous panels we used the weighted euclidean distance across the top 50 PCs to compare the stress cleaning improvement (heatmap). We selected differentiated cell types (DL-EN, UL-EN, immature EN, and IN) and calculated the distances between these cell types pre- and post-Gruffi. This revealed that ***after stress exclusion more genes contributing to cell type differences***

***were included**, as evidenced by e.g., a 16.1% increase in distance between IN and immature EN after stress filtering. This corroborates that stress filtering allows to capture more relevant biology from an organoid dataset.*

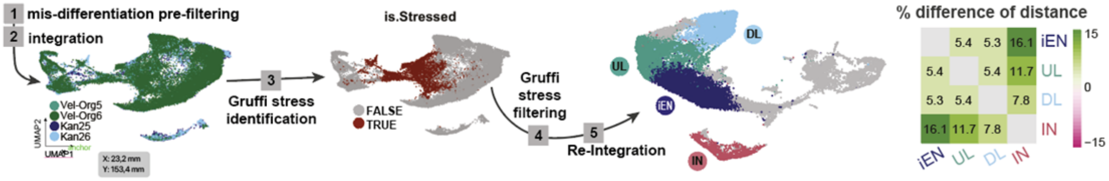

3. One of major question in the paper is "Does stress occur in all or only certain organoid protocols?" But, authors only limited the datasets from the traditional organoids methods. Authors should test whether the stressed clusters are present, or become minimal at the setting where organoids were made with less hypoxic condition. Data from 1) Qian, et al, Cell Stem Cell, 2020, 2) Cakir, et al, Nature Methods, and 3) Giandomenico, et al, Nature Neuroscience, 2019, should be obtained and tested their hypothesis, and use Gruffi to see what are the impact of the methods in these three papers to reduce the stress-induced genes and cells.

We thank the reviewer for his/her/their important suggestion. Great effort in the organoid field has been devoted to optimizing culture conditions. As we stated in the manuscript most solutions that were developed until now require low-throughput slice culture or other methodologies and are therefore unlikely to scale to replace the current standard protocols. Nevertheless, we agree that it is important to test the effect of these optimizations on stress. We have therefore analysed all three datasets in detail and provide the results in Appendix Figure S5 and below.

We downloaded the count matrices for each experiment from GEO and performed standard workflow analysis. UMAP coordinates or metadata annotation was not provided for any of the datasets. We therefore performed the annotation of the clusters ourselves as shown below. Our analysis uncovered that in general neither of the three methodologies was able to abolish the stress identity:

1) Cakir et al.:

The Cakir et al. dataset contains telencephalic cells (RGs and ENs), as well as putative endothelial cells (A). We applied Gruffi to the dataset and found that there were stressed cells identified among the telencephalic lineages (B). This is backed up by expressions of single GO terms (C). Interestingly, the putative endothelial cell clusters that express the GO term 0001935 ("endothelial cell proliferation", also used in their paper) are not classified as stressed and have low expression of stress GO terms, further supporting the specificity of the algorithm beyond brain cells.

2) Giandomenico et al.:

The Giandomenico et al. dataset only contains neuronal lineages (RGs, EN, IN) and was generated on sliced organoids (D). We identified abundant stressed cells next to the normal telencephalic lineage (E).This was further supported by strong expression of especially the glycolysis GO term (GO 0006096, F).

3) Qian et al:

The Qian et al. dataset also only contains neural lineages (RGs, EN), and was generated on repeatedly sliced organoids (G).Also here, we found stressed cells among the organoid cells (H and I).

As these protocols were generated to improve the organoid culture, we decided it is important to independently assess whether the stress-classified cells are indeed stressed. To this end we used the progeny analysis as done previously and found that stressed cells scored highest for hypoxia in all three datasets (J). Thus, Gruffi-identified cells are indeed stressed also in the vasculature-induced and sliced organoid models. Finally, we compared the abundance of stressed cells between the different datasets and found about 6 to 15% of the cells were stressed (K), which is in the range of other datasets (L).

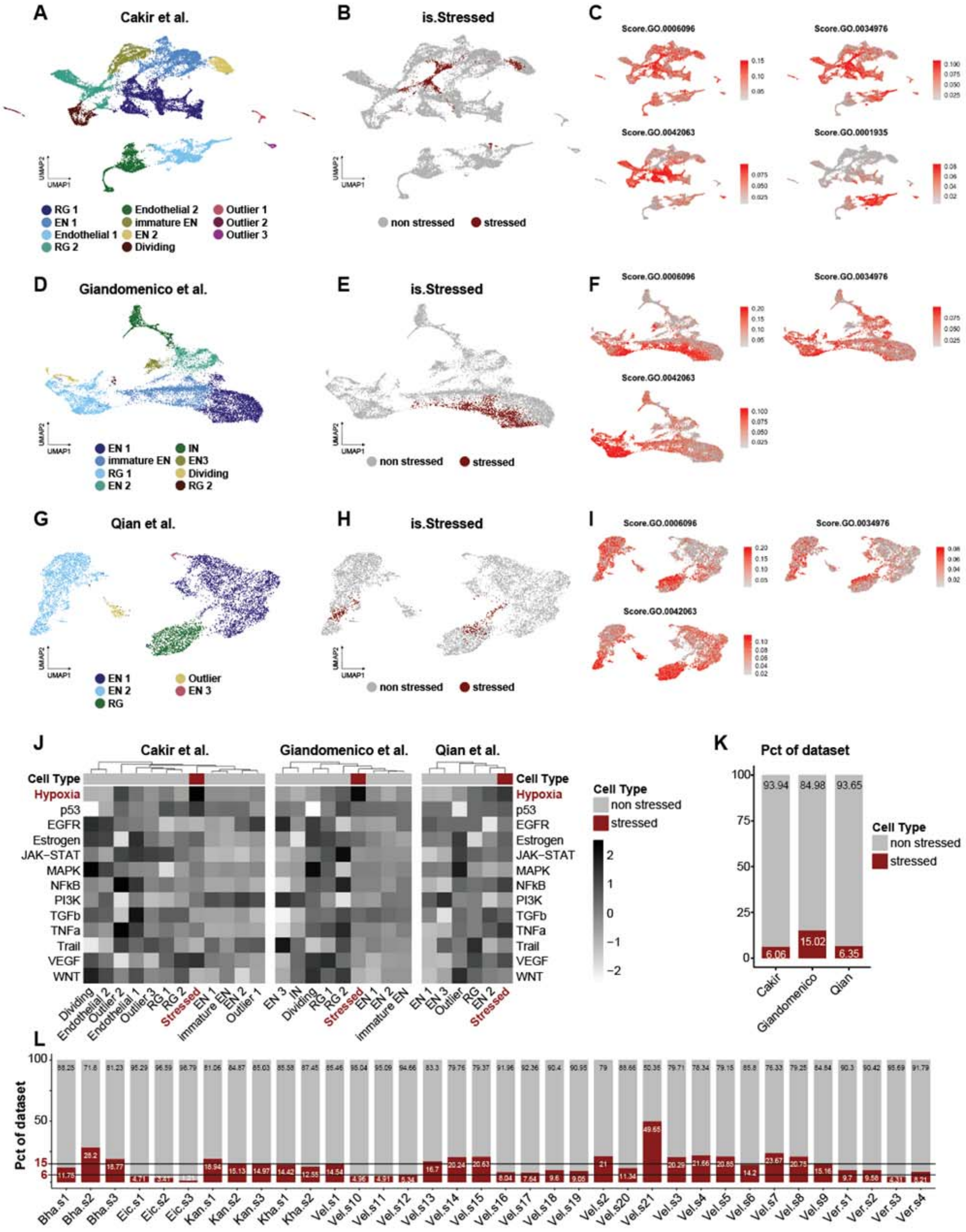

Minor concerns

1. There are a few figures that contain non-relevant contents, like coordinates of x and y, like Figure 3B and 4G. It seems from the cropping of desktop figures. Please replace them.

The figure legends file contained screenshots for faster evaluation. We have now ensured no cropping artefacts are found in any figures.

Referee #2

The manuscript titled "Cellular stress in brain organoids is limited to a distinct and bioinformatically removable subpopulation" by Vertesy et al. identified stress signature in a defined subset of cells in brain organoids. Removing the cells with these stress signatures improved overall data quality and revealed clear developmental trajectories. This manuscript addresses a pressing question in the organoid field and will be of wide impact: whether cellular stress affects organoid development generally and whether these stressed cells affect overall organoid development. This is an open question in the field of brain organoids specifically and the entire organoid field more broadly. Below, I have some suggestions for improvement for the authors.

We thank the reviewer for his/her/their helpful suggestions and the evaluation of our work. We have provided substantial new analyses to incorporate the suggestions of the reviewer and are confident that this improved the quality of our manuscript.

Major issues:

1. The authors claim that the "that stressed cells are in a more 'basal' state compared to differentiating neurons". The authors need to be more exact as to what this 'basal' state is? Do these cells show higher expression of markers for less differentiated cell? Do they show higher expression of stem cell markers?

We thank the reviewer for his/her/their comment. We have clarified the respective sections in the revised version of the manuscript. The investigation of stressed cells revealed high expression of metabolic stress pathways (e.g., ER-stress or glycolysis), while processes like neurogenesis or gliogenesis were reduced (Fig. 2A and Appendix Figure S2A and B). Therefore, we concluded that stressed cells were in a 'stress identity' that was defined by a metabolic survival signature and a lack of differentiation in comparison with correctly specified neurons or glia. "Basal" in this context was meant to illustrate this lack of proper differentiation. We have re-phrased the sections to:

"Compared to both glial and neural clusters, stressed cells also showed remarkably low scores for 'cell differentiation' (GO:0030154), and 'forebrain development' (GO:0030900), suggesting that stressed cells are <u>in a metabolic survival state characterized by a lack of specification to neurons or glia</u> (Appendix Figure S2 A and B). <u>Thus, chronic stress in organoids comes at the expense of neurogenic cell differentiation and leads to an undifferentiated, metabolic and stressed cell-state, which we term as the 'stress identity'."</u>

As for stem-cell markers, we did not find these to be highly expressed in stressed cells.

2. The authors claim there is a switch in cell fate in the stressed cells: "Thus, chronic stress in organoids comes at the expense of neurogenic cell differentiation and leads to a switch in cell fate". However, they don't actually show that the cell fate actually switches as opposed to being less differentiated. A switch in cell fate would show up on the trajectory analysis as a separate trajectory, which is not the case.

We thank the reviewer for raising this. As pointed out by the reviewer, we don't have evidence to distinguish between a terminal cell-fate and a reversible cell-state. We therefore have re-phrased the respective section to refer to cell states instead of fates or trajectories:

" Thus, chronic stress in organoids comes at the expense of neurogenic cell differentiation and leads to an undifferentiated, metabolic and stressed cell-state, which we term as the 'stress identity'."

The full paragraph is included in the response to the previous comment.

3. It is unclear how Granules were constructed and how Gruffi finds and optimal finds an optimal granule resolution. This should be more explicitly discussed in the methods section.

We agree with the reviewer that this was unclear in the manuscript.
What we meant is that the algorithm performs a parameter search (optimization) so that snn-clustering results in a median cluster size between the lower- and upper-thresholds, [100, 200] cells by default. Now we clarified this in the manuscript and in the method sections (changes underlined):

Main text
"We therefore took a grouping approach where we partitioned cells into groups of 100-200 cells by ultra-high-resolution SNN-clustering in PCA-space (Methods) resulting in small groups of cells, that we term granules."

" [...] Gruffi [...] (2). At the same time, it identifies a suitable resolution by parameter search (I), clusters cells into granules (small clusters) (II), and reassigns [...]"

*"[...] After pathway scores calculation, Gruffi identified the clustering resolution where the median cluster size is 154 cells, resulting in 1009 granules (**Fig 2C**)."*

Methods
"Gene detection in single cells is noisy. To overcome this noise, we grouped cells into small clusters (that we call granules) by high-resolution snn-clustering (as in the manuscript, using the algorithm of Seurat). Gruffi's aut.res.clustering(), performs a simple parameter search (for clustering-resolution) that results in a median of 100-200 cells per granule, which we call the optimal resolution, as clusters contain a statistically robust number of cells. All these parameters are also manually customizable."

4. In various parts of the manuscript the authors rely on UMAP distance to make claims about the difference between cells (including in how they merge granules). This is certainly better than tSNE, but not entirely correct, as UMAP is a method that distorts the underlying data/distances making the distances somewhat arbitrary as discussed

here: https://www.biorxiv.org/content/10.1101/2021.08.25.457696v3

We thank the reviewer for his/her/their comment raising an important point. While often overlooked, non-linear d.r. methods like UMAP or t-SNE can result in distortion of distances.

As we found this an interesting question (Are differences seen in 2D UMAPs supported by differences in higher dimensions?), we have added substantial new analysis to illustrate cell type differences. We are grateful for this point, because now we have substantial evidence to show that Gruffi leads to better cell type separation, regardless whether distance is calculated in gene expression, PCA or UMAP space.

To this end, we calculated euclidean distance across principle components (PCA, weighted by variance explained, Appendix Figure S5 and Fig. 4E), which are therefore linear distances in high dimensional space. We illustrate that exclusion of stressed cells results in capturing more biological meaningful difference, as evidenced by larger distance between differentiated cell types (Fig. 4E, Appendix Figure S5). We have changed all respective passages of the text to not use distance of UMAP space for comparing cell differences.

To clarify, our algorithm for calculating granules is based on the knn graph clustering, which in turn is based on PCA. Thus, granule construction itself is irrespective of UMAP space. As the reviewer correctly pointed out, the reclassification of small granules is based on 3D UMAP space, as we identified this as most efficient for this problem. In light of the above conclusion (distances and trends observed in UMAP space hold through in higher dimensions), we feel it is justified to keep Gruffi's code unchanged.

As a side note, we think that even with their discussed/referenced limitations, UMAP and other d.r. methods are tremendously useful to visualize high-dimensional data in a simple and interpretable way, they are successful in denoising the data, and in capturing relevant differences.

5. The authors state that the single cell approach is suitable for identifying correctly specified cells with high levels of stress. How do the authors explain the high levels of stress in these cells? What is causing these cells stress and why are they less differentiated than the other cells in the cluster? Also, can the authors explain why are the scSC are not more abundant (enriched?) in the stress clusters. Could it be that these are not really stressed, but just different maturation trajectories?

We thank the reviewer for raising these important points and answer them one-by-one below:

How do the authors explain the high levels of stress in these cells?

We agree with the reviewer, that this is an interesting point. To reiterate: our observation is that when selecting cells on high stress-expression, (by definition) these cells share the "high expression of stress-genes" with bona-fide (Gruffi classified) stressed cells. Yet, they limitedly share the other features, such as

the lack of differentiated cell fate, low mitochondrial, or high ribosomal read fraction (Fig S2D-I). This result can be explained by multiple reasons:

(1) Selection bias: if we select on X, we will see high X; (2) Noise in single-cell gene expression data: these (scSC) cells indeed seem to be similarly distributed across cell types (Fig S2C “only cellwise”). We also quantified this (stressed cells per cluster, by either method), and scSC showed a median of 7% stressed per cluster, with a CV of 0.4, whereas, gSC showed a median of 2% stressed per cluster, with a CV of 1.5, indicating that scSC ‘finds’ cells to be stressed in any cell type with similar probability, that is unlike gSC (see below).

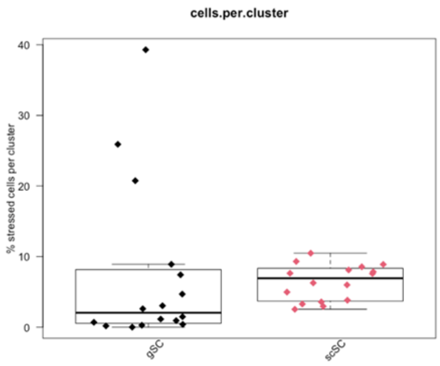

(3) We hypothesized that one plausible explanation for the difference between “stressed identity” cells (that we want to exclude) and these cells may be that the latter experience stress for a shorter time (acutely) or to a lesser degree, that does not trigger a change in cellular identity. Our data however does not permit us to delineate the origin and specific feature of these cells in greater detail.

What is causing these cells stress and why are they less differentiated than the other cells in the cluster?

This point refers to (Fig S2G). As discussed in (3) above, these cells may experience the same stressors less or for a shorter time. As we showed that hypoxic & ER stress leads to less differentiation in gSC, we would expect that on average, we see a similar trend in scSC. Overall, these cells are more stressed than their counterparts in the same cluster, thus the expectation would be that, on average, they show less differentiation, as seen in (Fig S2G).

Also, can the authors explain why are the scSC are not more abundant (enriched?) in the stress clusters.

This question refers to (Fig S2C), and the answer is quite simple: the naive approach of selecting the highest stress expressing cells (scSC) is limited to genes within 2 pathways (the 3rd is a negative score), and ignores all other transcriptome wide features in these cells. Gruffi however takes that into account by first grouping cells by transcriptome-wide similarities, thus the higher overlap with the stress-cluster, identified by transcriptome-wide clustering as well.

Could it be that these are not really stressed, but just different maturation trajectories?

As the scSC cells are scattered throughout all clusters, they have little in common, besides stress gene expression. We therefore do not see any indication that this `class` of scSC cells would constitute a separate trajectory. (We have to admit that we are not sure if we understood this question)

6. In Figure 3A are changes significant across the autophagy/ubiquitin pathways? What comparisons were made?

We now do a more correct statistical analysis, and added the description to the following section to the figure legend:

"All differences were significant on Kruskal-Wallis test (p=0) followed up by a Bonferroni corrected Dunn's test (p.adj<1e-100 for all, except %ribo Prog. vs. Str. Neurons: p.adj<5e-7). Group averages and relative changes quantified in Table S5."

For consistency, as in the original version, we also calculated the MWW tests for the missing pathways mentioned by the reviewer, and they were p<2.2e-16 in all cases. We note that in single-cell analyses, because of the very large number of observations, almost all differences are significant.

7. In the section describing figure 3b hypoxia is only enriched in Gruffi identified cells and not in the naïve clusters. Could the authors comment on why these naïve clusters are not enriched for hypoxia as they contain appear to highly overlap with the Gruffi clusters. One concern is that as Gruffi uses similar stress terms to define the cells there may be confirmation bias that the authors and defining and measuring the Gruffi results based on the same or similar genes.

We thank the reviewer for his/her/their comment. We agree that it is important to validate our findings by various different methods. Besides using GO-terms, that of course have an overlap with the GO-terms used to define the stressed cells, we additionally employed several other methods. In Fig. 3A we investigate mitochondrial and ribosomal reads that are irrespective of the stress GO-terms. Furthermore, we use PROGENy to investigate pathway activity in the different clusters. PROGENy derives pathway responsive genes from a large compendium of perturbation experiments, and thus is not reliant on GO-terms. In the set of pathways analyzed by PROGENy, two pathways that we did not use for Gruffi, showed stressed-cell specific activity: the hypoxia- and VEGFA-pathways.

We now improved the section on PROGENy (Fig. 3B) in the text to better explain the comparisons that were done. Fig. 3B investigates different pathways across all clusters, while separating the stress clusters into 2 groups: Either identified by Gruffi, or cells included in the stress cluster but not identified by Gruffi (=Clustering only). This revealed that while Gruffi identified cells had

enrichment in hypoxia and VEGFA pathway, cells that were in stressed clusters but not identified by Gruffi did not. The text was changed to:

"To ask how stressed cells identified by Gruffi differ from those detected by the naïve clustering-based approach (Figure 1), we subcategorized the stressed clusters into cells identified by Gruffi or cells in the stressed clusters but not classified by Gruffi. The complete lack of separation of cells found by only the naïve approach contrasted the salient stress features found by Gruffi (Figure 3B). This suggests that a clustering-based approach is impractical for excluding stressed cells as it is variable between datasets and would exclude non-stressed cells, further supporting the versatility of Gruffi."

Additionally, we used the comparison to fetal cells to illustrate that the stressed state is highly enriched and artificial in organoids.

8. While the authors claim the section title that "Stressed cells in organoids have no fetal counterpart" there is clear evidence in figure 3F that stress cells exist in vivo, even if there are relatively few of them. It would be interesting to see if the abundance of these stress cells in vitro changes over developmental time and whether they are more abundant at earlier timepoints by using existing earlier data sets (i.e. Eze et al. Nat. Neuro).

We thank the reviewer for prompting us to look into other time points of fetal development, and to clarify this point.

The presence of stress in the human fetal brain is widely debated, especially in relation to stress in organoids. The reviewer is correct that Fig. 3F shows that some individual cells are classified as stressed in the fetal dataset. We would like to draw attention to the following points:

(1) The number of stressed cells in fetal samples is very low, and of course our algorithm is not perfect, which can lead to some misclassification. (2) More importantly, granules were calculated together for fetal and organoid cells. If we investigate the underlying cells, it becomes evident that stress-classified cells in fetal show much lower "Hypoxia" activation than in organoids (Fig. S4F). This suggests that stress in organoids is different from the stress found in the fetal brain.

We nevertheless agree that it is important to test whether a stress identity is present in the human brain, especially in earlier stages that include more radial glia cells. We therefore investigated the mentioned Eze et. al dataset and include this in the revised Figure EV2 and below here.

Panel A shows clustering of the fetal early progenitors colored by Carnegie stage using the coordinates provided by the authors. We applied the Gruffi algorithm to the dataset and found that no stressed cells were identified (B). To exclude that this result is due to technical inconsistencies like the sequencing depth, we compared the genes of each GO-term that were found in the fetal and the organoid dataset (C). For all GO-terms the number of detected genes was similar and largely overlapping, excluding a technical problem. We also investigated expression of each score (D) and found that there was not one clear cluster with increased stress scores. Notably, the scores for stress GO-

terms were low in fetal as revealed by comparing the granule-wise scores of fetal and organoid data (E). We plotted the glycolysis and ER stress scores against each other and colored each granule by the stress annotation. Each point reflects one granule and its size is relative to the expression of the glycolysis score. Only in the organoid granules with very high stress scores are found, that were then classified as stressed. Individual granules had high gliogenesis scores (large size) and were thus not classified).*

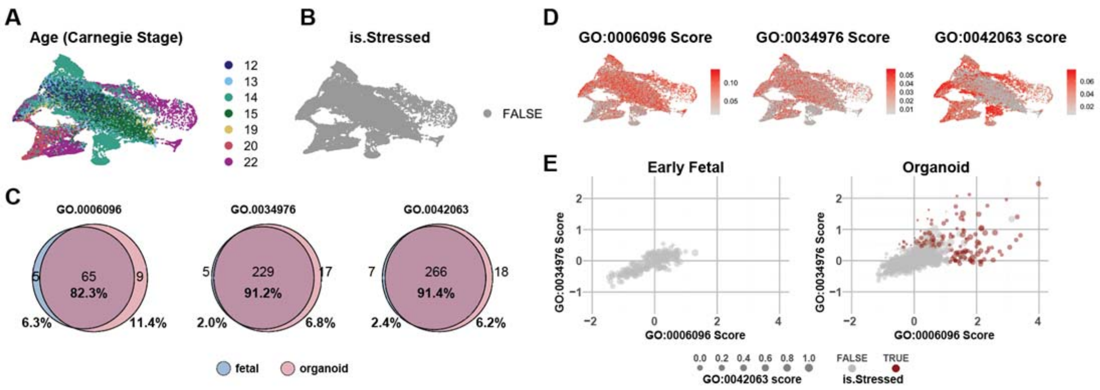

9. In the discussion the authors claim that "Our results are compatible with earlier observations that the organoid core, but not surface, is hypoxic". To make this claim the authors should show localization of stress markers near the hypoxic core of the organoid.

We thank the reviewer for his/her/their suggestion. The presence of hypoxic conditions in the center of an organoid has been reported numerous times before. We agree that it is important to link this to the metabolic changes that we find. A preprint that uses spatial transcriptomics to investigate the distribution of different transcriptomic signatures was recently uploaded to bioRxiv (Uzquiano et al., 2022). We have added this citation to the manuscript:

“Our results are compatible with earlier observations that the organoid core, but not surface, is hypoxic (Qian et al., 2019), explaining why stress characterizes only in a defined set of cells. This is further supported by a recent pre-print employing spatial transcriptomics to identify hypoxic metabolic programs in the organoid core (Uzquiano et al., 2022).”

10. It is unclear if Gruffi performs better that removing cells based on top stress markers? A comparison of the two methods would be useful.

We thank the reviewer for his/her/their suggestion. We tried to address this question with the comparison of single-cell scoring (scSC) and granular scoring (gSC) in the previous version of the manuscript. In this comparison we investigated three groups of cells: the intersection of scSC and gSC, as well as scSC-only and gSC-only cells. This revealed that both the intersection and gSC-

only classified cells showed a stress-identity, while scSC-only cells did not. Nevertheless, we include both methods in the Gruffi algorithm.

We have expanded the section on this comparison:

"To test the granular approach, we compared stressed cells identified by Gruffi's granular method (gSC) and stressed cells identified on single-cell scores (scSC). We contrasted cells only identified by either, both or neither of the approaches, thus validating whether top stress markers alone is superior in filtering stressed cells. First of all, scSC were evenly scattered across all clusters, while most gSC were close to the previously identified stress clusters and the cells identified by both methods (Appendix Figure S2C). By definition, single-cell selection on stress scores identified the cells with highest stress-scores. Thus, we tested whether the scSC-only selected cells had other hallmarks of stressed cells. scSC cells showed less of all other features defining stress-identity: lack of cell differentiation, lower mitochondrial and higher ribosomal mRNA content. In contrast, granular identification found cells that shared these features with cells identified by both methods and showed strong stress-identity (Appendix Figure S2D-I). Thus, single-cell scoring based on top stress markers alone is not sufficient to identify stressed cells. While the intersection of both scSC and gSC classification showed strong stress-identity, this was also the case for gSC-only cells. We implemented both methods in Gruffi, but we concluded that the granular approach is more suitable if one aims to exclude stress-identity, whereas the single cell approach is more suitable if one aims to simply find cells with the highest stress gene expression, but otherwise properly specified cells."

11. The code and data which are an integral and important part of the manuscript are unavailable ("under reference number GSE1XXXXXXXXX"??) and all novel github links are broken (non-existent or set to private). These have to be made available to be able to determine whether this manuscript can be published.

We are in the process of uploading to GEO. We communicate this to the editor and will provide the ID as soon as we obtain it, before publication. We are happy to provide the raw data to reviewers until then.

12. Could these data be applied more broadly outside of brain organoids? Have the authors looked at that in other people's data. That would broaden the appeal of the Method.

We thank the reviewer for this interesting suggestion. We have focused on the brain organoid system as these are among the largest and most diverse organoid cultures. We agree that it would be exciting to test Gruffi on other datasets. To this end, we have used a published resource of retinal organoid cultures that include comparisons to fetal data (Sridhar et al., 2020). We focused on the d205 organoid dataset that the authors compared to central and peripheral retina at d125 (Figure 6 in (Sridhar et al., 2020)). The results are reported in Figure EV6 and reported here below.

We accessed raw count matrices and processed the data with cell type annotations provided by the authors. After CCA alignment and dimensionality

reduction several cell types as defined by the authors can be found. Notably, a few cell types are strongly biased towards organoid cells (A to C):

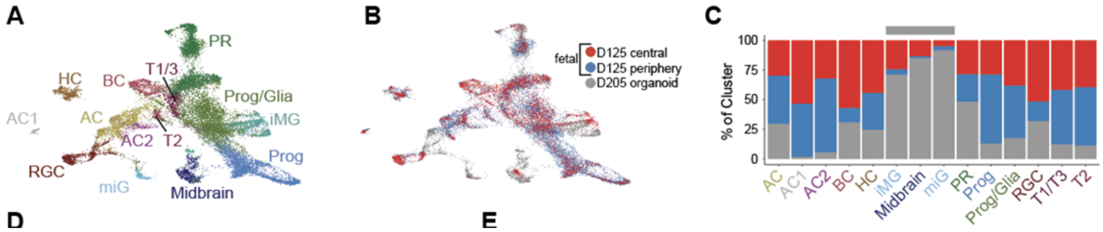

We found that RGCs (retinal ganglion cells) show a strong glycolytic signature, characteristic for this cell type. Thus, we generated a negative score for RGCs (including the genes: ISL1, SNCG, RBPMS) and applied scWGCNA to identify an additional stress score. This revealed cellular response to hypoxia as strongly enriched in organoid cells (D to G):

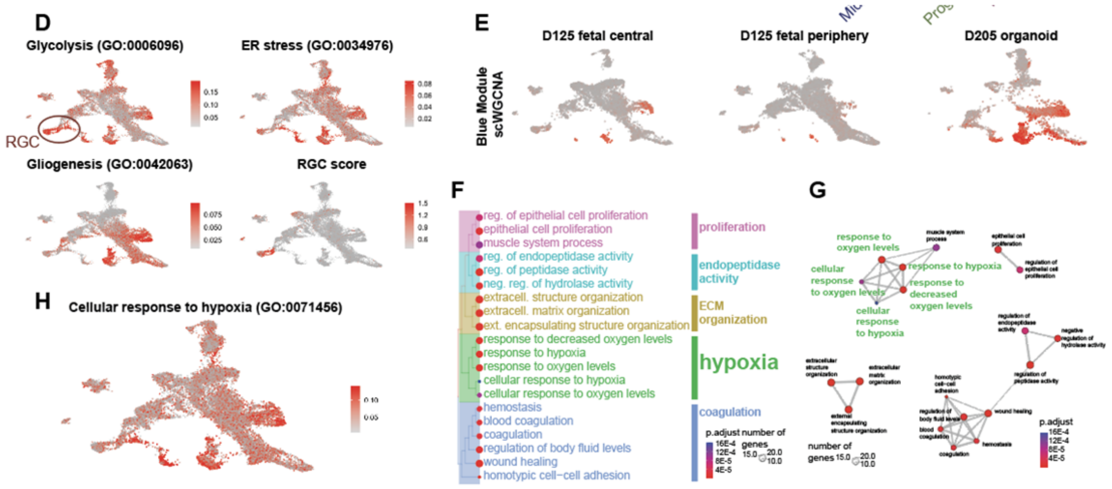

We applied these scores to the integrated dataset and identified stressed cells in retinal organoids, while they were rare in fetal datasets. Notably, clusters that were strongly biased towards organoid cells were mostly stressed (I and J):

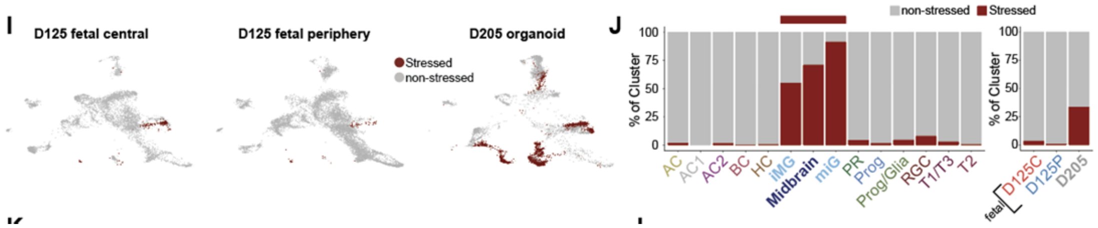

In Figure EV6 we provide additional analyses like per granule stress scores for all datasets, PROGENy pathway scores, and re-integration/clustering of the data after stress filtering. This analysis demonstrated that the Gruffi workflow can be easily applied to other datasets, however, it might require careful investigation of the underlying metabolic pathways and selection of proper stress scores.

Minor issues:

1. In the section titled "Stress is a common hallmark of the two largest unidentified clusters" it is not clear which clusters the authors are referring to. Which are the unidentified clusters? The authors claim that these clusters uniquely express stress markers which is not true as they later show that cell expressing stress markers are present in all clusters (Fig. S2c).

We have updated the respective sections in the text:

“Differential gene expression and QC-metric analysis revealed that the ‘unidentified’ <u>two</u> central clusters (<u>Stressed Neurons and Stressed Progenitors, Figure 1B)</u> consisted of cells <u>strongly</u> expressing stress markers and low-quality cells […]”

2. "...we analyzed all significantly enriched genes in the stress-clusters". How were significantly enriched genes determined? Are these the differentially expressed genes?

Yes, these are differentially expressed genes. We now added a reference to the methods with exact description of this procedure and rephrased the main text as:

“To better understand the nature of stress in these cells, we analyzed all significantly differentially enriched genes in the stress-clusters (methods).”

In methods:

Differential Gene Expression Analysis (DGEA)

“We used the Wilcoxon test via the FindAllMarkers() and FindMarkers() to identify differentially expressed genes, with the folllowing parameters: return.thresh = 0.001; min.pct = 0.1; min.diff.pct = 0.01; min.cells.group = 100; min.cells.feature = 100; logfc.threshold = 0.25.”

3. "Surprisingly, metabolic terms were both among the strongest and largest enriched terms". How was strength measured?

We agree with the reviewer that this section needed clarification. These results refer to GO-term enrichment analysis that is now described in the methods as:

GO-term enrichment

GO-term enrichment was performed on coding, differentially expressed genes, using the STRING toolkit (Szklarczyk et al. 2019). Enriched terms were filtered by False Discovery Rate (FDR), so that the Benjamini–Hochberg multiple testing corrected p-values <0.05. ***Next, terms were ranked by Enrichment Strength, that is the Log10(observed / expected) codung gene count. Where "expected" is the number of proteins with a given annotation that is expected in a random gene set of the same size.***

In the main text, now we refer to the methods section, which we hope answers the reviewer precisely.

4. There are many figure references in the text which are incorrect. For example, Figure 1h is referred to as Fig 1f and figure 4e-k are all mixed up in the main text.

We corrected these mistakes in the new version of the manuscript.

5. "Comparing neuronal and glial cell types revealed that all non-dividing glial cells showed higher ER stress scores (Fig 2B)." - figure 2B doesn't show this point and there was no figure that clearly made this point. Overlaying the stress scores with cell types would be helpful to make this point.

We agree with the reviewer that this could be better quantified by showing average scores per cell classes, so we calculated the average scores per cluster, where glia score third highest after stress clusters:

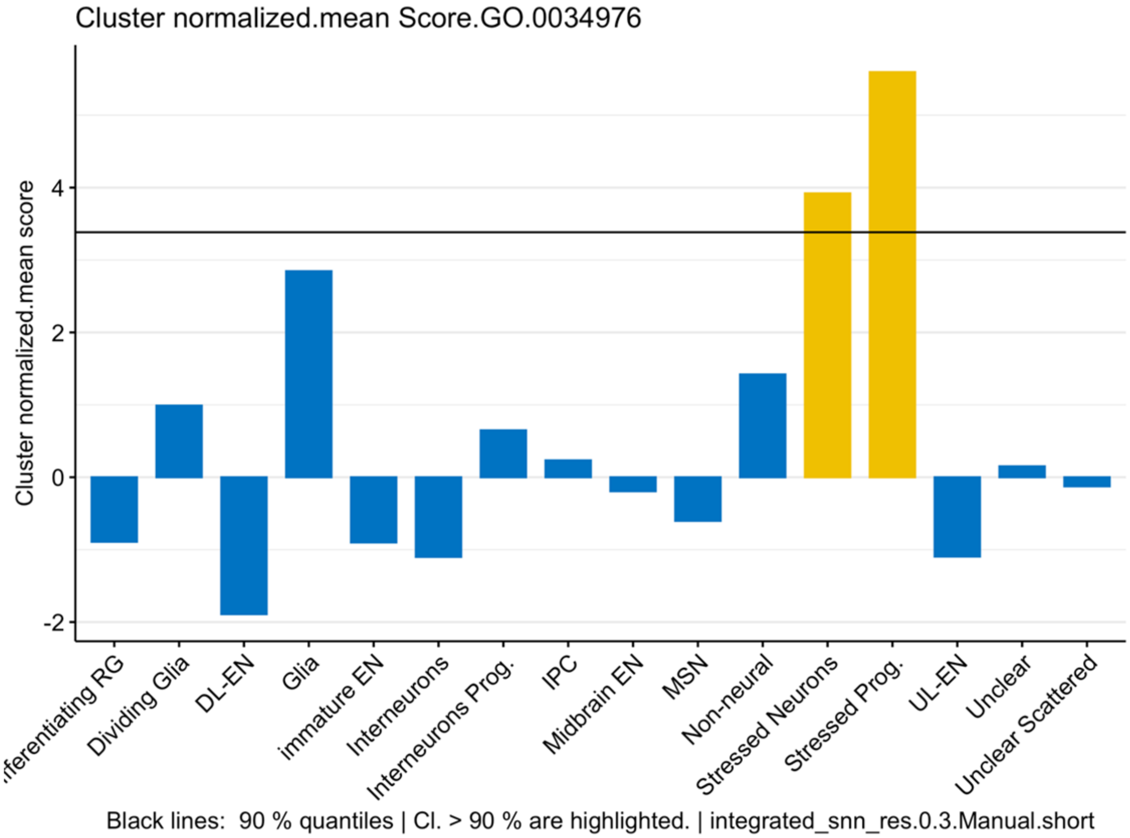

6. "... we did not further adjust the by Gruffi initially estimated thresholds" - the word by should be removed

We thank the reviewer for finding this mistake. We corrected the sentence as follows:

"Throughout our analysis presented here, we did not adjust manually the thresholds estimated by Gruffi"

7. It would be helpful if the authors specified what PROGENy measure in the main text.

We now include an explanation in the main text:

"As a complementary approach to GO-term defined pathway scores, we turned to PROGENy, which derives pathway responsive genes from a large compendium of perturbation experiments, and assigns a Z-score normalized

activity to each pathway (Schubert et al., 2018). Therein, we analyzed cell types by clustering PROGENy's activity-scores across signaling pathways and clusters."

8. In figure 3d it would help if the organoid specific populations were clearly labeled on the graph.

We agree with the reviewer that the organoid specific populations should be clearly labeled. We now highlighted the two organoid specific populations with red arrows and added to the legend "Red arrows indicate groups of cells only / mostly present in the organoid samples."

9. The authors claim that "Stress genes majorly contribute to variable genes". The data supporting this claim should be shown.

We agree that this statement lacked support. We now added:

"In the organoid integration dataset 22 from the top 50, and 36 from the top 100 variable genes were enriched in "Response to stress" (GO:0006950; FDR: 3.4e-3 & 2e-3)."

10. The experiment using two media conditions the affect maturation need more details in the text. What is each condition and how does it affect maturation?

We have added the following sections to the manuscript:

*"While the original organoid medium contains high concentrations of nutrients and amino acids and thus supports progenitor expansion it is less suitable for neuronal maturation. A low-nutrient media composition, in contrast, based on a defined 2D neuron culture medium (BrainPhys, Bardy et al. 2015) more closely resembles the cortical environment and enabled further maturation of neurons. To test whether stress affects maturation, we grew pairwise matched samples in the two different media conditions and analyzed them together (**Fig 4G**)."*

References:

Bardy C, Hurk M van den, Eames T, Marchand C, Hernandez RV, Kellogg M, Gorris M, Galet B, Palomares V, Brown J, et al (2015) Neuronal medium that supports basic synaptic functions and activity of human neurons in vitro. Proc National Acad Sci 112: E3312

Cakir B, Xiang Y, Tanaka Y, Kural MH, Parent M, Kang Y-J, Chapeton K, Patterson B, Yuan Y, He C-S, et al (2019) Engineering of human brain organoids with a functional vascular-like system. Nat Methods 16: 1169–1175

Cao J, O'Day DR, Pliner HA, Kingsley PD, Deng M, Daza RM, Zager MA, Aldinger KA, Blecher-Gonen R, Zhang F, et al (2020) A human cell atlas of fetal gene expression. Science 370: eaba7721

Eze UC, Bhaduri A, Haeussler M, Nowakowski TJ & Kriegstein AR (2021) Single-cell atlas of early human brain development highlights heterogeneity of human neuroepithelial cells and early radial glia. Nat Neurosci 24: 584–594

Giandomenico SL, Mierau SB, Gibbons GM, Wenger LMD, Masullo L, Sit T, Sutcliffe M, Boulanger J, Tripodi M, Derivery E, et al (2019) Cerebral organoids at the air-liquid interface generate diverse nerve tracts with functional output. Nat Neurosci 22: 669–679

Qian X, Su Y, Adam CD, Deutschmann AU, Pather SR, Goldberg EM, Su K, Li S, Lu L, Jacob F, et al (2020) Sliced Human Cortical Organoids for Modeling Distinct Cortical Layer Formation. Cell Stem Cell 26: 766-781.e9

Sridhar A, Hoshino A, Finkbeiner CR, Chitsazan A, Dai L, Haugan AK, Eschenbacher KM, Jackson DL, Trapnell C, Bermingham-McDonogh O, et al (2020) Single-Cell Transcriptomic Comparison of Human Fetal Retina, hPSC-Derived Retinal Organoids, and Long-Term Retinal Cultures. Cell Reports 30: 1644-1659.e4

Uzqiano, A., Kedaigle, A.J., Pigoni, M., Paulsen, B., Adiconis, A., Kim, K., Faits, T., Nagaraja, S., Antón-Bolaños, N., Gerhardinger C., et al (2022) Single-cell multiomics atlas of organoid development uncovers longitudinal molecular programs of cellular diversification of the human cerebral cortex. 2022.03.17.484798

1st Revision - Editorial Decision 27th Jun 2022

Dear Jürgen,

Thank you for submitting your revised MS to The EMBO Journal. Your study has now been seen by referee #1 who appreciates the introduced changes and supports publication here. The referee has no further comments to the authors.

Given this I am pleased to accept the MS for publication here. However
before sending you the formal acceptance letter there are some formatting issues that need to be sorted out:

- The accession code for the deposited data should to the data availability section.

- The ms is split into 3 word files (main text, methods and figure legends). Please combine them into one word file.

- we are missing 3-5 keywords

- COI should be updated to Disclosure & Competing Interests Statement - see also our guide to authors

- Please check the reference format. References with more that 10 authors listed should be cut after 10 et al.

- Make sure that the funding information is also provided in the online submission platform.

- Please upload main figures and EV figures as individual figure files.

- Callouts are missing for Figures 1D, 2E, 3F, EV6H, EV6N, Appendix Fig. S4C callouts are missing. There is a text callout to EV3E, but no such panel.

- There are callouts to Table S7, which doesn't exist.

- Tables S1, S4 + S5 should be Dataset EV files (for dataset tables please add a legend as a separate tab in the excel file). Table S3 + S6 can be either Dataset or Appendix tables. Table S2 should be an Appendix or EV table. If you make them into appendix tables, then please include them in the Appendix. Please note the following nomenclature: Table EV1, Dataset EV1, Appendix Table S1. See also guide to authors https://www.embopress.org/page/journal/14602075/authorguide#tablesformat

- The Appendix needs a ToC

- We encourage authors to include datasets obtained from public resources in the reference list in the form of data citations. See also guide to authors under Data Citation. https://www.embopress.org/page/journal/14602075/authorguide#referencesformat

- We include a synopsis of the paper (see http://emboj.embopress.org/). Please provide me with a general summary statement and 3-5 bullet points that capture the key findings of the paper.

- We also need a summary figure for the synopsis. The size should be 550 wide by [200-400] high (pixels). You can also use something from the figures if that is easier.

- Our publisher has also done their pre-publication check on your manuscript. When you log into the manuscript submission system you will see the file "Data Edited Manuscript file". Please look at the word file and the comments regarding the figure legends and respond to the issues. There are just a few minor edits that need to be incorporated into the final version.

That should be all. Let me know if you have any questions regarding the formatting issues. We are happy to help out

With best wishes

Karin

Karin Dumstrei, PhD
Senior Editor
The EMBO Journal

Use the link below to submit your revision:

https://emboj.msubmit.net/cgi-bin/main.plex

2nd Revision - Editorial Decision 4th Jul 2022

Dear Jürgen,

Thank you for submitting your revised manuscript. I have now had a chance to take a careful look at everything and all looks good.

I am therefore very pleased to accept the MS for publication here.

With best wishes

Karin

Karin Dumstrei, PhD
Senior Editor
The EMBO Journal

Please note that it is EMBO Journal policy for the transcript of the editorial process (containing referee reports and your response letter) to be published as an online supplement to each paper. If you do NOT want this, you will need to inform the Editorial Office via email immediately. More information is available here:
https://www.embopress.org/page/journal/14602075/authorguide#transparentprocess

Your manuscript will be processed for publication in the journal by EMBO Press. Manuscripts in the PDF and electronic editions of The EMBO Journal will be copy edited, and you will be provided with page proofs prior to publication. Please note that supplementary information is not included in the proofs.

You will be contacted by Wiley Author Services to complete licensing and payment information. The required 'Page Charges Authorization Form' is available here: https://www.embopress.org/pb-assets/embo-site/tej_apc.pdf - please download and complete the form and return to embopressproduction@wiley.com

Should you be planning a Press Release on your article, please get in contact with embojournal@wiley.com as early as possible, in order to coordinate publication and release dates.

If you have any questions, please do not hesitate to call or email the Editorial Office. Thank you for your contribution to The EMBO Journal.

** Click here to be directed to your login page: https://emboj.msubmit.net

EMBO Press Author Checklist

Corresponding Author Name: Juergen A Knoblich	
Journal Submitted to: EMBO Journal	
Manuscript Number: EMBOJ-2022-111118	

USEFUL LINKS FOR COMPLETING THIS FORM
The EMBO Journal - Author Guidelines
EMBO Reports - Author Guidelines
Molecular Systems Biology - Author Guidelines
EMBO Molecular Medicine - Author Guidelines

Reporting Checklist for Life Science Articles (updated January 2022)
This checklist is adapted from Materials Design Analysis Reporting (MDAR) Checklist for Authors. MDAR establishes a minimum set of requirements in transparent reporting in the life sciences (see Statement of Task: 10.31222/osf.io/9sm4x). Please follow the journal's guidelines in preparing your manuscript.
Please note that a copy of this checklist will be published alongside your article.

Abridged guidelines for figures
1. Data
The data shown in figures should satisfy the following conditions:
- the data were obtained and processed according to the field's best practice and are presented to reflect the results of the experiments in an accurate and unbiased manner.
- ideally, figure panels should include only measurements that are directly comparable to each other and obtained with the same assay.
- plots include clearly labeled error bars for independent experiments and sample sizes. Unless justified, error bars should not be shown for technical replicates.
- if n<5, the individual data points from each experiment should be plotted. Any statistical test employed should be justified.
- Source Data should be included to report the data underlying figures according to the guidelines set out in the authorship guidelines on Data Presentation.

2. Captions
Each figure caption should contain the following information, for each panel where they are relevant:
- a specification of the experimental system investigated (eg cell line, species name).
- the assay(s) and method(s) used to carry out the reported observations and measurements.
- an explicit mention of the biological and chemical entity(ies) that are being measured.
- an explicit mention of the biological and chemical entity(ies) that are altered/varied/perturbed in a controlled manner.
- the exact sample size (n) for each experimental group/condition, given as a number, not a range;
- a description of the sample collection allowing the reader to understand whether the samples represent technical or biological replicates (including how many animals, litters, cultures, etc.).
- a statement of how many times the experiment shown was independently replicated in the laboratory.
- definitions of statistical methods and measures:
  - common tests, such as t-test (please specify whether paired vs. unpaired), simple χ2 tests, Wilcoxon and Mann-Whitney tests, can be unambiguously identified by name only, but more complex techniques should be described in the methods section;
  - are tests one-sided or two-sided?
  - are there adjustments for multiple comparisons?
  - exact statistical test results, e.g., P values = x but not P values < x;
  - definition of 'center values' as median or average;
  - definition of error bars as s.d. or s.e.m.

Please complete ALL of the questions below.
Select "Not Applicable" only when the requested information is not relevant for your study.

Materials

Newly Created Materials	Information included in the manuscript?	In which section is the information available? (Reagents and Tools Table, Materials and Methods, Figures, Data Availability Section)
New materials and reagents need to be available; do any restrictions apply?	Not Applicable	

Antibodies	Information included in the manuscript?	In which section is the information available? (Reagents and Tools Table, Materials and Methods, Figures, Data Availability Section)
For **antibodies** provide the following information:<br>- Commercial antibodies: RRID (if possible) or supplier name, catalogue number and or/clone number<br>- Non-commercial: RRID or citation	Not Applicable	

DNA and RNA sequences	Information included in the manuscript?	In which section is the information available? (Reagents and Tools Table, Materials and Methods, Figures, Data Availability Section)
**Short novel DNA or RNA including primers, probes**: provide the sequences.	Not Applicable	

Cell materials	Information included in the manuscript?	In which section is the information available? (Reagents and Tools Table, Materials and Methods, Figures, Data Availability Section)
**Cell lines:** Provide species information, strain. Provide accession number in repository **OR** supplier name, catalog number, clone number, and/**OR** RRID.	Yes	Methods
**Primary cultures:** Provide species, strain, sex of origin, genetic modification status.	Not Applicable	
Report if the cell lines were recently **authenticated** (e.g., by STR profiling) and tested for mycoplasma contamination.	Yes	Methods

Experimental animals	Information included in the manuscript?	In which section is the information available? (Reagents and Tools Table, Materials and Methods, Figures, Data Availability Section)
**Laboratory animals or Model organisms:** Provide species, strain, sex, age, genetic modification status. Provide accession number in repository **OR** supplier name, catalog number, clone number, **OR** RRID.	Yes	Methods
**Animal observed in or captured from the field:** Provide species, sex, and age where possible.	Not Applicable	
Please detail **housing and husbandry conditions**.	Not Applicable	

Plants and microbes	Information included in the manuscript?	In which section is the information available? (Reagents and Tools Table, Materials and Methods, Figures, Data Availability Section)
**Plants:** provide species and strain, ecotype and cultivar where relevant, unique accession number if available, and source (including location for collected wild specimens).	Not Applicable	
**Microbes:** provide species and strain, unique accession number if available, and source.	Not Applicable	

Human research participants	Information included in the manuscript?	In which section is the information available? (Reagents and Tools Table, Materials and Methods, Figures, Data Availability Section)
If collected and within the bounds of privacy constraints report on age, sex and gender or ethnicity for all study participants.	Not Applicable	

Core facilities	Information included in the manuscript?	In which section is the information available? (Reagents and Tools Table, Materials and Methods, Figures, Data Availability Section)
If your work benefited from core facilities, was their service mentioned in the acknowledgments section?	Yes	Acknowledgement

Design

Study protocol	Information included in the manuscript?	In which section is the information available? (Reagents and Tools Table, Materials and Methods, Figures, Data Availability Section)
If study protocol has been **pre-registered, provide DOI in the manuscript**. For clinical trials, provide the trial registration number **OR** cite DOI.	Not Applicable	
Report the **clinical trial registration number** (at ClinicalTrials.gov or equivalent), where applicable.	Not Applicable	

Laboratory protocol	Information included in the manuscript?	In which section is the information available? (Reagents and Tools Table, Materials and Methods, Figures, Data Availability Section)
Provide DOI OR other citation details if **external detailed step-by-step protocols** are available.	Not Applicable	

Experimental study design and statistics	Information included in the manuscript?	In which section is the information available? (Reagents and Tools Table, Materials and Methods, Figures, Data Availability Section)
Include a statement about **sample size** estimate even if no statistical methods were used.	Yes	Main text, Methods
Were any steps taken to minimize the effects of subjective bias when allocating animals/samples to treatment (e.g. **randomization procedure**)? If yes, have they been described?	Yes	Main text, Methods
Include a statement about **blinding** even if no blinding was done.	Not Applicable	
Describe **inclusion/exclusion criteria** if samples or animals were excluded from the analysis. Were the criteria pre-established?<br><br>If sample or data points were omitted from analysis, report if this was due to attrition or intentional exclusion and provide justification.	Yes	Main text, Methods
For every figure, are **statistical tests** justified as appropriate? Do the data meet the assumptions of the tests (e.g., normal distribution)? Describe any methods used to assess it. Is there an estimate of variation within each group of data? Is the variance similar between the groups that are being statistically compared?	Yes	Main text, Methods

Sample definition and in-laboratory replication	Information included in the manuscript?	In which section is the information available? (Reagents and Tools Table, Materials and Methods, Figures, Data Availability Section)
In the figure legends: state number of times the experiment was **replicated** in laboratory.	Not Applicable	
In the figure legends: define whether data describe **technical or biological replicates**.	Yes	Main text

Ethics

Ethics	Information included in the manuscript?	In which section is the information available? (Reagents and Tools Table, Materials and Methods, Figures, Data Availability Section)
Studies involving **human participants**: State details of **authority granting ethics approval** (IRB or equivalent committee(s), provide reference number for approval.	Not Applicable	
Studies involving **human participants**: Include a statement confirming that **informed consent** was obtained from all subjects and that the experiments conformed to the principles set out in the WMA Declaration of Helsinki and the Department of Health and Human Services Belmont Report.	Not Applicable	
Studies involving **human participants:** For publication of **patient photos**, include a statement confirming that consent to publish was obtained.	Not Applicable	
Studies involving experimental **animals**: State details of **authority granting ethics approva**l (IRB or equivalent committee(s), provide reference number for approval. Include a statement of compliance with ethical regulations.	Not Applicable	
Studies involving **specimen and field samples:** State if relevant **permits** obtained, provide details of authority approving study; if none were required, explain why.	Not Applicable	

Dual Use Research of Concern (DURC)	Information included in the manuscript?	In which section is the information available? (Reagents and Tools Table, Materials and Methods, Figures, Data Availability Section)
Could your study fall under dual use research restrictions? Please check biosecurity documents and list of **select agents and toxins** (CDC): https://www.selectagents.gov/sat/list.htm	Not Applicable	
If you used a select agent, is the security level of the lab appropriate and reported in the manuscript?	Not Applicable	
If a study is subject to dual use research of concern regulations, is the name of the **authority granting approval and reference number** for the regulatory approval provided in the manuscript?	Not Applicable	

Reporting

The MDAR framework recommends adoption of discipline-specific guidelines, established and endorsed through community initiatives. Journals have their own policy about requiring specific guidelines and recommendations to complement MDAR.

Adherence to community standards	Information included in the manuscript?	In which section is the information available? (Reagents and Tools Table, Materials and Methods, Figures, Data Availability Section)
State if relevant guidelines or checklists (e.g., **ICMJE, MIBBI, ARRIVE, PRISMA**) have been followed or provided.	Not Applicable	
For **tumor marker prognostic studies,** we recommend that you follow the **REMARK** reporting guidelines (see link list at top right). See author guidelines, under 'Reporting Guidelines'. Please confirm you have followed these guidelines.	Not Applicable	
For **phase II and III randomized controlled trials**, please refer to the **CONSORT** flow diagram (see link list at top right) and submit the CONSORT checklist (see link list at top right) with your submission. See author guidelines, under 'Reporting Guidelines'. Please confirm you have submitted this list.	Not Applicable	

Data Availability

Data availability	Information included in the manuscript?	In which section is the information available? (Reagents and Tools Table, Materials and Methods, Figures, Data Availability Section)
Have **primary datasets** been deposited according to the journal's guidelines (see 'Data Deposition' section) and the respective accession numbers provided in the Data Availability Section?	Yes	Data and code availibility
Were **human clinical and genomic datasets** deposited in a public access-controlled repository in accordance to ethical obligations to the patients and to the applicable consent agreement?	Not Applicable	Methods
Are **computational models** that are central and integral to a study available without restrictions in a machine-readable form? Were the relevant accession numbers or links provided?	Yes	Data and code availibility
If publicly available data were reused, provide the respective **data citations in the reference list**.	Yes	Methods
